# Stability against fluctuations: a two-dimensional study of scaling, bifurcations and spontaneous symmetry breaking in stochastic models of synaptic plasticity

**DOI:** 10.1007/s00422-024-00985-0

**Published:** 2024-04-07

**Authors:** Terry Elliott

**Affiliations:** Department of Electronics and Computer Science, University of Southampton, Highfield, Southampton, SO17 1BJ UK

**Keywords:** Synaptic plasticity, Neuronal development, Stochastic processes, Fluctuations

## Abstract

Stochastic models of synaptic plasticity must confront the corrosive influence of fluctuations in synaptic strength on patterns of synaptic connectivity. To solve this problem, we have proposed that synapses act as filters, integrating plasticity induction signals and expressing changes in synaptic strength only upon reaching filter threshold. Our earlier analytical study calculated the lifetimes of quasi-stable patterns of synaptic connectivity with synaptic filtering. We showed that the plasticity step size in a stochastic model of spike-timing-dependent plasticity (STDP) acts as a temperature-like parameter, exhibiting a critical value below which neuronal structure formation occurs. The filter threshold scales this temperature-like parameter downwards, cooling the dynamics and enhancing stability. A key step in this calculation was a resetting approximation, essentially reducing the dynamics to one-dimensional processes. Here, we revisit our earlier study to examine this resetting approximation, with the aim of understanding in detail why it works so well by comparing it, and a simpler approximation, to the system’s full dynamics consisting of various embedded two-dimensional processes without resetting. Comparing the full system to the simpler approximation, to our original resetting approximation, and to a one-afferent system, we show that their equilibrium distributions of synaptic strengths and critical plasticity step sizes are all qualitatively similar, and increasingly quantitatively similar as the filter threshold increases. This increasing similarity is due to the decorrelation in changes in synaptic strength between different afferents caused by our STDP model, and the amplification of this decorrelation with larger synaptic filters.

## Introduction

Spike-timing-dependent plasticity (STDP; Markram et al. 1997; Bi and Poo 1998; Zhang et al. 1998; Froemke and Dan 2002; Roberts and Bell 2002; Harvey and Svoboda 2007; Caporale and Dan 2008)—the biphasic, graded change in synaptic efficacy depending on the relative timing of pre- and postsynaptic spiking—is typically understood to imply that single synapses can express finely graded changes in synaptic strength, with this assumption implicit in many models (Song et al. 2000; van Rossum et al. 2000; Castellani et al. 2001; Senn et al. 2001; Sjöström and Nelson 2002; Burkitt et al. 2004; Bi and Rubin 2005; Rubin et al. 2005; Bender et al. 2006). Yet some experimental evidence suggests that synapses may occupy only discrete states of synaptic strength (Montgomery and Madison 2002, 2004; O’Connor et al. 2005a, b; Bartol et al. 2015) or may change their strengths only in discrete, all-or-none jumps (Petersen et al. 1998; Yasuda et al. 2003; Bagal et al. 2005; Sobczyk and Svoboda 2007). One way to resolve this apparent contradiction is to propose that a single synapse may express only fixed-amplitude steps in synaptic strength, with the probability but not amplitude of change depending on spike timing (Appleby and Elliott 2005). The classic, graded biphasic STDP curve (Bi and Poo 1998) then emerges as an average change over multiple synapses for a single spike-pair presentation or at a single synapse over multiple spike-pair presentations (Appleby and Elliott 2005).

Any probabilistic or stochastic model of synaptic plasticity faces the challenge posed by destabilising fluctuations in synaptic strength. When considering, for example, neuronal development (Purves and Lichtman 1985), fluctuations can lead to a change in the patterns of synaptic connectivity acquired through activity-dependent competitive dynamics in the developing primary visual cortex (V1). In particular, the segregated afferent input to V1 neurons, in which one eye or the other dominates in the control of V1 neurons (Hubel and Wiesel 1962), can be destabilised by fluctuations, so that the two afferents repeatedly switch control of a target cell over some characteristic, average time scale (Appleby and Elliott 2006; Elliott 2008). Reducing the size of the all-or-none steps in synaptic strength can control these fluctuations, but if the plasticity step size must be very small to control fluctuations, then models become biologically implausible and, furthermore, synaptic strengths become for all practical purposes graded rather than discrete (Elliott 2008). We have instead proposed that synapses act as low-pass filters, integrating their plasticity induction signals before expressing a step change in synaptic strength (Elliott 2008; Elliott and Lagogiannis 2009). These “integrate-and-express” models powerfully control fluctuations without having to resort to implausible parameter choices or assumptions.

Previously we extensively analysed three such models of synaptic filtering operating in concert with our model of STDP (Elliott 2011b). We found that the plasticity step size plays the role of a temperature-like parameter, with smaller step sizes corresponding to lower temperatures. Structure formation (segregated states) emerges only below a critical plasticity step size, akin to a Curie point or critical temperature. Synaptic filtering “cools” the dynamics, with the filter threshold scaling back the actual plasticity step size into an effective step size, where this scaling is linear over a range of parameters. This analysis was performed using the master equation for the strengths of two afferents (representing inputs via the lateral geniculate nucleus from the two eyes) and its Fokker–Planck equation limit. In order to write down the master equation, it was necessary to make several approximations, but the key one for the purposes of analytical tractability was to assume that the occurrence of a plasticity step in one afferent resets the filter states in all afferents. This resetting or renewal approximation essentially reduces a random walk in multiple dimensions (afferents) to a set of one-dimensional random walks, one for each afferent. The afferents’ dynamics are then coupled only through the common postsynaptic firing rate of their target cell. We argued although did not demonstrate that this approximation works because our stochastic model of STDP, together with synaptic filtering, decorrelates synaptic strength changes across multiple afferents, with simultaneous changes becoming increasingly unlikely with larger filter thresholds.

Using this resetting or renewal approximation is essential in obtaining analytical results that are key to understanding the stability and lifetimes of patterns of neuronal connectivity acquired during development, and thus to revealing the role of the plasticity step size as a temperature-like parameter. Understanding in detail why this approximation works so well is therefore of central importance, not just to our own models but to other stochastic models in which this approximation could be employed. Our purpose here is thus to examine this approximation in detail by comparing and contrasting results obtained with it to those obtained from the full synaptic dynamics without it, and to those obtained with other, simpler approximations. Doing so shows when and why the approximation works. To achieve this, we consider the full dynamics of two afferents in their joint STDP and filter state space, obtaining the equilibrium joint probability distribution for their strengths. This distribution determines whether or not segregated states of afferent connectivity exist as the outcome of the process of neuronal development. We compare these full results to those from our earlier study and to the results from a model that may be regarded as intermediate between the full and earlier models. Regardless of which of these three models we use, all results are qualitatively and often quantitatively very similar, especially for larger filters, confirming the validity of the central approximation used in our earlier model and verifying our explanation of it in terms of decorrelating changes in synaptic strength. We also compare some of our results to simulation, particularly in relation to the lifetimes of segregated states of afferent connectivity. Most of our results must be obtained by the numerical solution of large, two-dimensional linear systems, so we restrict to a study of just the simplest possible filter model. However, the validation of our earlier key approximation by these numerical methods permits the full analytical power of that approximation to be deployed.

The remainder of our paper is organised as follows: In the next section, we introduce in some detail the methods and approach that we take for a single afferent (or synapse). Section 3 then builds on these methods to consider two afferents, extending the machinery of Sect. 2 to the three models that we examine. Then in Sect. 4, we present our numerical and simulation results for these models, comparing and contrasting them. Finally, in Sect. 5 we briefly discuss our results.

## Recapitulation of synaptic dynamics of one afferent

Before considering two or more afferents in the next section, we first consider our previous analysis (Elliott 2011b) of the dynamics of one afferent synapsing on a target cell. We do this in some detail to provide orientation; to set up the formalism and notation; to describe our switch-based model of STDP for synaptic plasticity induction and our simplest filter-based model for synaptic plasticity expression; to clarify conceptual issues that previously were implicit or unclear; and to extend some of our previous results from specific to general cases. Mathematically and notationally speaking, the two-afferent case considered in Sect. 3 is much more elaborate and involved than the one-afferent case considered in this section. We therefore also discuss the one-afferent case at some length so that the major issues and approximations involved are behind us when we turn to the mathematically harder but conceptually identical two-afferent case.

### Plasticity induction by tristate STDP switch

**Fig. 1:**
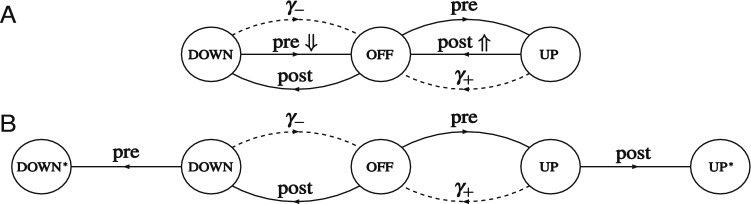
Synaptic state transitions induced by spike-timing-dependent plasticity in the tristate switch model. Circles indicate STDP-related synaptic states and either solid or dashed lines transitions between them. Solid lines labelled “pre” or “post” represent transitions caused by pre- or postsynaptic spikes, respectively, while dashed lines labelled “$\gamma _+$” or “$\gamma _-$” represent stochastic decay processes. Lines additionally labelled “$\Uparrow$” or “$\Downarrow$” indicate transitions in which synaptic plasticity (potentiation or depression, respectively) is induced. In panel A, the return of the tristate switch to the OFF state after each plasticity-inducing transition is explicitly represented. In panel B, we instead replace these plasticity-inducing transitions with transitions into fictitious absorbing states that enable the calculation of first passage time densities for plasticity induction processes

Our tristate switch model of STDP (Appleby and Elliott 2005) postulates that any given synapse resides in one of three STDP-related synaptic states, as illustrated in Fig. 1A. We refer to these states as the “UP” state, the “OFF” state, and the “DOWN” state.^1^ Transitions between these three states are driven by pre- or postsynaptic spikes, as well as by two stochastic decay processes. When in the OFF state, the occurrence of a presynaptic spike will drive the synapse to the UP state, while a postsynaptic spike will cause a transition to the DOWN state. From the UP state, a postsynaptic spike will drive the synapse back to the OFF state, inducing a fixed-amplitude increment of $T_+$ in synaptic strength. The sequence of transitions OFF $\rightarrow$ UP $\rightarrow$ OFF driven by a presynaptic followed by postsynaptic spike pair accounts for the potentiating side of the usual biphasic STDP curve (Bi and Poo 1998) although not the dependence of the amplitude on the spike-time difference. Similarly, when in the DOWN state, a presynaptic spike triggers a transition back to the OFF state, inducing a fixed-amplitude decrement of $T_-$ in synaptic strength. The sequence of transitions OFF $\rightarrow$ DOWN $\rightarrow$ OFF driven by a postsynaptic followed by a presynaptic spike pair accounts for the depressing side of the usual STDP curve, although again not the dependence of the amplitude on spike-time difference.

To account for the spike-time dependence in both cases, we suppose that a synapse in the UP state will stochastically decay back to the OFF state, and similarly a synapse in the DOWN state will stochastically decay back to the OFF state. Although a single spike pair (either presynaptic followed by postsynaptic or postsynaptic followed by presynaptic) will always induce a fixed-amplitude jump (or no change) in synaptic strength, multiple such spike pairs at the same synapse, or the same spike pair across different synapses between the same pre- and postsynaptic neuron, will then induce graded changes in synaptic strength (at the same synapse or across all synapses between afferent and target). This is because the stochastic decay processes are assumed to be independent between different spike-pair instances at the same synapse or for the same spike-pair instance across different synapses. For example, a 10-ms time difference between a presynaptic spike followed by a postsynaptic spike may on one occasion induce a potentiation step because the postsynaptic spike occurs before the decay process, but on another may not because the decay process occurs first. Although the two stochastic decay processes could be characterised by any PDFs, for simplicity we take them to be exponential distributions with rate $\lambda _+$ for the UP $\rightarrow$ OFF decay process and $\lambda _-$ for the DOWN $\rightarrow$ OFF decay process. These decays rates $\lambda _\pm$ translate into time scales $\tau _\pm = 1 / \lambda _\pm$ characterising the widths of the two sides of the biphasic STDP window.

We have not indicated how a synapse in the UP state responds to a presynaptic spike, nor how a synapse in the DOWN state responds to a postsynaptic spike. Variants of the basic tristate switch model can be constructed depending on whether or not a spike of the appropriate type resets the stochastic decay process of the corresponding state (Appleby and Elliott 2006). Here we consider the simplest and most natural model, which assumes that a synapse in the UP state does not respond at all to a presynaptic spike, and similarly for the DOWN state and a postsynaptic spike. However, when all inter-event processes are exponentially distributed, the memorylessness of the exponential process ensures that all these variant models collapse down to one model.

It is therefore convenient to assume that pre- and postsynaptic spike processes are governed by Poisson processes of rates that we write for brevity as $\lambda _\pi$ and $\lambda _p$, respectively. Inter-spike intervals for the same spike type are then exponentially distributed. With the assumption of Poisson spiking, we may show that the parameter $\gamma = (T_+ \lambda _-)/(T_- \lambda _+) = (T_+ \tau _+) / (T_- \tau _-)$ must satisfy the condition $\gamma < 1$ to prevent potentiation dominating depression (Appleby and Elliott 2005). Furthermore, we may also show that activity-dependent competitive dynamics naturally arise between different afferents synapsing on the same target cell for particular ranges of $\gamma$ (Appleby and Elliott 2006; Elliott 2008). Moreover, these competitive dynamics depend on higher-order spike interactions (e.g. spike triplets and quadruplets) that our STDP model automatically accommodates without additional postulates (Appleby and Elliott 2005, 2007). We will set $T_+ = T_-$ so that synaptic strengths occupy points on a lattice, and previously we used the choice $\gamma = 3/5$ with $T_\pm = T$ to ensure the presence of competitive synaptic dynamics (Elliott 2011b). We take $\lambda _- = 50$ Hz (or $\tau _- = 20$ ms) as a standard choice from this earlier work, which therefore implies that $\lambda _+ \approx 83$ Hz (or $\tau _+ = 12$ ms).

We have been careful to label the transitions in Fig. 1A according to the processes that cause them rather than according to the rates at which they occur. This is because a plasticity induction signal that is generated by the spike-driven UP $\rightarrow$ OFF or DOWN $\rightarrow$ OFF transition will trigger a change in the synapse’s state, whether that be its strength or some other, internal synaptic state. Even for a fixed presynaptic spike firing rate $\lambda _\pi$, if an induction signal causes an immediate change in synaptic strength, then the postsynaptic spike firing rate $\lambda _p$ will change. Hence, the rates of the transitions in Fig. 1A may change because of the induction processes triggered by these very transitions, even when $\lambda _\pi$ is fixed. The induction processes are formally first passage time (FPT) processes in which, starting from any given STDP switch state at time $t = 0$ s, a postsynaptic-spike-driven UP $\rightarrow$ OFF transition occurs for the first time, or a presynaptic-spike-driven DOWN $\rightarrow$ OFF transition occurs for the first time, with in each case an induction event of the other type having not already occurred. Prior to an induction signal being generated for the first time, the rates of the processes in Fig. 1A cannot change unless $\lambda _\pi$ changes. Once an induction signal has been generated, the rate $\lambda _p$ could change even if $\lambda _\pi$ does not. We must therefore focus on the FPT processes by which the STDP switch mechanism generates plasticity induction signals, as these signals drive further synaptic changes.

To compute the FPT densities for the induction signals generated by the tristate STDP switch, we introduce the fictitious absorbing states $\hbox {UP}^*$ and $\hbox {DOWN}^*$ and replace the spike-driven transitions UP $\rightarrow$ OFF and DOWN $\rightarrow$ OFF in Fig. 1A with the transitions UP $\rightarrow$ $\hbox {UP}^*$ and DOWN $\rightarrow$ $\hbox {DOWN}^*$, respectively, shown in Fig. 1B. In Fig. 1B, any transitions between the standard DOWN, OFF and UP switch states are then by construction transitions that have occurred without an induction signal having been generated. However, probability will accumulate in the fictitious absorbing states over time, with the rate of change of the probabilities of these two states corresponding precisely to the FPT densities for the occurrence of plasticity induction signals. Ordering the switch states including the fictitious absorbing states as $\{ \textrm{DOWN}^*, \textrm{DOWN}, \textrm{OFF}, \textrm{UP}, \textrm{UP}^*\}$, we first define the $3 \times 3$ matrix

1 $$\begin{aligned} \mathbb {S}_0 = \begin{pmatrix} - (\lambda _- + \lambda _\pi ) & \lambda _p & 0 \\ \lambda _- & - (\lambda _\pi + \lambda _p) & \lambda _+ \\ 0 & \lambda _\pi & - (\lambda _+ + \lambda _p) \end{pmatrix} \end{aligned}$$

as the generating matrix for transitions purely within the $\{ \textrm{DOWN}, \textrm{OFF}, \textrm{UP} \}$ states excluding transitions to the fictitious states. Defining also the two vectors

2 $$\begin{aligned} \underline{v}^+ = \begin{pmatrix} 0 \\ 0 \\ \lambda _p \end{pmatrix} \, \, \, \, \text{ and } \, \, \, \, \underline{v}^- = \begin{pmatrix} \lambda _\pi \\ 0 \\ 0 \end{pmatrix}, \end{aligned}$$

the $5 \times 5$ generating matrix for all transitions including those to the fictitious states is then given by3We have written the matrix in schematic block form, so the central three elements of the first and last rows contain the components of $\underline{v}^-$ and $\underline{v}^+$, respectively; the central $3 \times 3$ submatrix is just $\mathbb {S}_0$; all elements of the first and last columns are zero; the vector $\underline{0}$ denotes a vector of zeros (in this case with three components); and a superscript $\textrm{T}$ denotes the transpose.

The transition matrix $\mathbb {P}(t)$ that describes the transition probabilities between the five states in Fig. 1B in a time *t* is then the solution of the standard forward Chapman–Kolmogorov equation $\textrm{d} \mathbb {P}(t)/\textrm{d}t = \mathbb {S}_*\mathbb {P}(t)$, subject to the initial condition $\mathbb {P} (0) = \mathbb {I}$, with $\mathbb {I}$ being the identity matrix. If $\lambda _\pi$ and thus $\lambda _p$ are constant, then we obtain the standard matrix exponential solution $\mathbb {P}(t) = \exp \left( \mathbb {S}_*\, t \right)$. It is easy to see that the matrix exponential in this case takes the form4where the integral terms arise by simplifying the sum $\sum _{n=1}^\infty \mathbb {S}_0^{n-1} \, (t^n/n!)$. If $\lambda _\pi$ and thus $\lambda _p$ are not constant, then the matrix exponentials in Eq. (4) are replaced throughout by time-ordered matrix exponentials. Indexing the standard STDP switch states with letters such as *X* and *Y*, where $X, Y \in \{ \textrm{DOWN}, \textrm{OFF}, \textrm{UP} \}$, the FPT densities $G_Y^\pm (t)$ for potentiating and depressing induction signals at time *t* starting from switch state *Y* are given by:

5a $$\begin{aligned} G_Y^-(t)&= \frac{\textrm{d}}{\textrm{d} t} \left[ \, \mathbb {P}(t) \right] _{\textrm{DOWN}^*,Y}, \end{aligned}$$

5b $$\begin{aligned} G_Y^+(t)&= \frac{\textrm{d}}{\textrm{d} t} \left[ \, \mathbb {P}(t) \right] _{\textrm{UP}^*,Y}, \end{aligned}$$

where we index matrix elements according to the associated states rather than numerically. Hence, writing the vectors $\underline{G}^\pm (t) = \left( G_\textrm{DOWN}^\pm (t), G_\textrm{OFF}^\pm (t), G_\textrm{UP}^\pm (t) \right) ^{\textrm{T}}$, we have

6 $$\begin{aligned} \underline{G}^\pm (t) = \exp \left( \mathbb {S}_0^{\textrm{T}} \, t \right) \, \underline{v}^\pm . \end{aligned}$$

We must have that $G_Y^\pm (0) \equiv 0$ because an induction signal cannot be generated if no time has elapsed, so Eq. (6) is valid for $t > 0$ s and not at $t = 0$ s. We may confirm that the FPT densities satisfy the differential equations

7 $$\begin{aligned} \frac{\textrm{d} \underline{G}^\pm (t)}{\textrm{d} t} = \mathbb {S}_0^{\textrm{T}} \, \underline{G}^\pm (t) + \delta (t) \, \underline{v}^\pm , \end{aligned}$$

subject to these initial conditions, $\underline{G}^\pm (0) = \underline{0}$. The Dirac delta function $\delta (t)$ gives the discontinuous behaviours of the $G_Y^\pm (t)$ at $t = 0$ s. The inhomogeneous terms in Eq. (7) correspond to absorbing boundary conditions that act via $\delta (t)$ at the instant that a transition to the relevant fictitious absorbing state occurs. The appearance of the transposed matrix $\mathbb {S}_0^{\textrm{T}}$ rather than $\mathbb {S}_0$ in Eq. (7) reflects the fact that FPT processes must be computed by keeping the final state (here $\hbox {DOWN}^*$ or $\hbox {UP}^*$ in Eq. (5)) fixed and allowing a variable initial state *Y*, rather than *vice versa*. Hence, they arise from the backward rather than the forward Chapman-Kolmogorov equation, with backward processes indicated by post- rather than pre-multiplication by $\mathbb {S}_0$. As $\underline{G}^\pm (t)$ are vectors and not matrices, post-multiplication by $\mathbb {S}_0$ must become pre-multiplication by its transpose, $\mathbb {S}_0^{\textrm{T}}$.

Equation (7) may alternatively be written down by other, standard methods (see, e.g. van Kampen 1992). However, by using the fictitious absorbing states and the matrix $\mathbb {S}_*$, we can just read off from the matrix $\mathbb {P}(t)$ not only the FPT densities $G_Y^\pm (t)$ but also the transition probabilities between the standard switch states $\{ \textrm{DOWN}, \textrm{OFF}, \textrm{UP} \}$ without a transition to the fictitious absorbing states $\{ \textrm{DOWN}^*, \textrm{UP}^*\}$ having occurred. We represent these by the elements of a matrix $\mathbb {F}(t)$, where $\mathbb {F}(t)$ is just the central $3 \times 3$ submatrix of $\mathbb {P}(t)$, so

8 $$\begin{aligned} \mathbb {F}(t) = \exp \left( \mathbb {S}_0 \, t \right) \end{aligned}$$

or its time-ordered equivalent. We write its elements as $F_Y^X(t) = \left[ \, \mathbb {F}(t) \right] _{X,Y}$, for the transition probability from switch state *Y* to switch state *X* in time *t*, without a transition to $\hbox {DOWN}^*$ or $\hbox {UP}^*$ having occurred. Asymptotically these transition probabilities must vanish, so $F_Y^X(t) \rightarrow 0$ as $t \rightarrow \infty$, because an induction process is inevitable given enough time: all probability finally accumulates in the top and bottom rows of $\mathbb {P}(t)$. The matrix elements $F_Y^X(t)$ and the FPT densities $G_Y^\pm (t)$ are related by the equality:

9a $$\begin{aligned} \sum _X F_Y^X(t)&= 1 - \int _0^t \textrm{d} \tau \left[ G_Y^+ (\tau ) + G_Y^- (\tau ) \right] \end{aligned}$$

with the sum running over $X \in \{ \textrm{DOWN}, \textrm{OFF}, \textrm{UP} \}$, because the left-hand side is the probability of having not transitioned from the three standard switch states to the fictitious absorbing states, while the right-hand side is the complement of the probability of having already been absorbed by them. This probability is just the waiting time distribution for either of these FPT processes to occur. Alternatively, Eq. (9a) is just the statement that each column sum of $\mathbb {P}(t)$ must be unity. The further equalities

9b $$\begin{aligned} G_Y^- (t)&= \lambda _\pi \, F_Y^{\textrm{DOWN}} (t), \end{aligned}$$

9c $$\begin{aligned} G_Y^+ (t)&= \lambda _p \, F_Y^{\textrm{UP}} (t), \end{aligned}$$

follow directly from Eqs. (4), (5) and (8).

We will require the rates of the FPT processes defined by the pair of mutually exclusive density functions $G_Y^\pm (t)$ for any given *Y*, considered purely in mathematical terms rather than as processes that arise from some underlying mechanism. The rates of these processes are determined from the probability distribution for the number of occurrences of these events in some time *t*, where immediately upon the occurrence of one of them, we start waiting for the next. Let $N(\nu _+, \nu _-; t)$ be the probability of $\nu _\pm$ occurrences of the events associated with the densities $G_Y^\pm (t)$, respectively. Then, we have

10 $$\begin{aligned}&N(\nu _+, \nu _-; t) \nonumber \\&\quad = \delta _{\nu _+,0} \, \delta _{\nu _-,0} \, \left\{ 1 - \int _0^t \textrm{d} \tau \, \left[ G_Y^+(\tau ) + G_Y^-(\tau ) \right] \right\} \nonumber \\&\qquad + \int _0^t \textrm{d} \tau \, \big [ N(\nu _+ {-} 1, \nu _-; t {-} \tau ) \, G_Y^+(\tau ) \nonumber \\&\qquad + \, N(\nu _+, \nu _- {-} 1; t {-} \tau ) \, G_Y^-(\tau ) \big ], \end{aligned}$$

for $\nu _\pm \ge 0$ with boundary conditions $N(-1, \nu _-; t) = 0$ and $N(\nu _+, -1; t) = 0$, where $\delta _{a,b}$ is the Kronecker delta symbol. The inhomogeneous term on the right-hand side involves the waiting time distribution for either event. If neither has occurred in time *t*, then $\nu _\pm$ must both be zero with this waiting time probability. The homogeneous terms essentially count the events as they first occur at some time $\tau$, with further events having to occur in the remaining time $t - \tau$ to give the required number of events $\nu _\pm$ at time *t*. By taking the Laplace transform, with $\widehat{f}(s)$ denoting the transform of *f*(*t*), and defining the probability generating function (PGF)

11 $$\begin{aligned} \mathscr {G}(x, y; t) = \sum _{\nu _\pm = 0}^{\infty } x^{\nu _+} y^{\nu _-} N(\nu _+, \nu _-; t), \end{aligned}$$

we obtain

12 $$\begin{aligned} \widehat{\mathscr {G}}(x, y; s) = \frac{1}{s} \, \frac{1 - \widehat{G}_Y^+(s) - \widehat{G}_Y^+(s)}{1 - x \, \widehat{G}_Y^+(s) - y \, \widehat{G}_Y^+(s)}. \end{aligned}$$

The rates $r_Y^\pm (t)$ of the induction signals are just the derivatives of the expectation values of $\nu _\pm$. It is more convenient to consider the derivatives of these rates, so $\rho _Y^\pm (t) = \textrm{d} r_Y^\pm (t)/\textrm{d}t$. Since $\widehat{\rho }_Y^\pm (s) = s \, \widehat{r}_Y^{\, \pm } (s)$, where we have used the fact that $r_Y^\pm (0) = 0$ Hz (cf. $G_Y^\pm (0) = 0$), we get

13 $$\begin{aligned} \widehat{\rho }_Y^\pm (s) = \frac{s \, \widehat{G}_Y^\pm (s)}{1 - \widehat{G}_Y^+(s) - \widehat{G}_Y^-(s)} \end{aligned}$$

with inverse relations

14 $$\begin{aligned} \widehat{G}_Y^\pm (s) = \frac{\widehat{\rho }_Y^\pm (s)}{s + \widehat{\rho }_Y^+(s) + \widehat{\rho }_Y^-(s)}. \end{aligned}$$

For any escape or FPT processes, we always obtain this relationship between the $\widehat{\rho }$’s and the $\widehat{G}$’s, but with the denominators extended to sums over all exit processes into all possible absorbing states. The rate derivatives $\rho _Y^\pm (t)$ are useful because we can obtain the asymptotic behaviour of the rates $r_Y^\pm (t)$ from the Laplace transforms of $\rho _Y^\pm (t)$. In particular,

15 $$\begin{aligned} \widehat{\rho }_Y^\pm (0) \equiv \int _0^\infty \textrm{d} \tau \, \rho _Y^\pm (\tau ) = \int _0^\infty \textrm{d} \tau \, \frac{\textrm{d} r_Y^\pm (\tau )}{\textrm{d} \tau } = \lim _{t \rightarrow \infty } r_Y^\pm (t), \end{aligned}$$

using $r_Y^\pm (0) \equiv 0$ Hz. As $\widehat{\rho }_Y^\pm (s) = s \, \widehat{r}_Y^{\, \pm } (s)$, this result is an example of a standard Tauberian theorem, namely that $\lim _{s \rightarrow 0} s \, \widehat{f} (s) = \lim _{t \rightarrow \infty } f(t)$ (see, e.g. Feller 1967, vol. II).

Before considering how a synapse might respond to the plasticity induction signals generated by the STDP switch, it is instructive to reconsider the dynamics in Fig. 1A. Although we have argued that the spike-driven UP $\rightarrow$ OFF and DOWN $\rightarrow$ OFF transitions can lead to changes in the rates of these very transitions because of synaptic plasticity, we can instead consistently define the system in Fig. 1A by considering the scenario in which a synapse just ignores plasticity induction signals. The rates of the transitions in Fig. 1A may then be time-dependent only in virtue of any time-dependence of the presynaptic spike firing rate and not because of any changes that the transitions in Fig. 1A may themselves trigger. The generating matrix for the transitions in Fig. 1A is then given by:

16 $$\begin{aligned} \mathbb {S} = \begin{pmatrix} - (\lambda _- + \lambda _\pi ) & \lambda _p & 0 \\ \lambda _- + \lambda _\pi & - (\lambda _\pi + \lambda _p) & \lambda _++ \lambda _p \\ 0 & \lambda _\pi & - (\lambda _+ + \lambda _p) \end{pmatrix}, \end{aligned}$$

which is just the matrix $\mathbb {S}_0$ with the spike-driven UP $\rightarrow$ OFF and DOWN $\rightarrow$ OFF transitions included. If the presynaptic spike firing rate $\lambda _\pi$ and thus the postsynaptic spike firing rate $\lambda _p$ do not depend on time, then we may examine the equilibrium distribution of the STDP switch states. Denoting this distribution by the vector $\underline{\sigma }$ with components $\sigma _X$, $X \in \{ \textrm{DOWN}, \textrm{OFF}, \textrm{UP} \}$, $\underline{\sigma }$ is the normalised null right eigenvector of $\mathbb {S}$, so that $\mathbb {S} \, \underline{\sigma } = \underline{0}$, where $\underline{\sigma } \cdot \underline{1} = 1$, with $\underline{1}$ being a vector of unit components and the dot denotes the dot product. In equilibrium, the rate $r^+$ of potentiating induction signals is just the probability that the STDP switch is in the UP state multiplied by the rate $\lambda _p$ of the postsynaptic-spike-driven UP $\rightarrow$ OFF transition. Thus, $r^+ = \lambda _p \, \sigma _{\, \textrm{UP}}$. Similarly, $r^- = \lambda _\pi \, \sigma _{\, \textrm{DOWN}}$. These equilibrium rates $r^\pm$ in fact agree precisely with an explicit calculation of the asymptotic rates $r_{\textrm{OFF}}^\pm (\infty ) = \widehat{\rho }^\pm _{\textrm{OFF}} (0)$ via the FPT densities $G_{\textrm{OFF}}^\pm (t)$ discussed above, when the spike firing rates $\lambda _\pi$ and $\lambda _p$ are of course taken to be fixed. Notice, however, that $r_{\textrm{OFF}}^\pm (\infty )$ are by construction the asymptotic rates of FPT induction signals specifically starting from an initial OFF state. The dependence of the rates $r_Y^\pm (t)$ on their initial state *Y* is in general never lost, not even in the asymptotic limit when it exists (e.g. $r_{\textrm{DOWN}}^+(\infty ) \ne r_{\textrm{OFF}}^+(\infty )$), whereas essentially by definition the equilibrium vector $\underline{\sigma }$ retains no memory of the initial distribution of the switch states. The use of an equilibrium distribution to calculate particular asymptotic FPT induction rates in this way provides a powerful and simple tool that will be extremely useful later.

### Synaptic filtering of plasticity induction signals

**Fig. 2:**
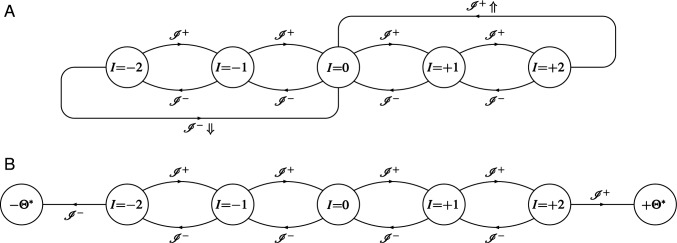
Changes in synaptic filter state triggered by the synaptic plasticity induction signals generated by the STDP tristate switch mechanism. Circles labelled with a particular value of *I* indicate the five possible filter states for the specific choice of $\varTheta = 3$; the two circles labelled $\pm \varTheta ^*$ in panel B represent absorbing states enabling FPT calculations. The transitions are labelled $\mathscr {I}^+$ and $\mathscr {I}^-$ to indicate potentiating and depressing induction signals, respectively. The additional labels $\Uparrow$ and $\Downarrow$ indicate transitions in which the expression of synaptic plasticity is triggered

The plasticity induction signals generated by the STDP switch may lead to the immediate expression of a change in synaptic strength of the corresponding synapse. However, because of the stochastic nature of our STDP model, fluctuations can destabilise developmentally-relevant patterns of synaptic connectivity (Appleby and Elliott 2006; Elliott 2008). These fluctuations can be suppressed by taking the plasticity step size *T* to be very, in fact implausibly small, which slows down the overall rate of synaptic plasticity and therefore allows synaptic self-averaging to occur (Appleby and Elliott 2006; Elliott 2008). Alternatively, we have proposed that synapses integrate synaptic plasticity induction signals before expressing synaptic plasticity. These integrate-and-express models of synaptic plasticity allow synapses to act as low-pass filters, suppressing high-frequency noise in plasticity induction signals while passing any low-frequency trends in them (Elliott 2008; Elliott and Lagogiannis 2009). Many such models are possible, but here we consider only the simplest. Any given synapse is postulated to instantiate in its molecular machinery the state of a discrete synaptic filter. We have discussed elsewhere how a synapse might do this (Elliott 2011a). A potentiating induction signal leads to an increment in filter state, while a depressing induction signal leads to a decrement. When the filter reaches an upper threshold, an increase in synaptic strength of *T* (or, more generally, $T_+$) is expressed, while if it reaches a lower threshold, a decrease of *T* (or $T_-$) is expressed. Upon reaching either threshold, the filter is then also reset to some other state, which we take to be the zero filter state. Filter states can then be represented by integers, with the upper threshold at some integer value $+ \varTheta _+ > 0$ and the lower threshold at some other integer value $- \varTheta _- < 0$, where $\varTheta _\pm > 0$. We will take the two thresholds to be symmetrical with respect to the zero filter state, so that $\varTheta _\pm = \varTheta$ for some common value $\varTheta$. Filter states are indexed by letters such as *I* and *J*, where $I,J \in \{ -(\varTheta - 1), \ldots , 0, \ldots + (\varTheta - 1) \}$. The filter state performs a one-step random walk on this discrete set with absorbing boundaries at $\pm \varTheta$. The one-step transitions are driven by the spike-induced UP $\rightarrow$ OFF and DOWN $\rightarrow$ OFF transitions from the STDP tristate switch, whose FPT densities we calculated above. Synaptic plasticity is expressed at the absorbing boundaries at $\pm \varTheta$, at which the filter is then reset to the zero state. Figure 2A illustrates the transitions of such a synaptic filter for the particular case of $\varTheta = 3$, explicitly showing the resetting of the filter to the zero state at threshold.

Because synaptic plasticity is expressed at either filter threshold, we have the familiar issue of the transition rates in Fig. 2A changing due to these very transitions even when $\lambda _\pi$ is fixed. In Fig. 2B, we therefore explicitly represent the thresholds as absorbing boundary states so that for fixed $\lambda _\pi$ and thus fixed $\lambda _p$, the transition rates are fixed. The temporal dynamics of the expression of synaptic plasticity are therefore determined by the FPT process for escape to these absorbing boundaries. To obtain the FPT densities for filter threshold events, we write down a renewal equation governing the transitions in joint synaptic STDP switch and filter states (see, e.g. Cox 1962, for a discussion of renewal equations). Let $F_{J, Y}^{I, X}(t)$ be the probability for the transition from switch state *Y* and filter state *J* to switch state *X* and filter state *I* in time *t* without either filter threshold having been reached. Then, we have the renewal equation

17 $$\begin{aligned}&F_{J, Y}^{I, X}(t) = \delta _J^I \, F_Y^X(t) \,\nonumber \\&\!+\! \int _0^t \!\textrm{d} \tau \, \Big [ F_{J + 1, \textrm{OFF}}^{I, X} (t\!-\!\tau ) G_Y^+(\tau ) + F_{J - 1, \textrm{OFF}}^{I, X} (t\!-\!\tau ) \, G_Y^-(\tau ) \Big ], \end{aligned}$$

for $| I | < \varTheta$ and $| J | < \varTheta$, subject to the boundary conditions $F_{\pm \varTheta ,Y}^{I, X}(t) = 0$.^2^ As convolution integrals typically appear in renewal equations, we will usually use the standard notation

$$\begin{aligned} (f *g) (t) = \int _0^t \textrm{d}\tau \, f(t - \tau ) g(\tau ) \end{aligned}$$

for brevity. The interpretation of Eq. (17) is straightforward. The inhomogeneous term on the right-hand side allows for the possibility that an induction signal is not generated by the STDP switch in time *t*. In this case, the filter state cannot change (hence $\delta _J^I$), with the switch state possibly changing but not via a spike-driven induction process (hence $F_Y^X(t)$). The homogeneous terms allow for the possibility that an induction signal is first generated at some time $0< \tau < t$. This may be a potentiating (hence $G_Y^+(\tau )$) or depressing (hence $G_Y^-(\tau )$) induction signal. The initial filter state *J* will then have increased to $J+1$ or decreased to $J-1$, respectively, but in either case, the STDP switch will be in the OFF state immediately after the induction signal has been generated at time $\tau$. In the remaining time $t-\tau$, the synapse must then transition from filter states $J \pm 1$ and the OFF switch state to the final states *I* and *X* (hence $F_{J {\pm } 1, \textrm{OFF}}^{I, X} (t-\tau )$). The imposed boundary conditions exclude the possibility that threshold is reached in either of these two induction cases, as by definition $F_{J, Y}^{I, X}(t)$ is the transition probability without threshold being reached. These three possibilities of no induction signal or a first induction signal of either potentiating or depressing type are mutually exclusive and exhaustive, so they just sum on the right-hand side.

Equation (17) integrates out the internal STDP switch transitions by focusing only on the induction densities rather than considering the detailed internal switch dynamics by which the induction signals are generated. It therefore integrates out the “microscopic” switch dynamics and works with the “mesoscopic” dynamics of filter transitions; the “macroscopic” dynamics discussed later will be the changes in strength triggered by filter threshold processes. To confirm that the non- or semi-Markovian mesoscopic renewal dynamics of Eq. (17) are completely equivalent to the underlying Markovian microscopic dynamics, we can differentiate Eq. (17) and use Eq. (7) for the induction densities $G_Y^\pm (t)$. After a little algebra we obtain

18 $$\begin{aligned} \frac{\textrm{d} F_{J, Y}^{I, X} (t)}{\textrm{d} t}&= \sum _Z \left( F_{J, Z}^{I, X}(t) \left[ \, \mathbb {S}_0 \right] _{Z,Y} \right) \nonumber \\&\quad + F_{J + 1, \textrm{OFF}}^{I, X} (t) \, v_Y^+ + F_{J - 1, \textrm{OFF}}^{I, X} (t) \, v_Y^- \nonumber \\&\quad + \delta _J^I \left[ \frac{\textrm{d} F_Y^X (t)}{\textrm{d} t} - \sum _Z \left( F_Z^X (t) \left[ \mathbb {S}_0 \right] _{Z,Y} \right) \right] , \end{aligned}$$

where the sums are over $Z \in \{ \textrm{DOWN}, [1] \textrm{OFF}, [1] \textrm{UP} \}$, and $v^\pm _Y$ are the components of $\underline{v}^\pm$. The expression multiplying $\delta _J^I$ on the right-hand side is just the relevant element of $\textrm{d} \mathbb {F}(t)/\textrm{d}t - \mathbb {F}(t) \, \mathbb {S}_0$. This vanishes identically because $\mathbb {F}(t)$ satisfies the forward equation $d \mathbb {F}(t)/\textrm{d}t = \mathbb {S}_0 \, \mathbb {F}(t)$, and hence, it also satisfies the backward equation $\textrm{d} \mathbb {F}(t)/\textrm{d}t = \mathbb {F}(t) \, \mathbb {S}_0$. The first term on the right-hand side is also just a matrix multiplication written out element-wise, again with post- rather than pre-multiplication by $\mathbb {S}_0$. This matrix gives transitions in STDP switch state without induction processes being generated, so the filter states do not change. The remaining two terms on the right-hand side account for the induction processes, in which the STDP switch returns to the OFF state and an increment or decrement in filter state occurs, with the imposed boundary conditions excluding the possibility of either filter threshold being reached. Equation (18) is just the backward form of the microscopic dynamics including all switch state transitions. We obtain the backward rather than forward form because the renewal equation in Eq. (17) can only be written in the backward form, in which the final states are fixed.

We can use the renewal equation in Eq. (17) to derive equations for the FPT densities $G_{J, Y}^{\pm \varTheta }$ for the escape through filter thresholds $\pm \varTheta$ from the initial filter state *J* and switch state *Y*. Using the equivalent relationship between $F_{J, Y}^{I, X} (t)$ and $G_{J, Y}^{\pm \varTheta } (t)$ as that between $F_Y^X (t)$ and $G_Y^\pm (t)$ in Eq. (9a), after taking the Laplace transform we obtain

19 $$\begin{aligned}&\widehat{G}_{J, Y}^{\, + \varTheta } (s) + \widehat{G}_{J, Y}^{\, - \varTheta } (s) \nonumber \\ &\quad = \big [ \widehat{G}_{J+1, \textrm{OFF}}^{\, +\varTheta } (s) + \widehat{G}_{J+1, \textrm{OFF}}^{\, -\varTheta }(s) \big ] \widehat{G}_Y^+(s) \nonumber \\&\qquad + \big [ \widehat{G}_{J-1, \textrm{OFF}}^{\, +\varTheta } (s) + \widehat{G}_{J-1, \textrm{OFF}}^{\, -\varTheta } (s) \big ] \widehat{G}_Y^+(s). \end{aligned}$$

Similar equations are satisfied by $\widehat{G}_{J, Y}^{\, \pm \varTheta } (s)$ individually, of which Eq. (19) is their sum. These equations are just

20 $$\begin{aligned} \widehat{G}_{J, Y}^{\, \pm \varTheta } (s) = \widehat{G}_{J+1, \textrm{OFF}}^{\, \pm \varTheta } (s) \, \widehat{G}_Y^+(s) + \widehat{G}_{J-1, \textrm{OFF}}^{\, \pm \varTheta } (s) \, \widehat{G}_Y^-(s), \end{aligned}$$

subject to the absorbing boundary conditions $\widehat{G}_{\pm \varTheta , \textrm{OFF}}^{\pm \varTheta } (s) = 1$ (same signs) and $\widehat{G}_{\mp \varTheta , \textrm{OFF}}^{\pm \varTheta }(s) = 0$ (opposite signs). It is clear that if we have solved for $\widehat{G}_{J, \textrm{OFF}}^{\pm \varTheta } (s)$, the density for escape starting from the OFF switch state, then the other densities $\widehat{G}_{J, Y}^{\pm \varTheta } (s)$ for $Y = \textrm{DOWN}$ and $Y = \textrm{UP}$ follow immediately. Setting $Y= \textrm{OFF}$ in Eq. (20), we obtain simple second-order recurrence relations, whose solutions are 21a21b where

22 $$\begin{aligned} \varPhi ^\pm (s) = \frac{1 \pm \sqrt{1 - 4 \, \widehat{G}_{\textrm{OFF}}^+(s) \, \widehat{G}_{\textrm{OFF}}^-(s)}}{2 \, \widehat{G}_{\textrm{OFF}}^+(s)}. \end{aligned}$$

Substituting the expressions in Eq. (21) into Eq. (20) at once gives all the $\widehat{G}_{J, Y}^{\pm \varTheta }(s)$. An equation very similar to Eq. (13) can then be used to obtain the Laplace transforms of the derivatives of the expression rates, $\widehat{\rho }_{J, Y}^{\pm \varTheta }(s)$. The expressions for $\widehat{\rho }_{0, \textrm{OFF}}^{\pm \varTheta }(s)$, namely

23a $$\begin{aligned} \widehat{\rho }_{0, \textrm{OFF}}^{+ \varTheta }(s)&= - s \, \frac{1}{\big ( \left[ \varPhi ^+(s) \right] ^\varTheta - 1 \big ) \big ( \left[ \varPhi ^-(s) \right] ^\varTheta - 1 \big )}, \end{aligned}$$

23b $$\begin{aligned} \widehat{\rho }_{0, \textrm{OFF}}^{- \varTheta }(s)&= - s \, \frac{\left[ \varPhi ^+(s) \varPhi ^-(s) \right] ^\varTheta }{\big ( \left[ \varPhi ^+(s) \right] ^\varTheta - 1 \big ) \big ( \left[ \varPhi ^-(s) \right] ^\varTheta - 1 \big )}, \end{aligned}$$

are particularly simple, and are of fundamental importance here, as we will see later.

We see from Eq. (17) that the various probabilities $P_{J, Y}^{\, I, X}(t)$ for $Y \in \{ \textrm{DOWN}, \textrm{OFF}, \textrm{UP} \}$ are controlled by the particular $Y = \textrm{OFF}$ case, so by the probability $P_{J, \textrm{OFF}}^{\, I, X} (t)$. This is also reflected in the escape densities $G_{J, Y}^{\pm \varTheta }(t)$, all of which are controlled by $G_{J, \textrm{OFF}}^{\pm \varTheta }(t)$. Starting from any initial switch state *Y*, one of the two possible initial induction densities $G_Y^\pm (t)$ will drive the first induction event. However, all subsequent induction events will then be driven by the densities $G_{\textrm{OFF}}^\pm (t)$, so starting from the OFF switch state. These OFF $\rightarrow$ OFF switch transitions that trigger induction events are the key processes that drive changes in synaptic filter state. This is the reason for the control of filter transition probabilities and the corresponding filter threshold densities by the particular $Y = \textrm{OFF}$ case. We have stressed the importance of these OFF $\rightarrow$ OFF switch transitions before (Elliott 2010).

Synaptic plasticity is expressed by a synapse when its filter reaches threshold, but we may again consider a consistent scheme in which expression does not occur. Provided that $\lambda _\pi$ and thus $\lambda _p$ are fixed, the rates of the transitions in Fig. 2A are then fixed. In this case, we can consider the probability $P_{J, Y}^{\, I, X}(t)$ for transitions between joint switch and filter states including the threshold processes and the subsequent resetting of the filter to the zero state ($F_{J, Y}^{I, X}(t)$ excludes the possibility of reaching threshold and thus of resetting). The renewal dynamics become

24 $$\begin{aligned}&P_{J, Y}^{\, I, X}(t) = F_Y^X(t) \, \delta _J^I \nonumber \\&\qquad + \big ( \big [ \big ( 1 - \delta _J^{\varTheta {-} 1} \big ) P_{J + 1, \textrm{OFF}}^{\, I, X} + \delta _J^{\varTheta - 1} \, P_{0, \textrm{OFF}}^{\, I, X} \big ] *G_Y^+ \big ) (t) \nonumber \\&\qquad + \big ( \big [ \big ( 1 - \delta _J^{1 - \varTheta } \big ) P_{J - 1, \textrm{OFF}}^{\, I, X} + \delta _J^{1 - \varTheta } \, P_{0, \textrm{OFF}}^{\, I, X} \big ] *G_Y^- \big ) (t). \end{aligned}$$

Boundary conditions are not required. Rather, the two factors $1 - \delta _J^{\pm (\varTheta - 1)}$ remove the upper and lower threshold process terms when $J = \pm (\varTheta - 1)$. They would lead to the incorrect terms $P_{\pm \varTheta , \textrm{OFF}}^{\, I, X}$ in this equation. Instead, these terms are replaced by the two terms multiplied by the two factors $\delta _J^{\pm (\varTheta - 1)}$. These terms correctly reset the filter state to zero at threshold, giving the two different $P_{0, \textrm{OFF}}^{\, I, X}$ contributions.

Unlike $F_{J, Y}^{I, X}(t)$, which vanish as $t \rightarrow \infty$ because filter threshold processes are inevitable given enough time, the transition probabilities $P_{J, Y}^{\, I, X}(t)$ do not in general vanish in the equilibrium limit. Taking the Laplace transform of Eq. (24), we can use the Tauberian theorem that $\lim _{s \rightarrow 0} s \, \widehat{P}_{J, Y}^{\, I, X} (s) = \lim _{t \rightarrow \infty } P_{J, Y}^{\, I, X} (t)$ to obtain

25 $$\begin{aligned}&\big [ r_Y^+ (\infty ) + r_Y^- (\infty ) \big ] P_{J, Y}^{\, I, X} (\infty ) \nonumber \\&\quad = r_Y^+ (\infty ) \Big [ \big ( 1 - \delta _J^{\varTheta - 1} \big ) P_{J + 1,\textrm{OFF}}^{\, I, X} (\infty ) + \delta _J^{\varTheta - 1} \, P_{0, \textrm{OFF}}^{\, I, X} (\infty ) \Big ] \nonumber \\&\qquad + r_Y^- (\infty ) \Big [ \big ( 1 - \delta _J^{1 - \varTheta } \big ) P_{J - 1,\textrm{OFF}}^{\, I, X} (\infty ) + \delta _J^{1 - \varTheta } \, P_{0, \textrm{OFF}}^{\, I, X} (\infty ) \Big ], \end{aligned}$$

where we have used the fact that

26 $$\begin{aligned} \lim _{s \rightarrow 0} \widehat{G}_Y^\pm (s) = \frac{r_Y^\pm (\infty )}{r_Y^+ (\infty ) + r_Y^- (\infty )}. \end{aligned}$$

Taking as usual $Y = \textrm{OFF}$ and defining the $(2\varTheta -1) \times (2\varTheta -1)$ matrix $\mathbb {E}$ with elements

27 $$\begin{aligned} {[} \, \mathbb {E} \, ]_{I,J}&= - \big [ r_{\textrm{OFF}}^+ (\infty ) + r_{\textrm{OFF}}^- (\infty ) \big ] \delta _{\, I, J} \nonumber \\&\quad + r_{\textrm{OFF}}^+ (\infty ) \big [ \delta _{\, I, J+1} + \delta _{\, I, 0} \, \delta _{\, \varTheta - 1, J} \big ] \nonumber \\&\quad + r_{\textrm{OFF}}^- (\infty ) \big [ \delta _{\, I, J-1} + \delta _{\, I, 0} \, \delta _{\, 1 - \varTheta , J} \big ], \end{aligned}$$

where we index the elements according to the corresponding filter state, Eq. (25) can be written in the form

28 $$\begin{aligned} \sum _{K=-(\varTheta {-}1)}^{+(\varTheta {-}1)} P_{K, \textrm{OFF}}^{\, I, X} (\infty ) \, [ \, \mathbb {E} \, ]_{K,J} = 0. \end{aligned}$$

For any given final switch state *X*, this equation just expresses an element-wise matrix multiplication involving a matrix of asymptotic transition probabilities for filter states and the matrix $\mathbb {E}$. We again have post-multiplication by the generating matrix $\mathbb {E}$ because of the backward form of renewal equations. In this backward form, Eq. (28) encodes the trivial fact that the rows of the asymptotic filter state transition matrix (for fixed final switch state *X*) are proportional to the null left eigenvector of $\mathbb {E}$, or just $\underline{1}^{\textrm{T}}$. For the columns, we must consider the forward form of Eq. (28), which just entails pre-multiplication by $\mathbb {E}$. Hence, all columns are the same (because of the row proportionality), with any given column proportional to the null right eigenvector of $\mathbb {E}$. Write this as $\underline{\varepsilon }$, where $\underline{\varepsilon } \cdot \underline{1} = 1$, so that $\underline{\varepsilon }$ is normalised to a probability distribution, and write its components as $\varepsilon _I$, again indexed by filter state. In any given equilibrium filter state *I* with probability $\varepsilon _I$, the system can reside in any of the three switch states $X \in \{ \textrm{DOWN}, \textrm{OFF}, \textrm{UP} \}$. Their equilibrium probability distribution is just $\sigma _X$. So, we must have $P_{J, \textrm{OFF}}^{\, I, X} (\infty ) = \varepsilon _I \, \sigma _X$. This equilibrium result must be independent of the particular initial choice of $Y = \textrm{OFF}$, which can be confirmed directly from Eq. (28). Hence, we have

29 $$\begin{aligned} P_{J, Y}^{\, I, X} (\infty ) = \varepsilon _I \, \sigma _X, \end{aligned}$$

as the asymptotic transition probabilities for joint filter and switch states.

An explicit calculation of the components $\varepsilon _I$ gives

30 $$\begin{aligned} \varepsilon _I = \left\{ \begin{array}{ll} {\displaystyle \frac{1}{\varTheta } \, \frac{1 - [ r_{\textrm{OFF}}^-(\infty ) / r_{\textrm{OFF}}^+(\infty ) ]^{\varTheta - I}}{1 - [ r_{\textrm{OFF}}^-(\infty ) / r_{\textrm{OFF}}^+(\infty ) ]^\varTheta }} & \text{ for } I \ge 0 \\ & \\ {\displaystyle \frac{1}{\varTheta } \, \frac{1 - [ r_{\textrm{OFF}}^+(\infty ) / r_{\textrm{OFF}}^-(\infty ) ]^{\varTheta + I}}{1 - [ r_{\textrm{OFF}}^+(\infty ) / r_{\textrm{OFF}}^-(\infty ) ]^\varTheta }} & \text{ for } I \le 0 \end{array} \right. , \end{aligned}$$

which may be verified by checking that $\mathbb {E} \, \underline{\varepsilon } = \underline{0}$. In equilibrium, the conditional rate of the expression of a potentiating step, conditional on the switch state being in the OFF state, is just probability of the filter being in state $I = +(\varTheta - 1)$ at equilibrium, $\varepsilon _{\varTheta -1}$, multiplied by the asymptotic rate of an induction step starting from the OFF switch state, $r_{\textrm{OFF}}^+(\infty )$. Similarly for the expression of a depressing step. Hence, these conditional equilibrium rates are just $r_{\textrm{OFF}}^\pm (\infty ) \, \varepsilon _{\pm (\varTheta - 1)}$ (same signs). They agree exactly with the asymptotic rates $r_{0, \textrm{OFF}}^{\pm \varTheta } (\infty ) = \widehat{\rho }_{0, \textrm{OFF}}^{\pm \varTheta } (0)$ obtained from the FPT densities for filter thresholds being reached starting from the OFF switch state and the zero filter state given in Eq. (23). We have

31 $$\begin{aligned} r_{0, \textrm{OFF}}^{\pm \varTheta } (\infty ) = \frac{\left[ r_{\textrm{OFF}}^\pm (\infty )\right] ^\varTheta }{\varTheta } \, \frac{\left[ r_{\textrm{OFF}}^+(\infty )\right] - \left[ r_{\textrm{OFF}}^-(\infty )\right] }{\left[ r_{\textrm{OFF}}^+(\infty )\right] ^\varTheta - \left[ r_{\textrm{OFF}}^-(\infty )\right] ^\varTheta }. \end{aligned}$$

We note again that although the asymptotic rates $r_{0, \textrm{OFF}}^{\pm \varTheta } (\infty )$ explicitly depend on starting from the zero filter state, the equilibrium rates $r_{\textrm{OFF}}^\pm (\infty ) \, \varepsilon _{\pm (\varTheta - 1)}$ have by definition forgotten about the initial filter state.

### Expressing changes in synaptic strength

We are now in a position to write down a renewal equation for joint changes in synaptic strength, filter and switch states. A synapse’s strength is assumed to take only discrete values, with a uniform spacing of *T* between consecutive strengths. We refer to a synapse’s strength state rather than its strength, indexing the strength state by letters such as *A* and *B*. As we consider only excitatory synapses, strengths cannot be negative, so $A,B \in \{ 0, 1, 2, \ldots \}$. The strength of a synapse in strength state *B* is just *TB*. It will be convenient to employ an upper bound on synaptic strengths, but only for the purposes of obtaining numerical solutions of equations rather than as a hard bound to prevent run-away excitation. Analytically this upper bound is not required and we can often ignore it. When required, we take $A,B \in \{ 0, 1, \ldots , N-1, N \}$, with *N* denoting the highest strength state available. We will always take *N* large enough, when required, so that it does not have a significant impact on our results. For a postsynaptic cell with just one synaptic input, as we are currently considering, its spike rate $\lambda _p$ is a function of the presynaptic spike rate $\lambda _\pi$ and the strength state *B* of its synaptic input. We set

32 $$\begin{aligned} \lambda _p = \left\{ \begin{array}{ll} \lambda _{\textrm{spont}} & \text{ for } B=0 \\ \lambda _\pi T B & \text{ for } B>0 \end{array} \right. , \end{aligned}$$

where $\lambda _{\textrm{spont}}$ is a spontaneous postsynaptic spike firing rate whose presence is essential to ensure the existence of non-trivial equilibrium patterns of synaptic connectivity (Elliott 2011b). The switch and filter state transitions considered above depend on both the presynaptic spike firing rate and the postsynaptic spike firing rate. They therefore depend explicitly on the synaptic strength state via $\lambda _p$. If a synapse is in strength state *B*, we denote this dependence by writing, for example, the FPT densities as $G_{J, Y; \, B}^{\pm \varTheta } (t)$ and $G_{J; \,B}^\pm (t)$ rather than the earlier $G_{J, Y}^{\pm \varTheta } (t)$ and $G_J^\pm (t)$, and other related quantities are similarly modified.

The renewal equation for the transition probability from joint synaptic strength state *B*, filter state *J* and switch state *Y* to joint states *A*, *I* and *X* in time *t* is, for $0<B<N$,

33 $$\begin{aligned} P_{B, J, Y}^{\, A, I, X}(t)&= \delta _B^A \, F_{J, Y; \, B}^{\, I, X}(t) \nonumber \\&\quad + \big ( P_{B + 1, 0, \textrm{OFF}}^{\, A, I, X} *G_{J, Y; \, B}^{+ \varTheta } \big ) (t) \nonumber \\ &\quad + \big ( P_{B - 1, 0, \textrm{OFF}}^{\, A, I, X} *G_{J, Y; \,B}^{- \varTheta } \big ) (t). \end{aligned}$$

For $B=0$, we replace the depressed term $P_{B - 1, 0, \textrm{OFF}}^{\, A, I, X}$ on the right-hand side with $P_{0, 0, \textrm{OFF}}^{\, A, I, X}$ to enforce a reflecting boundary condition at the lowest strength state, while with an upper boundary at $B=N$, we replace the potentiated term $P_{B+1, 0, \textrm{OFF}}^{\, A, I, X}$ with $P_{N, 0, \textrm{OFF}}^{\, A, I, X}$. These reflecting boundaries enforce saturation dynamics in synaptic strength. Notice that despite this saturation, the switch and filter states are still returned to the OFF and zero states, respectively. We assume that the dynamics of switch and filter state transitions are not explicitly sensitive to a synapse’s current strength state (they are implicitly sensitive, but only via the dependence of $\lambda _p$ on synaptic strength). Thus, a threshold filter transition caused by an induction signal triggered by the STDP switch will lead to an expression signal regardless of the synapse’s current strength state. The filter and STDP switch will then reset. Whether or not the synapse can actually act on the expression signal and implement a change in its strength state should not affect the filter and switch processes.

For a single synapse, Eq. (33) is exact, with no approximations having been made to obtain it. It integrates out the details of the underlying filter and switch processes by considering only the filter threshold densities. We may again confirm by differentiation that the macroscopic strength dynamics of Eq. (33) correctly describe the mesoscopic filter and microscopic switch dynamics that generate the expression and induction signals leading to macroscopic strength changes. The equation essentially describes a one-step random walk on a finite or semi-infinite set of strength states between reflecting boundaries, where the expression densities drive the steps. Although the detailed sub-macroscopic dynamics have been integrated out, Eq. (33) retains vestiges of them by referencing the initial filter and switch states *J* and *Y*, respectively. It is clear, however, that the fundamental process is driven by the transitions starting from the OFF switch state and the zero filter state. We have already discussed the significance of the OFF $\rightarrow$ OFF switch transitions. The zero $\rightarrow$ zero filter state transitions are also fundamental in the same way. This is reflected in the presence of the terms $P_{B \pm 1, 0, \textrm{OFF}}^{\, A, I, X}(t)$ in Eq. (33). Once the first expression step has occurred, the system has forgotten its initial filter and switch states, and the key dynamics are then driven by the expression processes from the joint zero filter and OFF switch states back to these states.

If we were to solve Eq. (33), we would first set $J = 0$ and $Y = \textrm{OFF}$ to obtain a recurrence relation in *B* for $P_{B, 0, \textrm{OFF}}^{\, A, I, X}(t)$, and then the general forms $P_{B, J, Y}^{\, A, I, X}(t)$ would follow. The key transition probabilities of interest are therefore $P_{B, 0, \textrm{OFF}}^{\, A, I, X}(t)$, which control all the others. Furthermore, we will be concerned with the long-term, equilibrium behaviour of $P_{B, 0, \textrm{OFF}}^{\, A, I, X}(t)$ (or some suitable version thereof), and this must become independent of the initial joint state. Therefore, we may without loss of generality simply set $J = 0$ and $Y = \textrm{OFF}$ in Eq. (33). Since we are interested only in the macroscopic dynamics of the evolution of synaptic strength states, we can also just sum over the final switch state *X* and filter state *I*, to obtain the marginal transition probability to strength state *A* from strength state *B*. By writing $\mathscr {P}_B^{\, A}(t) = \sum _{I, X} P_{B, 0, \textrm{OFF}}^{\, A, I, X} (t)$, using the relationship between $\sum _{I, X} F_{J, Y; \, B}^{I, X}(t)$ and $G_{J, Y; \, B}^{\pm \varTheta }(t)$, taking the Laplace transform and then expressing $\widehat{G}_{J, Y; \, B}^{\pm \varTheta } (s)$ in terms of $\widehat{\rho }_{J, Y; \, B}^{\pm \varTheta }(t)$, Eq. (33) becomes

34 $$\begin{aligned} s \, \widehat{\mathscr {P}}_B^{\, A} (s) - \delta _B^A&= \big [ \widehat{\mathscr {P}}_{B+1}^{\, A} (s) - \widehat{\mathscr {P}}_B^{\, A} (s) \big ] \, \widehat{\rho }_{0,\textrm{OFF}; \, B}^{+ \varTheta } (s) \nonumber \\&\quad + \big [ \widehat{\mathscr {P}}_{B-1}^{\, A} (s) - \widehat{\mathscr {P}}_B^{\, A} (s) \big ] \, \widehat{\rho }_{0, \textrm{OFF}; \, B}^{- \varTheta } (s), \end{aligned}$$

with obvious modifications at the reflecting boundaries. Since $\mathscr {P}_B^{\, A} (0) = \delta _B^A$, we recognise the left-hand side as the Laplace transform of $\textrm{d} \mathscr {P}_B^{\, A}(t)/\textrm{d}t$. Undoing the Laplace transform, we have

35 $$\begin{aligned} \frac{\textrm{d} \mathscr {P}_B^{\, A} (t)}{\textrm{d} t}&= \big ( \big [ \mathscr {P}_{B + 1}^{\, A} - \mathscr {P}_B^{\, A} \, \big ] *\rho _{0, \textrm{OFF}; \, B}^{+ \varTheta } \big ) (t) \nonumber \\&\quad + \big ( \big [ \mathscr {P}_{B - 1}^{\, A} - \mathscr {P}_B^{\, A} \, \big ] *\rho _{0, \textrm{OFF}; \,B}^{- \varTheta } \big ) (t). \end{aligned}$$

This is a backward equation for the evolution of the strength state transition probabilities $\mathscr {P}_B^{\, A}(t)$ involving the memory kernels $\rho _{0, \textrm{OFF}; \, B}^{\pm \varTheta } (t)$.

Since we have a backward equation, it is extremely tempting to move immediately to its forward form, which normally involves the trivial step of pre- rather than post-multiplying by the transition matrix implied by Eq. (35). In a backward equation, the final (joint) state is fixed and we evolve backwards in time to the probability distribution of the initial (joint) state. In a forward equation, the initial (joint) state is fixed and we evolve forwards in time to the probability distribution of the final (joint) state. Critically, however, in obtaining Eq. (35), we have summed over the final switch state *X* and filter state *I* and set their initial states to $J = 0$ and $Y = \textrm{OFF}$, since $\mathscr {P}_B^{\, A} (t) = \sum _{I, X} P_{B, 0, \textrm{OFF}}^{\, A, I, X} (t)$. Although moving between the forward and backward Chapman–Kolmogorov equations for the evolution of the full transition probability $P_{B, J, Y}^{\, A, I, X} (t)$ does involve the trivial transposition of the transition operator, no such move is therefore possible for the marginalised and restricted transition probability $\mathscr {P}_B^{\, A} (t)$.

Nevertheless, Eq. (35) highlights the centrality of the internal state transitions in which, starting from the OFF switch and zero filter states, a synapse first expresses a change in strength state via an upper or lower filter threshold process, returning immediately back to the OFF switch and zero filter states. The memory kernels $\rho _{0, \textrm{OFF}; \, B}^{\pm \varTheta } (t)$ integrate out all the internal details by which these two OFF & zero $\rightarrow$ OFF & zero strength-changing transitions occur. As we are only interested in these internal dynamics insofar as they drive changes in strength state, we can elevate the memory kernels $\rho _{0, \textrm{OFF}; \, B}^{\pm \varTheta } (t)$ to a fundamental status. We can therefore consider them as defining a one-step non- or semi-Markovian random walk on the set of strength states, inducing the transition probability $P_B^{\, A} (t)$ between these strength states, where these strength states are now considered as structureless and irreducible. The backward equation describing this random walk is then of course identical to Eq. (35):

36 $$\begin{aligned} \frac{\textrm{d} P_B^{\, A} (t)}{\textrm{d} t}&= \big ( \big [ P_{B + 1}^{\, A} - P_B^{\, A} \, \big ] *\rho _{0, \textrm{OFF}; \, B}^{+ \varTheta } \big ) (t) \nonumber \\&\quad + \big ( \big [ P_{B - 1}^{\, A} - P_B^{\, A} \, \big ] *\rho _{0, \textrm{OFF}; \,B}^{- \varTheta } \big ) (t). \end{aligned}$$

Clearly, the evolution of $P_B^{\, A} (t)$ and $\mathscr {P}_B^{\, A} (t)$ are identical, but this identity is established not by equivalence but by formal correspondence. However, unlike $\mathscr {P}_B^{\, A} (t)$, for which we cannot move immediately to the forward equation, the move to the forward Chapman–Kolmogorov equation for $P_B^{\, A} (t)$ is now indeed just a trivial matter of matrix transposition. Although there is a formal correspondence between $P_B^{\, A} (t)$ and $\mathscr {P}_B^{\, A} (t)$, this difference emphasises the deep conceptual gulf between these transition probabilities: $P_B^{\, A} (t)$ knows nothing about the internal states, while $\mathscr {P}_B^{\, A} (t) = \sum _{I, X} P_{B, 0, \textrm{OFF}}^{\, A, I, X} (t)$ has simply marginalised over the final internal states and restricted to specific initial internal states.

The forward equation for $P_B^{\, A} (t)$ is just

37 $$\begin{aligned}&\frac{\textrm{d} P_B^{\, A}(t)}{\textrm{d} t} = \big ( \rho _{0, \textrm{OFF}; \, A - 1}^{+ \varTheta } *P_B^{\, A - 1} \big ) (t) - \big ( \rho _{0, \textrm{OFF}; \,A}^{+ \varTheta } *P_B^{\, A} \big ) (t) \nonumber \\&\quad + \big ( \rho _{0, \textrm{OFF}; \, A + 1}^{- \varTheta } *P_B^{\, A + 1} \big ) (t) - \big ( \rho _{0, \textrm{OFF}; \,A}^{- \varTheta } *P_B^{\, A} \big ) (t). \end{aligned}$$

We have re-ordered the products in Eq. (37) compared to Eq. (36) because, in a forward equation, the expression step is the last rather than the first step. Equation (37) is the general form for $0< A < N$. For $A = 0$, we replace $\rho _{0, \textrm{OFF}; \, A - 1}^{+ \varTheta } *P_B^{\, A-1}$ by $\rho _{0, \textrm{OFF}; \, 0}^{- \varTheta } *P_B^{\, 0}$, which replaces a potentiating step from the non-existent $A = -1$ state with the reflected depressing step from the $A=0$ state itself. There is of course then a cancellation of the two $A=0$ depression terms in Eq. (37), but explicitly including the reflection process is conceptually clearer. If we also have an upper, reflecting boundary at $A = N$, then we would replace $\rho _{0, \textrm{OFF}; \, A + 1}^{- \varTheta } *P_B^{\, A + 1}$ by $\rho _{0, \textrm{OFF}; \, N}^{+ \varTheta } *P_B^{\, N}$, which now replaces a depressing step from the non-existent $A=N+1$ state with the reflected potentiating step from the $A=N$ state itself.

The forward equation in Eq. (37) for the transition probabilities $P_B^{\, A}(t)$ is of course just equivalent to the master equation for the evolution of the strength states themselves, starting from the definite initial strength state *B*. We write the probability of strength state *A* as $P_{A; \, 0, \textrm{OFF}} (t)$, where we add the zero and OFF labels to remind ourselves that these are state probabilities driven exclusively by the two OFF & zero $\rightarrow$ OFF & zero strength-changing transitions. We stress that these are merely labels, and do not indicate internal states. This distinction will be vital in Sect. 3. The master equation is just

38 $$\begin{aligned}&\frac{\textrm{d} P_{A; \, 0, \textrm{OFF}}(t)}{\textrm{d} t} \nonumber \\&\quad = \big ( \rho _{0, \textrm{OFF}; \, A - 1}^{+ \varTheta } *P_{A - 1; \, 0, \textrm{OFF}} \big ) (t) \nonumber \\&\qquad - \big (\rho _{0, \textrm{OFF}; \,A}^{+ \varTheta } *P_{A; \, 0, \textrm{OFF}} \big ) (t) \nonumber \\&\qquad + \big ( \rho _{0, \textrm{OFF}; \, A + 1}^{- \varTheta } *P_{A + 1; \, 0, \textrm{OFF}} \big ) (t) \nonumber \\&\qquad - \big ( \rho _{0, \textrm{OFF}; \,A}^{- \varTheta } *P_{A; \, 0, \textrm{OFF}} \big ) (t), \end{aligned}$$

again with the same boundary modifications. We can take the Laplace transform of Eq. (38) to remove the convolution structure and solve directly for $\widehat{P}_{A; \, 0, \textrm{OFF}}(s)$, obtaining $P_{A; \, 0, \textrm{OFF}}(t)$ for any finite time *t*, at least in principle even when $\lambda _\pi$ is time-dependent. Here we are mainly concerned with the equilibrium structure of the probability distribution of synaptic strength states rather than the process of evolution towards this equilibrium distribution. This focus is equivalent to considering the outcome (or the multiple possible outcomes) of neuronal development rather than the temporal dynamics of neuronal development. For the unrealistic case of fixed $\lambda _\pi$, $P_{A; \, 0, \textrm{OFF}}(t)$ has a well-defined equilibrium limit. However, for the realistic case of time-dependent $\lambda _\pi$, an equilibrium limit in the conventional sense does not in general exist. This limit would have to be defined by considering the ensemble of all possible patterns of $\lambda _\pi (t)$, taking the ensemble average $\langle P_{A; \, 0, \textrm{OFF}}(t) \rangle _{\lambda _\pi }$ over these patterns, and finally taking the limit $t \rightarrow \infty$. Whether this calculation is feasible remains to be seen. We must instead resort to approximation methods to separate time scales, which we discuss next.

### Separation of time scales

For the first approximation, we consider the behaviour of the rates $r_{\textrm{OFF}; \, B}^\pm (t)$ of induction signals generated by the STDP switch mechanism, starting from the OFF state. Assuming that $\lambda _\pi$ is fixed and with $\lambda _p$ therefore also fixed for processes not involving a change in synaptic strength state *B*, the asymptotic behaviour of the induction rates is in fact achieved very quickly. For the typical presynaptic spike rate $\lambda _\pi$ that we consider below and with $\lambda _- = 50$ Hz (or $\tau _- = 20$ ms) and $\lambda _+ \approx 83$ Hz (or $\tau _+ = 12$ ms), asymptotic behaviour is achieved within around 10 ms (Elliott 2011b). This time scale is much shorter than the time scale on which natural stimulus patterns change in the visual system, for example. This latter time scale is determined by the average fixation time between saccades, and so is perhaps of order at least 100 ms. Hence, using a fixed spike firing rate $\lambda _\pi$ to determine the time scale on which the induction rates attain their asymptotic behaviours is a consistent procedure, because the spike firing rate is indeed essentially fixed during the much longer fixation periods. In addition, as 10 ms is much shorter than typical stimulus times and certainly much shorter than any relevant time scales involving synaptic plasticity (see, e.g. Elliott 2011a), to a good approximation we may regard $r_{\textrm{OFF}; \, B}^\pm (t)$ as behaving like a scaled Heaviside step function, instantaneously jumping from 0 Hz at $t=0$ s to its asymptotic rate for $t > 0$ s. Thus, we write $r_{\textrm{OFF}; \, B}^\pm (t) \approx r_{\textrm{OFF}; \, B}^\pm (\infty ) \, H(t)$, where for concreteness we define $H(t) = 0$ for $t \le 0$ s and $H(t) = 1$ for $t > 0$ s. We then also have $\rho _{\textrm{OFF}; \, B}^\pm (t) = \textrm{d} r_{\textrm{OFF}; \, B}^\pm (t)/\textrm{d}t \approx r_{\textrm{OFF}; \, B}^\pm (\infty ) \, \delta (t)$. We saw earlier that the asymptotic FPT induction rates $r_{\textrm{OFF}; \, B}^\pm (\infty )$ match exactly the induction rates $r_B^+ = \lambda _p \, \sigma _{\, \textrm{UP}}$ and $r_B^- = \lambda _\pi \, \sigma _{\, \textrm{DOWN}}$ obtained from the equilibrium distribution $\underline{\sigma }$ of switch states. We therefore simplify the notation by stripping off the subscript referring to the OFF switch state in these asymptotic induction rates, and instead just write $r_B^\pm (t) \approx r_B^\pm \, H(t)$ and $\rho _B^\pm (t) \approx r_B^\pm \, \delta (t)$. The Laplace transforms of the switch FPT densities for $Y = \textrm{OFF}$ in Eq. (14) then become the simple expressions (again stripping off the subscript OFF)

39 $$\begin{aligned} \widehat{G}_B^\pm (s) = \frac{r_B^\pm }{s + r_B^+ + r_B^-}, \end{aligned}$$

so that $G_B^\pm (t) = r_B^\pm \, \exp [ - (r_B^+ + r_B^-) \, t ]$, which are just exponentially distributed densities. An explicit calculation of the asymptotic or equilibrium rates $r_B^\pm$ gives

40a $$\begin{aligned} r_B^+&= \frac{\lambda _\pi \lambda _p (\lambda _\pi + \lambda _-)}{(\lambda _\pi + \lambda _p + \lambda _+) (\lambda _\pi + \lambda _p + \lambda _-) - \lambda _\pi \lambda _p}, \end{aligned}$$

40b $$\begin{aligned} r_B^-&= \frac{\lambda _\pi \lambda _p (\lambda _p + \lambda _+)}{(\lambda _\pi + \lambda _p + \lambda _+) (\lambda _\pi + \lambda _p + \lambda _-) - \lambda _\pi \lambda _p}, \end{aligned}$$

which are just Eqs. (2.5) and (2.6) in Elliott (2011b) with $s \rightarrow 0$ (or in the notation there, $p \rightarrow 0$).

For the second approximation, we consider the expression rates $r_{0, \textrm{OFF}; \, B}^{\pm \varTheta } (t)$ rather than the induction rates $r_{\textrm{OFF}; \, B}^\pm (t)$. As we now strip off the subscript OFF on $r_{\textrm{OFF}; \, B}^\pm (t)$, we do the same on $r_{0, \textrm{OFF}; \, B}^{\pm \varTheta } (t)$ and related functions, but it will be essential to retain reference to the initial filter state in Sect. 3, so we do not also strip off the subscript 0. Equation (21) gives the Laplace transforms of the expression densities $\widehat{G}_{0; \, B}^{\pm \varTheta } (s)$ (setting $J = 0$), where in the expressions for $\varPhi ^\pm (s)$ we use the approximation in Eq. (39) for the Laplace transformed induction densities. Because the Laplace transform of a probability density function is, up to the sign of *s*, just the moment generating function (MGF) of the distribution, we can use $\widehat{G}_{0; \, B}^{\pm \varTheta } (-s)$ to obtain the mean time to express any change in synaptic strength state. Direct calculation shows this to be

41 $$\begin{aligned} \langle \tau _{\textrm{exp}} \rangle = \frac{\varTheta }{( r_B^+ )^\varTheta + ( r_B^- )^\varTheta } \, \frac{( r_B^+ )^\varTheta - ( r_B^- )^\varTheta }{r_B^+ - r_B^-}. \end{aligned}$$

For $\varTheta = 1$, this time is well under one second for our typical choice of $\lambda _\pi$ and with $\lambda _p$ determined by direct synaptic drive (with $\lambda _- = 50$ Hz and $\lambda _+ \approx 83$ Hz). However, as $\varTheta$ increases, the mean expression time increases. For $\varTheta = 5$, it is typically a few seconds; for $\varTheta = 10$, around ten seconds; for $\varTheta = 20$, it approaches one minute; for $\varTheta = 40$, a few minutes; and for $\varTheta = 80$, closer to ten minutes. Choices of $\varTheta$ that are too large are unrealistic from a biological point of view (Elliott 2011a), with $\varTheta = 80$ likely too large but $\varTheta = 40$ acceptable. For reasonable choices of $\varTheta$ then, neither too small nor too large, filter threshold processes occur on time scales ranging from around ten seconds up to around several minutes. These time scales are much longer than typical stimulus times driven by fixation periods of perhaps around 100 ms or somewhat more. Thus, in Eqs. (17) and (24) (with $Y = \textrm{OFF}$) for the filter dynamics, the induction rates $r_B^\pm$ appearing via Eq. (39) cannot be regarded as fixed because the stimuli change on a time scale at least at an order of magnitude, perhaps even two orders of magnitude faster than any filter threshold process. However, we may exploit the fact that the time scales are so different to make a second approximation. We assume that the filter dynamics in Eqs. (17) and (24) sample the changing induction rates $r_B^\pm$ sufficiently often that we can replace the time-dependent rates $r_B^\pm$ with their fixed ensemble averages $\langle r_B^\pm \rangle$, where the average is taken over all possible stimulus patterns. This is an adiabatic approximation that allows a separation of processes occurring on two rather different time scales (Elliott 2011b).

To average over the stimulus patterns of *m* afferents with spike rates $\lambda _i$, $i = 1, \ldots , m$, we use our earlier methods (Elliott 2011b). We write $\lambda _i = \mu + \alpha _i$ and define the first- and second-order statistics of the fluctuations $\alpha _i$ by

42a $$\begin{aligned} \langle \alpha _i \rangle&= 0, \end{aligned}$$

42b $$\begin{aligned} \langle \alpha _i \, \alpha _j \rangle&= \varsigma ^2 \, \mathfrak {r}_{i j}, \end{aligned}$$

so that $\mu$ is the common mean spike firing rate, $\varsigma ^2$ is the common variance in each afferent’s spike firing rate, and $\mathfrak {r}_{i j}$ is the correlation coefficient between any pair of afferents’ spike firing rates; for $i \ne j$, we take a common correlation coefficient $\mathfrak {r}$, although we usually just set it to zero, i.e. $\mathfrak {r}_{i j} = \delta _{i j}$. In simulation, we take $\lambda _i \in \{ \mu - \varsigma , \mu + \varsigma \}$ with equal probability, with suitable pairwise correlations where necessary. Provided that $\mu / \varsigma$ is sufficiently small, we showed that this restricted set of simulated spike firing rates fully captures an analytical ensemble average over any spike firing rate distribution satisfying the statistics in Eq. (42) (Elliott 2011b). Simulations are defined by firing epochs of duration 1000 ms. Spike firing rates are constant within each epoch and change between them. We take $\mu = 50$ Hz and allow a standard deviation $\varsigma$ of 25% around this mean value, in accordance with our previous protocols (Elliott 2011b).

The asymptotic and adiabatic approximations simplify the filter dynamics. For example, setting $Y = \textrm{OFF}$ and summing over $X \in \{ \textrm{DOWN}, \textrm{OFF}, \textrm{UP} \}$ in Eq. (17) to obtain the transition probability between filter states without threshold events, $F_{J; \, B}^I(t)$ (again stripping off the OFF subscript and adding the strength state *B*), we obtain

43 $$\begin{aligned} \frac{\textrm{d} F_{J; \, B}^I (t)}{\textrm{d} t}&= \big [ F_{J + 1; \, B}^I (t) - F_{J; \, B}^I (t) \big ] \, \langle r_B^+ \rangle \nonumber \\&\quad + \big [ F_{J - 1; \, B}^I (t) - F_{J; \, B}^I (t) \big ] \, \langle r_B^- \rangle , \end{aligned}$$

again with boundary conditions $F_{\pm \varTheta ; \, B}^I (t) = 0$. We have obtained this by employing the standard trick of taking the Laplace transform, using Eq. (39), and then undoing the transform. What would have been the memory kernels $\rho _B^\pm (t)$ appearing under convolution integrals on the right-hand side of Eq. (43) have collapsed the integrals down because with the asymptotic approximation, we have $\rho _B^\pm (t) \approx r_B^\pm \, \delta (t)$. Then, with the adiabatic approximation, $r_B^\pm$ are replaced by their averages $\langle r_B^\pm \rangle$. A similar simplification of Eq. (24) also occurs. Equation (43) can be solved explicitly. Writing it as the matrix equation $\textrm{d} \mathbb {F}_B(t) / \textrm{d}t = \mathbb {F}_B(t) \, \mathbb {E}_0$, the matrix elements $[ \, \mathbb {E}_0 ]_{I,J}$ are

44 $$\begin{aligned} {[} \, \mathbb {E}_0 ]_{I,J} = - \big [ \langle r_B^+ \rangle + \langle r_B^- \rangle \big ] \, \delta _{\, I, J} + \langle r_B^+ \rangle \, \delta _{\, I, J+1} + \langle r_B^- \rangle \, \delta _{\, I, J-1}. \end{aligned}$$

The matrix $\mathbb {E}_0$ is just the earlier matrix $\mathbb {E}$, whose elements are given in Eq. (27), but lacking the filter resetting terms and having been ensemble averaged. Because $\mathbb {E}_0$ is a tridiagonal matrix, its eigen-decomposition is standard, and so the filter transition probabilities without threshold processes can be explicitly computed (van Kampen, 1992; see also Elliott and Lagogiannis, 2012). We obtain

45 $$\begin{aligned} F_{J; \, B}^I (t)&= \frac{1}{\varTheta } \sum _{K=-(\varTheta -1)}^{+(\varTheta -1)} \left[ \left( {\textstyle \frac{\langle r_B^+ \rangle }{\langle r_B^- \rangle }} \right) ^{(\varTheta + I)/2} \sin {\textstyle \frac{(\varTheta + I)(\varTheta + K) \pi }{2 \varTheta } } \right] \nonumber \\&\quad \times \left[ \left( {\textstyle \frac{\langle r_B^- \rangle }{\langle r_B^+ \rangle }} \right) ^{(\varTheta + J)/2} \sin {\textstyle \frac{(\varTheta + J)(\varTheta + K) \pi }{2 \varTheta }} \right] \nonumber \\&\quad \times \exp \left\{ \left[ - \langle r_B^+ \rangle - \langle r_B^- \rangle + 2 \sqrt{\langle r_B^+ \rangle \langle r_B^- \rangle } \, \cos {\textstyle \frac{(\varTheta + K) \pi }{2 \varTheta }} \right] t \right\} , \end{aligned}$$

where we have not simplified the expression any further so that the left and right eigenvectors and associated eigenvalues of $\mathbb {E}_0$ can be read off from this form. Because the filter threshold FPT densities are in general given by (cf. Eqs. (9b) and (9c))

46 $$\begin{aligned} G_{J; \, B}^{\pm \varTheta } (t) = \int _0^t \textrm{d} \tau \; \rho _B^\pm (t - \tau ) \, F_{J; \, B}^{\pm (\varTheta - 1)} (\tau ), \end{aligned}$$

or $\widehat{G}_{J; \, B}^{\pm \varTheta } (s) = \widehat{\rho }_B^\pm (s) \widehat{F}_{J; \, B}^{\pm (\varTheta - 1)} (s)$, which may be verified by directly solving the Laplace-transformed Eq. (17) (after setting $Y = \textrm{OFF}$ and summing over $X \in \{ \textrm{DOWN}, \textrm{OFF}, \textrm{UP} \}$) and using Eq. (21), we can just write down explicit forms for $G_{J; \, B}^{\pm \varTheta } (t)$ using Eq. (45). With the asymptotic approximation, the integral in Eq. (46) collapses, and the adiabatic approximation then gives

47 $$\begin{aligned} G_{J; \, B}^{\pm \varTheta } (t) = \langle r_B^\pm \rangle \, F_{J; \, B}^{\pm (\varTheta - 1)} (t). \end{aligned}$$

Although these are explicit solutions without using the Laplace transform, the Laplace-transformed versions are often simpler to use. With the asymptotic and adiabatic approximations, the results in Eq. (21) retain their given forms, but with $\varPhi _B^\pm (s)$ in Eq. (22) replaced by48The same replacement also occurs in Eq. (23) for $\widehat{\rho }_{0; \, B}^{\pm \varTheta } (s)$.

Although the filter dynamics collapse down to a Markov process with the asymptotic approximation, the strength dynamics do not collapse in this way. With the asymptotic and adiabatic approximations, for completeness we restate the master equation in Eq. (38) for strength states,

49 $$\begin{aligned} \frac{\textrm{d} P_{A; \, 0} (t)}{\textrm{d} t}&= \big ( \rho _{0; \, A - 1}^{+ \varTheta } *P_{A - 1; \, 0} \big ) (t) - \big ( \rho _{0; \, A}^{+ \varTheta } *P_{A; \, 0} \big ) (t) \nonumber \\&\quad + \big ( \rho _{0; \, A + 1}^{- \varTheta } *P_{A + 1; \, 0} \big ) (t) - \big ( \rho _{0; \, A}^{- \varTheta } *P_{A; \, 0} \big ) (t), \end{aligned}$$

where the Laplace transforms of $\rho _{0; \, B}^{\pm \varTheta }(t)$ are given in Eq. (23) (dropping the subscript OFF and adding the *B* index), with $\varPhi _B^\pm (s)$ given by Eq. (48). We have also stripped off the OFF label on $P_{A; \, 0, \textrm{OFF}} (t)$ to leave $P_{A; \, 0} (t)$, because of the asymptotic approximation. The two approximations have simplified the memory kernels $\rho _{0; \, B}^{\pm \varTheta }(t)$, but they are still present, so that the strength transitions continue to be governed by non- or semi-Markovian dynamics determined by non-exponential waiting times driven by the filter threshold FPT densities.

### Equilibrium strength distribution

As discussed, although we can solve Eq. (49) at least for the Laplace transform of $P_{A; \, 0}(t)$ and thus obtain the full time-dependence, we are more interested in the asymptotic behaviour of these probabilities, giving their equilibrium structure. With the adiabatic approximation in particular, the equilibrium limit is well-defined. Taking the Laplace transform of Eq. (49) and then again using the standard Tauberian theorem for the equivalence of the $s \rightarrow 0$ and $t \rightarrow \infty$ limits, the resulting equation for the equilibrium distribution, $P_{A; \, 0} (\infty )$, is

50 $$\begin{aligned} 0&= \left[ r_{0; \, A-1}^{+\varTheta } (\infty ) \, P_{A-1; \, 0} (\infty ) - r_{0; \, A}^{+\varTheta } (\infty ) \, P_{A; \, 0} (\infty ) \right] \nonumber \\&\quad + \left[ r_{0; \, A+1}^{-\varTheta } (\infty ) \, P_{A+1; \, 0} (\infty ) - r_{0; \, A}^{-\varTheta } (\infty ) \, P_{A; \, 0} (\infty ) \right] , \end{aligned}$$

with appropriate changes for the reflecting boundary conditions at $A=0$ and possibly at $A=N$. Because $r_{0; \, A}^{\pm \varTheta } (\infty ) = \widehat{\rho }_{0; \, A}^{\pm \varTheta } (0)$, from Eq. (23) we have the explicit forms

51 $$\begin{aligned} r_{0; \, A}^{\pm \varTheta } (\infty ) = \frac{\langle r_A^\pm \rangle ^\varTheta }{\varTheta } \frac{\langle r_A^+ \rangle - \langle r_A^- \rangle }{\langle r_A^+ \rangle ^\varTheta - \langle r_A^- \rangle ^\varTheta } \end{aligned}$$

for the coefficients in this recurrence relation for $P_{A; \, 0} (\infty )$. By swapping the positions of the two $r_{0; \, A}^{\pm \varTheta } (\infty ) \, P_{A; \, 0} (\infty )$ terms in Eq. (50), it is manifest that if the $P_{A; \, 0} (\infty )$ for $A > 0$ satisfy the relationship

52 $$\begin{aligned} P_{A; \, 0} (\infty ) = \frac{r_{0; \, A-1}^{+\varTheta } (\infty )}{r_{0; \, A}^{-\varTheta } (\infty )} P_{A-1; \, 0} (\infty ), \end{aligned}$$

then Eq. (50) is immediately satisfied. The $A=0$ boundary case $P_{\, 0; \, 0} (\infty )$ is used to normalise the distribution. This observation generates the usual solution for the equilibrium distribution of a general one-dimensional one-step random walk on a finite or semi-infinite set of states (van Kampen 1992), where for $A>0$,

53 $$\begin{aligned} P_{A; \, 0} (\infty ) = \frac{1}{r_{0; \, A}^{-\varTheta } (\infty )} \left[ \prod _{B=1}^{A-1} \frac{r_{0; \, B}^{+\varTheta } (\infty )}{r_{0; \, B}^{-\varTheta } (\infty )} \right] r_{0; \, 0}^{+\varTheta } (\infty ) \, P_{\, 0; \, 0} (\infty ). \end{aligned}$$

The product is absent for the $A = 1$ case.

**Fig. 3:**
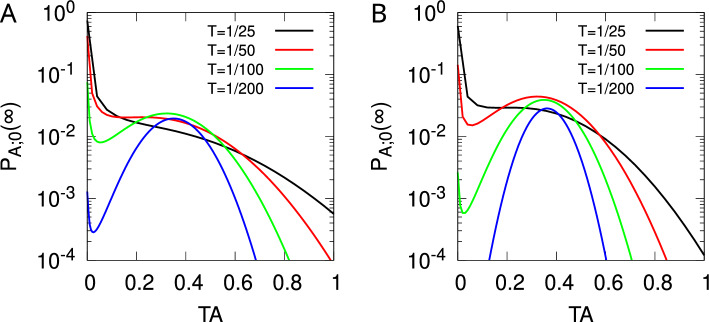
Equilibrium probability distribution of synaptic strength for a single afferent synapsing on a target cell. The distribution $P_{A; \, 0} (\infty )$ is plotted against the synapse’s strength *TA* for various choices of the plasticity step size *T* as indicated. In panel A, we take $\varTheta = 1$, while in panel B, we take $\varTheta = 2$. The strength *TA* takes only the discrete values dictated by the strength state *A* scaled by *T*, so that in the displayed interval [0, 1] there are $1 + 1/T$ discrete data points per line. The parameters are: $\mu = 50$ Hz, $\varsigma / \mu = 1/4$, $\lambda _- = 50$ Hz (or $\tau _- = 20$ ms), $\lambda _+ = \lambda _- / \rho$ where $\rho = 3/5$, and $\lambda _{\textrm{spont}} = 0.1$ Hz

In Fig. 3, we plot the equilibrium distribution of the strength of a single afferent synapsing on a target cell for two different choices of $\varTheta$ and different plasticity step sizes *T*. We see that for $\varTheta = 1$ and $T = 1/25$, the equilibrium distribution is tightly concentrated around the $A=0$ state. As *T* reduces, the distribution first broadens, then develops a maximum away from $A=0$, and finally sharpens up around this new maximum. A maximum of a probability distribution corresponds to a quasi-stable state that is frequently occupied by a system. If there are multiple maxima, then over time the system occupies all of them, with fluctuations driving transitions between them. As a distribution sharpens up around multiple maxima, the mean passage time for transitions between these maxima increases, possibly to the extent that any individual maximum is effectively stable because the fluctuations that drive transitions between the maxima develop on time scales that can for all practical purposes be regarded as infinitely long, or at least vastly longer than any relevant biological time scale. For $\varTheta = 2$, we observe the same behaviour as for $\varTheta = 1$, but a larger value of *T* for $\varTheta = 2$ roughly speaking corresponds to a smaller values of *T* for $\varTheta = 1$, in terms of the overall profile of the equilibrium probability distribution.

We have taken $\lambda _{\textrm{spont}} = 0.1$ Hz in Fig. 3. Using instead $\lambda _{\textrm{spont}} = 0$ Hz, the equilibrium distribution collapses down to $P_{A; \, 0} (\infty ) = \delta _{\, 0}^A$, so that it is entirely concentrated on the $A=0$, zero strength state. From Eq. (40), we see that if $\lambda _p = 0$ Hz, then $r_A^\pm = 0$ Hz, so that synaptic plasticity switches off in the switch model of STDP. In the absence of spontaneous postsynaptic activity, the postsynaptic spike firing rate can only become zero in the $A=0$ state. Hence, the $A=0$ state is an absorbing state in the absence of spontaneous postsynaptic activity, so we must allow a small, non-zero level of postsynaptic spontaneous activity to prevent this uninteresting case from arising. The equilibrium distribution is insensitive to the precise value of $\lambda _{\textrm{spont}}$ provided that it is not taken to be too large.

We denote by $T_{\textrm{C}}$ the critical value of *T* that corresponds to the first appearance of a maximum of $P_{A; \, 0} (\infty )$ for some $A > 0$ as *T* is reduced. This value $T_{\textrm{C}}$ corresponds to a value of *T* that induces a bifurcation in the number of maxima of $P_{A; \, 0} (\infty )$. For $\varTheta = 1$, we previously calculated an explicit expression for $T_{\textrm{C}}$ based on taking a continuum limit, regarding the synaptic strength $S=TA$ as a continuous rather than discrete quantity in Eq. (53), and making an approximation in replacing a sum by an integral in order to evaluate the logarithm of the product appearing in Eq. (53), and for simplicity using only $\lambda _\pi = \mu$ (Elliott 2011b). Repeating this calculation for general $\varTheta$, we find that

54 $$\begin{aligned} T_{\textrm{C}} = \varTheta \, \frac{\lambda _+}{4 \mu } \frac{\log _e^2 \frac{\lambda _+}{\lambda _- + \mu }}{1 + c \, \log _e \frac{\lambda _+}{\lambda _- + \mu }}, \end{aligned}$$

where

$$\begin{aligned} c&= 1 + \frac{\lambda _+ (\lambda _+ + \lambda _- + \mu )}{(\lambda _+ + \mu )(\lambda _- + \mu )} + \frac{\lambda _+}{\lambda _- + \mu - \lambda _+} \nonumber \\&\quad - \frac{\varTheta }{2} - \varTheta \, \frac{\lambda _+^\varTheta }{(\lambda _- + \mu )^\varTheta - \lambda _+^\varTheta }. \end{aligned}$$

For smaller values of $\varTheta$, $T_{\textrm{C}}$ scales nearly linearly with $\varTheta$, but as $\varTheta$ increases further, $T_{\textrm{C}}$ asymptotes to a value independent of $\varTheta$, where the asymptotic value is

55 $$\begin{aligned} T_{\textrm{C}} \rightarrow \frac{\lambda _+}{2 \mu } \left| \log _e \frac{\lambda _+}{\lambda _- + \mu } \right| . \end{aligned}$$

**Fig. 4:**
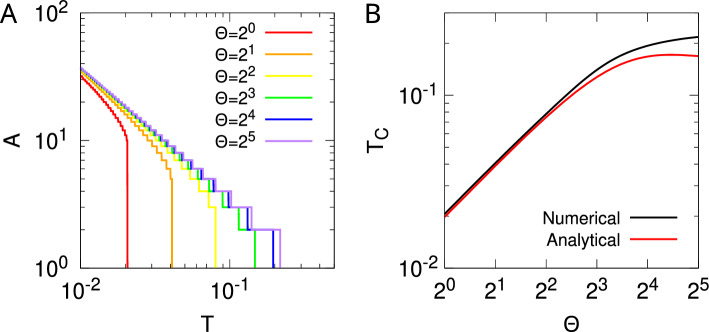
Scaling of critical synaptic plasticity step size with filter threshold for a single afferent synapsing on a target cell. In panel A, we plot the strength state $A > 0$ corresponding to a maximum of $P_{A; \, 0} (\infty )$ away from the zero strength state, as a function of the plasticity step size *T*, for different choices of threshold $\varTheta$ as indicated. At *T* is reduced, the value at which *A* suddenly jumps from $A=0$ to some value $A>0$ corresponds to a bifurcation. In panel B we plot this critical value $T_{\textrm{C}}$ as a function of $\varTheta$, where we obtain $T_{\textrm{C}}$ either numerically from the exact expression for $P_{A; \, 0} (\infty )$ (black line) or via the analytical approximation discussed in the main text (red line). Parameter choices correspond to those used in Fig. 3

We have observed this behaviour previously (Elliott 2011b) but not formally derived it because previously we only derived Eq. (54) for the specific, $\varTheta = 1$ case. Fig 4 shows the dependence of the location of the $A>0$ maximum of $P_{A; \, 0} (\infty )$ on *T*, and the dependence of the corresponding value of $T_{\textrm{C}}$ on $\varTheta$. For $\varTheta \lessapprox 8$, we see that the analytical expression for $T_{\textrm{C}}$ in Eq. (54) agrees very closely with the value of $T_{\textrm{C}}$ obtained by numerical methods. The agreement is still reasonable for larger values of $\varTheta$, but the approximation method used to obtain Eq. (54) requires considering both $S = TA$ and *T* to be small, so it starts to break down as $T_{\textrm{C}}$ increases above around 0.1.

We remark that in principle it is possible to obtain another approximation to the exact equilibrium solution in Eq. (53) by taking the continuum limit of the master equation in Eq. (38) and instead using the equilibrium solution of the resulting Fokker–Planck equation (van Kampen 1992). The explicit analytical form of this solution may be written down directly in terms of the asymptotic rates in Eq. (51). A very simple condition on the location of its extrema may be obtained, with bifurcations in the number of extrema determining $T_{\textrm{C}}$. The resulting solutions for $T_{\textrm{C}}$ are in decent agreement with the results from the master equation above for $\varTheta \le 9$, but for larger values of $\varTheta$ the results from the Fokker–Planck equation grow rather than saturate. In this context, the Fokker–Planck is an approximation to the master equation that depends critically on the plasticity step size *T* being small, so that the master equation can be truncated at second order in *T* to generate the Fokker–Planck equation. For *T* and thus $T_{\textrm{C}}$ in excess of again around 0.1, the Fokker–Planck equation ceases to be a useful approximation to the master equation. Because the asymptotic value of $T_{\textrm{C}}$ in Fig. 4B and Eq. (55) is around 0.2 for the parameters used here, the Fokker–Planck equation is therefore unable to probe this regime.

## Beyond one afferent

For a single afferent, Eq. (38) is exact, with no approximations having been made in deriving it. Even with the asymptotic and adiabatic approximations made in writing down Eq. (49) with its modified memory kernels, the equation captures the key dynamics occurring on multiple time scales that lead to changes in a single afferent’s synaptic strength. However, neurons typically receive synaptic input from multiple afferents. We are particularly interested in the outcome of development in the primary visual cortex, in which V1 neurons receive synaptic input via the lateral geniculate nucleus from retinal ganglion cells in both eyes. During development, activity-dependent synaptic competition leads from an initial state of roughly equal control of V1 neurons by both eyes to the emergence of a final state of ocular dominance columns, in which one eye or the other controls any given V1 neuron, but typically with spatial organisation to this control across V1 (Purves and Lichtman 1985).

Previously, we modified Eq. (49) to accommodate multiple afferents. With *m* afferents, we write their strength states $A_i$ (or $B_i$), $i = 1, \ldots , m$, as the vector $\underline{A}$ (or $\underline{B}$), and denote the set of *m* Cartesian basis vectors by $\{ \underline{\varDelta }^1, \ldots , \underline{\varDelta }^m \}$, where $\underline{\varDelta }^i_j = \delta ^i_j$, so that the vector $\underline{\varDelta }^i$ has unity at position *i* and zero elsewhere. With afferent spike firing rates $\lambda _i$ written in vector form $\underline{\lambda }$, we modify Eq. (32) in the obvious way for $\underline{B} \ne \underline{0}$ so that $\lambda _p = T \underline{\lambda } \cdot \underline{B}$. The $m = 1$ induction rates $r^\pm _B$ relate to one afferent, while for general *m* we must write them as $r^\pm _{\underline{B}}(\lambda _i)$, so that in Eq. (40), $\lambda _\pi$ is replaced by the specific $\lambda _i$, and $\lambda _p$ of course depends on $\underline{\lambda }$ and $\underline{B}$. We need not denote this additional dependence of $r^\pm _{\underline{B}}(\lambda _i)$ on the full vector $\underline{\lambda }$, but just its dependence on the specific afferent *i* of interest via $\lambda _i$. Similarly, the functions $\rho _{0; \, A}^{\pm \varTheta }(t)$ must become $\rho _{0; \, \underline{A}}^{\pm \varTheta } (\lambda _i; t)$ to indicate the particular afferent *i*, and in convolutions the temporal argument will be stripped off to leave $\rho _{0; \, \underline{A}}^{\pm \varTheta } (\lambda _i)$. We then modified Eq. (49) for $m = 1$ afferent so that it becomes

56 $$\begin{aligned}&\frac{\textrm{d} P_{\, \underline{A}; \, \underline{0}}(t)}{\textrm{d} t} =\sum _{i=1}^m \Big [ \big ( \rho ^{+\varTheta }_{0; \, \underline{A} - \underline{\varDelta }^i} (\lambda _i) *P_{\, \underline{A} - \underline{\varDelta }^i; \, \underline{0}} \big ) (t) \nonumber \\&\quad - \big ( \rho ^{+\varTheta }_{0; \, \underline{A}}(\lambda _i) *P_{\, \underline{A}; \, \underline{0}} \big ) (t) \nonumber \\&\quad + \big ( \rho ^{-\varTheta }_{0; \, \underline{A} + \underline{\varDelta }^i} (\lambda _i) *P_{\, \underline{A} + \underline{\varDelta }^i; \, \underline{0}} \big ) (t) \nonumber \\&\quad - \big ( \rho ^{-\varTheta }_{0; \, \underline{A}} (\lambda _i) *P_{\, \underline{A}; \, \underline{0}} \big ) (t) \Big ], \end{aligned}$$

which is just our previous Eq. (3.4) in Elliott (2011b) in our present notation. Changes to accommodate the reflecting boundary conditions for each afferent can be made as usual. Writing down this equation entails a massively simplifying approximation: that a filter threshold event in one synapse will reset the filters in all the other synapses. In fact, a further, then-unrecognised approximation is also present, as we shall discuss. Although this resetting approximation is coarse and gross, it permits much analytical insight and generates results that are in surprisingly good agreement with simulation results (Elliott 2011b). Our principal purpose here is to improve on Eq. (56) and in the process understand in detail why Eq. (56) works so well. For tractability, both analytical and numerical, we must restrict to $m = 2$ afferents. This is sufficient for the study of ocular dominance, but the $m > 2$ case is in any event essentially equivalent to the $m = 2$ case in terms of how fluctuations drive transitions between different segregated states (Elliott 2011b).

### STDP in two dimensions

**Fig. 5:**
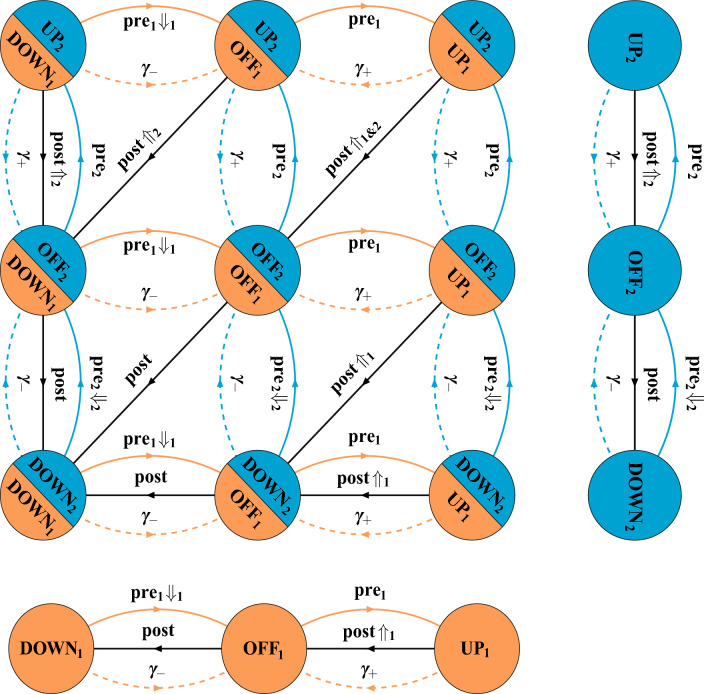
Joint synaptic state transitions induced by spike-timing-dependent plasticity in the tristate switch model for two afferents’ synapses. The format of this figure is essentially identical to that for Fig. 1A for one synapse, except that here we must represent the state of both synapses’ separate switches. Subscripts are added to the DOWN, OFF and UP states to indicate the afferent; to the presynaptic spike processes corresponding to the relevant afferent; and to the induction arrows $\Uparrow$ and $\Downarrow$ to indicate to which synapse the induction signal applies ($1 \& 2$ meaning both afferents). Different colours are also used for additional clarity. As well as the full transitions between all nine joint states in the top-left diagram, we also show in the right and bottom diagrams the marginal transitions for each synapse separately, so ignoring the transitions in the other synapse’s switch state. These marginal transitions of course correspond to those shown in Fig. 1A. For a joint transition diagram equivalent to Fig. 1B, we would require eleven fictitious absorbing states in the top-left diagram, replacing the eleven transitions that induce plasticity with transitions into the corresponding absorbing states

Although the spike firing rates $\lambda _1$ and $\lambda _2$ of two afferents may be subject to correlations $\mathfrak {r}_{12}$ due to commonalities in their stimulus patterns, we assume that the precise timing of their spikes is uncorrelated, so that the Poisson processes for their spikes are uncorrelated. However, both afferents’ synapses on the same target cell will always experience the same sequence of postsynaptic spikes. The postsynaptic spike train experienced by both afferents can therefore lead to simultaneous changes in both afferents’ separate STDP switch states. Although each afferent’s synapse possesses a separate STDP switch with three possible states, we must therefore consider the possible transitions between the nine states representing the $3 \times 3$ possible combinations of both synapses’ switch states. Figure 5 represents the possible transitions between these nine joint states. Of particular note are the four postsynaptic-spike-induced transitions that occur in the joint diagram in the diagonal direction from top-right to bottom-left. These four correspond to transitions in which both synapses’ STDP switch states change simultaneously. The transition $\textrm{UP}_1 \textrm{UP}_2 \rightarrow \textrm{OFF}_1 \textrm{OFF}_2$ in particular induces the simultaneous potentiation of both synapses’ strengths. The other four postsynaptic-spike-induced transitions in the joint diagram do not cause simultaneous switch state changes because at least one of the synapses’ switch states is DOWN, which by assumption does not respond to a postsynaptic spike.

We employ the same asymptotic approximation that we used for a single synapse. Both synapse’s induction rates attain their asymptotic behaviours swiftly, so we use only these asymptotic induction rates, where they may be obtained by regarding $\lambda _1$ and $\lambda _2$ as fixed because these latter change more slowly. We may then calculate the asymptotic induction rates directly from the equilibrium distribution of the afferents’ joint switch states. Ordering these joint switch states as $\{ \textrm{DOWN}_1 \textrm{DOWN}_2, \textrm{DOWN}_1 \textrm{OFF}_2, \textrm{DOWN}_1 \textrm{UP}_2, \textrm{OFF}_1 \textrm{DOWN}_2, \textrm{OFF}_1 \textrm{OFF}_2, \textrm{OFF}_1 \textrm{UP}_2, \textrm{UP}_1 \textrm{DOWN}_2, \textrm{UP}_1 \textrm{OFF}_2, \textrm{UP}_1 \textrm{UP}_2 \}$, the generating matrix governing the joint transitions in Fig. 5 is given by:57where each of the “$\ominus$” symbols on the diagonal should be replaced with that quantity that ensures that the corresponding column sum is zero (so that, e.g. $[ \, \mathbb {S} \, ]_{2,2} = - (\lambda _1 + \lambda _2 + \lambda _p + \lambda _-)$). The vertical and horizontal bars in Eq. (57) are used to partition the matrix into the $3 \times 3$ submatrices corresponding to particular switch states of the first afferent’s synapse. For clarity, we have used colour in the matrix elements in Eq. (57) to indicate only those transitions that induce synaptic plasticity, with red (respectively, blue) indicating transitions that affect only the first (respectively, second) afferent, and green indicating the transition in which both afferents experience simultaneous potentiation signals. The joint evolution of the two synapses’ switch states is determined by the usual matrix exponential, with the equilibrium distribution $\underline{\sigma }$ being given by the normalised null eigenvector of $\mathbb {S}$. The expression for the equilibrium distribution is extremely messy, so we do not reproduce it here. However, it is easy to explicitly verify that

58a $$\begin{aligned}&\lambda _1 \left( \sigma _{\, \textrm{DOWN}_1 \textrm{DOWN}_2} + \sigma _{\, \textrm{DOWN}_1 \textrm{OFF}_2} + \sigma _{\, \textrm{DOWN}_1 \textrm{UP}_2} \right) \nonumber \\ &\quad = r_{\underline{B}}^-(\lambda _1), \end{aligned}$$

58b $$\begin{aligned}&\lambda _2 \left( \sigma _{\, \textrm{DOWN}_1 \textrm{DOWN}_2} + \sigma _{\, \textrm{OFF}_1 \textrm{DOWN}_2} + \sigma _{\, \textrm{UP}_1 \textrm{DOWN}_2} \right) \nonumber \\ &\quad = r_{\underline{B}}^-(\lambda _2), \end{aligned}$$

where in each of these equations, we marginalise over one afferent to obtain the marginal rate of depressing induction signals of the other afferent. Similarly, we may verify that

59a $$\begin{aligned}&\lambda _p \left( \sigma _{\, \textrm{UP}_1 \textrm{DOWN}_2} + \sigma _{\, \textrm{UP}_1 \textrm{OFF}_2} + \sigma _{\, \textrm{UP}_1 \textrm{UP}_2} \right) = r_{\underline{B}}^+(\lambda _1), \end{aligned}$$

59b $$\begin{aligned}&\lambda _p \left( \sigma _{\, \textrm{DOWN}_1 \textrm{UP}_2} + \sigma _{\, \textrm{OFF}_1 \textrm{UP}_2} + \sigma _{\, \textrm{UP}_1 \textrm{UP}_2} \right) = r_{\underline{B}}^+(\lambda _2). \end{aligned}$$

As indicated, however, for these joint dynamics, a new process arises in which both afferents generate a postsynaptic-spike-triggered potentiating induction event simultaneously, from the $\textrm{UP}_1 \textrm{UP}_2 \rightarrow \textrm{OFF}_1 \textrm{OFF}_2$ transition. We denote the rate of this doubly potentiating induction process by $r_{\underline{B}}^{\, \ddagger } (\underline{\lambda })$, where $r_{\underline{B}}^{\, \ddagger } (\underline{\lambda }) = \lambda _p \, \sigma _{\, \textrm{UP}_1 \textrm{UP}_2}$. For completeness we give its explicit though messy form, which is

60 $$\begin{aligned} r_{\underline{B}}^{\, \ddagger } (\underline{\lambda }) = \frac{r_{\underline{B}}^+(\lambda _1) \, r_{\underline{B}}^+(\lambda _2)}{\lambda _p} \, \frac{a}{b}, \end{aligned}$$

with

$$\begin{aligned} a&= 2 \big [ (\lambda _p + \lambda _+) (\lambda _p + \lambda _-) - \lambda _1 \lambda _2 \big ] \lambda _p \nonumber \\&\quad + \big [ (\lambda _{12} + 2 \lambda _p + 2 \lambda _+) (\lambda _{12} + 2 \lambda _p + 2 \lambda _-) - \lambda _{12} \lambda _p \big ] \nonumber \\&\hspace{3cm} \times (\lambda _{12} + \lambda _p + \lambda _+ + \lambda _-) \\ b&= (\lambda _{12} + \lambda _p + 2 \lambda _+) (\lambda _{12} + \lambda _p + 2 \lambda _-) (\lambda _{12} {+} \lambda _p {+} \lambda _+ {+} \lambda _-)\nonumber \\&\quad - 2 \lambda _1 \lambda _2 \lambda _p, \end{aligned}$$

where we write $\lambda _{12} = \lambda _1 + \lambda _2$ for brevity. In the marginal rates $r_{\underline{B}}^+(\lambda _1)$ and $r_{\underline{B}}^+(\lambda _2)$ in Eq. (59), we note the appearance of $\lambda _p \, \sigma _{\, \textrm{UP}_1 \textrm{UP}_2} = r_{\underline{B}}^{\, \ddagger }(\underline{\lambda })$ on the left-hand sides of both expressions. These marginal potentiating processes are therefore not independent because they both involve the doubly potentiating process with rate $r_{\underline{B}}^{\, \ddagger }(\underline{\lambda })$. The two independent processes that potentiate the two afferents separately arise from just the $\lambda _p ( \sigma _{\, \textrm{UP}_1 \textrm{DOWN}_2} + \sigma _{\, \textrm{UP}_1 \textrm{OFF}_2} )$ and $\lambda _p ( \sigma _{\, \textrm{DOWN}_1 \textrm{UP}_2} + \sigma _{\, \textrm{OFF}_1 \textrm{UP}_2} )$ contributions to Eq. (59). Hence, there are three independent potentiating processes involving the two afferents: one with rate $r_{\underline{B}}^+ (\lambda _1) - r_{\underline{B}}^{\, \ddagger }(\underline{\lambda })$ involving only the first afferent; one with rate $r_{\underline{B}}^+ (\lambda _2) - r_{\underline{B}}^{\, \ddagger }(\underline{\lambda })$ involving only the second afferent; and the doubly potentiating process with rate $r_{\underline{B}}^{\, \ddagger } (\underline{\lambda })$ involving both afferents. For the marginal depressing rates in Eq. (58), there is no common process shared between them (the common $\sigma _{\, \textrm{DOWN}_1 \textrm{DOWN}_2}$ term is multiplied by different presynaptic rates, either $\lambda _1$ or $\lambda _2$, and therefore correspond to different transition processes), so the two marginal rates $r_{\underline{B}}^- (\lambda _1)$ and $r_{\underline{B}}^- (\lambda _2)$ correspond to independent processes involving just the first and second afferent, respectively. Overall, then, we have five independent induction processes, four of which involve only a single afferent, with the fifth involving both.

### Synaptic filtering in two dimensions

These five different independent induction processes involving the two afferents will induce changes in the two afferents’ filter states. As for the single afferent case, we again assume that these rates affect filter transitions only via their ensemble average over stimulus patterns. We denote the joint filter state of both afferents by vectors such as $\underline{I}$ and $\underline{J}$, where $\underline{I} = ( I_1, I_2 )^{\textrm{T}}$, with $I_i$ being the filter state of afferent *i*. For notational simplicity it is often convenient not to distinguish between row and column vectors below, so we will often write just $( I_1, I_2 )$, without the transpose, as the joint filter state. Both synapses are assumed to have the same filter thresholds $\pm \varTheta$.

**Fig. 6:**
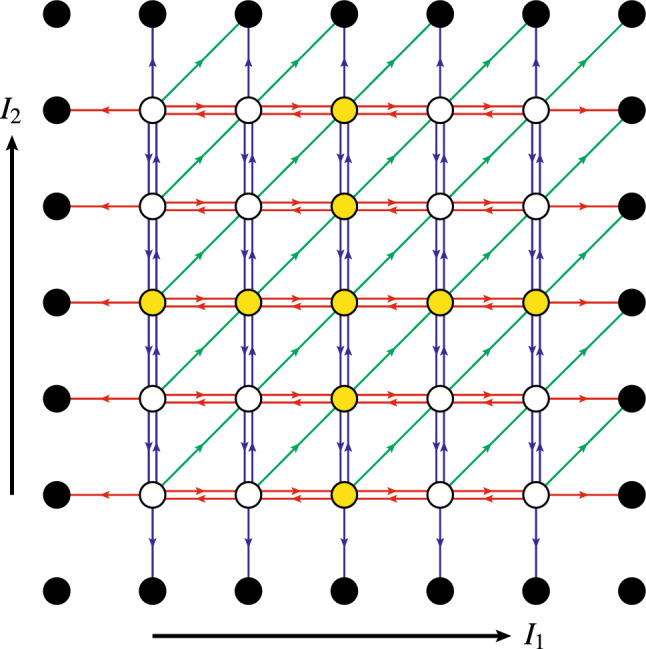
Schematic illustration of the transitions in the joint filter states of two afferents’ synapses induced by the switch model of STDP. We set $\varTheta = 3$ for concreteness. The white or yellow circles indicate occupiable joint filter states $(I_1, I_2)$ with $| I_i | < \varTheta$, with the yellow circles highlighting those states in which $I_1 = 0$ or $I_2 = 0$. The black circles form a square boundary around the occupiable states and indicate the absorbing threshold filter states in which $I_1 = \pm \varTheta$ or $I_2 = \pm \varTheta$. Red lines indicate filter transitions involving only the first afferent, with potentiating signal rates $\langle r_{\underline{B}}^+ (\lambda _1) - r_{\underline{B}}^{\, \ddagger } (\underline{\lambda }) \rangle$ (rightward transitions) and depressing signal rates $\langle r_{\underline{B}}^- (\lambda _1) \rangle$ (leftward transitions). Blue lines indicate filter transitions involving only the second afferent, with potentiating signal rates $\langle r_{\underline{B}}^+ (\lambda _2) - r_{\underline{B}}^{\, \ddagger } (\underline{\lambda }) \rangle$ (upward transitions) and depressing signal rates $\langle r_{\underline{B}}^- (\lambda _2) \rangle$ (downward transitions). The diagonal green lines indicate filter transitions in which both afferents simultaneously experience potentiating induction signals, at rate $\langle r_{\underline{B}}^{\, \ddagger } (\underline{\lambda }) \rangle$. For clarity we have not indicated the resetting transitions in which a filter state is reset to the zero state at threshold. Of the four corner threshold states $(+\varTheta ,+\varTheta )$, $(+\varTheta ,-\varTheta )$, $(-\varTheta ,-\varTheta )$ and ($-\varTheta ,+\varTheta$) in which both filters reach threshold simultaneously, only the top-right corner, corresponding to $(+\varTheta ,+\varTheta )$, is attainable, via a diagonal, doubly potentiating transition

Figure 6 illustrates the possible filter state transitions induced by STDP involving both afferents’ synapses. For clarity, the transitions leading to the resetting of a synapse’s filter state to zero upon reaching threshold are not shown. Of course, one synapse may reach threshold and reset its filter state to zero while the other may not. Threshold of one or both synapses is reached on the absorbing boundary (the black circles in Fig. 6) defined by the set

61 $$\begin{aligned} \mathfrak {B} = \{&(+\varTheta ,+\varTheta ), \ldots , (+\varTheta ,-(\varTheta {-}1)), \nonumber \\&(+\varTheta ,-\varTheta ), \ldots , (-(\varTheta {-}1),-\varTheta ), \nonumber \\&(-\varTheta ,-\varTheta ), \ldots , (-\varTheta ,+(\varTheta {-}1)), \nonumber \\&(-\varTheta ,+\varTheta ), \ldots , (+(\varTheta {-}1),+\varTheta )\}, \end{aligned}$$

containing $8 \varTheta$ boundary points. These boundary points are enumerated clockwise starting from the upper right-hand corner $(+\varTheta ,+\varTheta )$ of the square boundary. When a synapse reaches threshold, its filter state is reset to zero, but of course the other synapse’s filter state remains unchanged (unless both reach threshold simultaneously). Thus, upon reaching the possible threshold configurations $(\pm \varTheta , I_2)$ or $(I_1, \pm \varTheta _1)$, the joint filter state always returns to the $(0, I_2)$ or $(I_1, 0)$ joint filter states, respectively. These states are coloured yellow in Fig. 6. They resemble cross-hairs in an eyepiece, so we refer to them as cross-hair filter states. At least one filter state is always zero in these cross-hair states. We denote the set of cross-hair states by $\mathfrak {X}$, where

62 $$\begin{aligned} \mathfrak {X} = \mathfrak {H} \cup \mathfrak {V} \cup \mathfrak {Z}, \end{aligned}$$

with

$$\begin{aligned} \begin{array}{lcl} \mathfrak {H} & \! = \! & \{ (-(\varTheta {-}1),0), \ldots , (-1, 0), (+1, 0), \ldots , (+(\varTheta {-}1), 0) \}, \\ \mathfrak {V} & \! = \! & \{ (0, -(\varTheta {-}1)), \ldots , (0, -1), (0, +1), \ldots , (0,+(\varTheta {-}1)) \}, \\ \mathfrak {Z} & \! = \! & \{ (0, 0) \}. \end{array} \end{aligned}$$

The set $\mathfrak {H}$ contains the “horizontal” states $(I_1, 0)$ excluding $I_1 = 0$; $\mathfrak {V}$ contains the “vertical” states $(0, I_2)$ excluding $I_2 = 0$; and the singleton set $\mathfrak {Z}$ contains the “bull’s eye” of the cross-hairs at (0, 0).

Starting from any initial joint filter state $\underline{J}$, the filter dynamics may in general escape through any of the boundary points in $\mathfrak {B}$, although three of the corner points are not attainable because the required diagonal transitions do not exist. The joint filter state will then be reset to the relevant cross-hair state in $\mathfrak {X}$. We must compute the escape densities from initial filter state $\underline{J}$ through any of these boundary points. Let $\underline{\mathfrak {I}} \in \mathfrak {B}$ be any one of these boundary sites. Then, the escape density $G^{\, \underline{\mathfrak {I}}}_{\underline{J}; \, \underline{B}}(t)$ for escape through $\underline{\mathfrak {I}}$ starting from $\underline{J}$ satisfies the differential equation

63 $$\begin{aligned} \frac{\textrm{d} G^{\, \underline{\mathfrak {I}}}_{\underline{J}; \, \underline{B}}(t)}{\textrm{d} t}&= \big [ G^{\, \underline{\mathfrak {I}}}_{\underline{J} + \underline{\varDelta }^1; \, \underline{B}} (t) - G^{\, \underline{\mathfrak {I}}}_{\underline{J}; \, \underline{B}} (t) \big ] \langle r_{\underline{B}}^+(\lambda _1) - r_{\underline{B}}^{\,\ddagger }(\underline{\lambda }) \rangle \nonumber \\&\quad + \big [ G^{\, \underline{\mathfrak {I}}}_{\underline{J} + \underline{\varDelta }^2; \, \underline{B}} (t) - G^{\, \underline{\mathfrak {I}}}_{\underline{J}; \, \underline{B}} (t) \big ] \langle r_{\underline{B}}^+(\lambda _2) - r_{\underline{B}}^{\, \ddagger }(\underline{\lambda }) \rangle \nonumber \\&\quad + \big [ G^{\, \underline{\mathfrak {I}}}_{\underline{J} + \underline{\varDelta }^{\! \times \! \!}; \, \underline{B}} (t) - G^{\, \underline{\mathfrak {I}}}_{\underline{J}; \, \underline{B}}(t) \big ] \langle r_{\underline{B}}^{\, \ddagger }(\underline{\lambda }) \rangle \nonumber \\&\quad + \big [ G^{\, \underline{\mathfrak {I}}}_{\underline{J} - \underline{\varDelta }^1; \, \underline{B}} (t) - G^{\, \underline{\mathfrak {I}}}_{\underline{J}; \, \underline{B}} (t) \big ] \langle r_{\underline{B}}^-(\lambda _1) \rangle \nonumber \\&\quad + \big [ G^{\, \underline{\mathfrak {I}}}_{\underline{J} - \underline{\varDelta }^2; \, \underline{B}} (t) - G^{\, \underline{\mathfrak {I}}}_{\underline{J}; \, \underline{B}}(t) \big ] \langle r_{\underline{B}}^-(\lambda _2) \rangle , \end{aligned}$$

subject to the absorbing boundary conditions $G^{\, \underline{\mathfrak {I}}}_{\underline{\mathfrak {I}}; \, \underline{B}} (t) = \delta (t)$ and $G^{\, \underline{\mathfrak {I}}}_{\underline{\mathfrak {I}}'; \, \underline{B} } (t) = 0$ $\forall \underline{\mathfrak {I}}' \in \mathfrak {B}$ with $\underline{\mathfrak {I}}' \ne \underline{\mathfrak {I}}$. We have defined $\underline{\varDelta }^{\! \times \! \!} = \underline{\varDelta }^1 + \underline{\varDelta }^2 = (1,1)$, giving the simultaneous change in both filter states induced by the induction process with rate $\langle r_{\underline{B}}^{\, \ddagger }(\underline{\lambda }) \rangle$. There are in general $8 \varTheta (2 \varTheta - 1)^2$ of these densities $G^{\, \underline{\mathfrak {I}}}_{\underline{J}; \, \underline{B}}(t)$, corresponding to escape through any one of the $8 \varTheta$ boundary sites starting from any one of the $(2 \varTheta - 1)^2$ initial joint filter states. The one-dimensional equivalents $G_{J; \, B}^{\pm \varTheta }(t)$ number just $2 (2 \varTheta -1)$. For the particular case that $r_{\underline{B}}^{\, \ddagger } (\underline{\lambda }) = 0$ Hz, we have two independent random walks between absorbing boundaries in each direction, so we can just write down the two-dimensional escape functions in terms of the one-dimensional escape functions:64(Here the one-dimensional escape functions nevertheless depend on the full strength state $\underline{B}$ because of the dependence of $\lambda _p$ on $\underline{B}$; they do not depend on just either $B_1$ or $B_2$.) However, in the presence of the diagonal process, we typically cannot obtain closed-form expressions for the $G^{\, \underline{\mathfrak {I}}}_{\underline{J}; \, \underline{B}} (t)$ nor their Laplace transforms. Fortunately, as we will only be concerned with the equilibrium distribution of a pair of afferents’ synaptic strengths, we do not need the full forms for $G^{\, \underline{\mathfrak {I}}}_{\underline{J}; \, \underline{B}} (t)$. As $\widehat{G}^{\, \underline{\mathfrak {I}}}_{\underline{J}; \, \underline{B}} (-s)$ is just the MGF of the FPT density through the boundary site $\underline{\mathfrak {I}}$ starting from joint filter state $\underline{J}$, we can write

65 $$\begin{aligned} \widehat{G}^{\, \underline{\mathfrak {I}}}_{\underline{J}; \, \underline{B}}(-s) = \pi ^{\, \underline{\mathfrak {I}}}_{\underline{J}; \, \underline{B}} \big [ 1 + s \, \tau ^{\, \underline{\mathfrak {I}}}_{\underline{J}; \, \underline{B}} + \mathscr {O}(s^2) \big ], \end{aligned}$$

where the $8 \varTheta$ functions $\pi ^{\, \underline{\mathfrak {I}}}_{\underline{J}; \, \underline{B}}$ and $\tau ^{\, \underline{\mathfrak {I}}}_{\underline{J}; \, \underline{B}}$ for fixed joint filter state $\underline{J}$ are the splitting probabilities and conditional mean escape times (or splitting times), respectively, for escape (see, e.g. van Kampen 1992) through the $8 \varTheta$ boundary sites from the state $\underline{J}$. We can plug this expansion into the expression for the derivatives of the escape rates,

66 $$\begin{aligned} \widehat{\rho }^{\, \underline{\mathfrak {I}}}_{\underline{J}; \, \underline{B}} (s) = \frac{s \, \widehat{G}^{\, \underline{\mathfrak {I}}}_{\underline{J}; \, \underline{B}} (s)}{1 - \sum _{\underline{\mathfrak {I}}' \in \mathfrak {B}} \widehat{G}^{\, \underline{\mathfrak {I}}'}_{\underline{J}; \, \underline{B}} (s)}, \end{aligned}$$

and then take the limit $s \rightarrow 0$ to obtain the asymptotic escape rate $r^{\, \underline{\mathfrak {I}}}_{\underline{J}; \, \underline{B}}(\infty )$. We obtain the standard result

67 $$\begin{aligned} r^{\, \underline{\mathfrak {I}}}_{\underline{J}; \, \underline{B}}(\infty ) = \frac{\pi ^{\, \underline{\mathfrak {I}}}_{\underline{J}; \, \underline{B}}}{\sum _{\underline{\mathfrak {I}}' \in \mathfrak {B}} \pi ^{\, \underline{\mathfrak {I}}'}_{\underline{J}; \, \underline{B}} \, \tau ^{\, \underline{\mathfrak {I}}'}_{\underline{J}; \, \underline{B}}}, \end{aligned}$$

where the denominator is the total mean escape time through any of the $8 \varTheta$ boundary points, starting from $\underline{J}$. By Laplace transforming Eq. (63) and expanding to first order in *s*, we obtain at zeroth order a set of coupled linear equations solely in the splitting probabilities and then at first order a set of coupled linear equations for the splitting times involving the splitting probabilities. The two sets of equations can be solved successively by standard numerical methods to give the splitting probabilities and thence times, and hence, we can obtain the asymptotic escape rates $r^{\, \underline{\mathfrak {I}}}_{\underline{J}; \, \underline{B}}(\infty )$ numerically.

### Expressing changes in joint synaptic strength

We can now write down a renewal equation for the transition probability for a change in the joint synaptic filter and strength states of both synapses. Denoting this transition probability by $P_{\underline{B}, \underline{J}}^{\, \underline{A}, \underline{I}}(t)$, the renewal equation can be written in the compact form68where $F_{\underline{J}; \, \underline{B}}^{\, \underline{I}}(t)$ is the probability of a change in the joint filter state without any threshold process having occurred in time *t*, with the usual relationship between $F_{\underline{J}; \, \underline{B}}^{\, \underline{I}}(t)$ and $G_{\underline{J}; \, \underline{B}}^{\, \underline{\mathfrak {I}}} (t)$:

69 $$\begin{aligned} \sum _{\underline{I}} F_{\underline{J}; \, \underline{B}}^{\, \underline{I}} (t) = 1 - \sum _{\underline{\mathfrak {I}} \in \mathfrak {B}} \int _0^t \textrm{d} \tau \, G_{\underline{J}; \, \underline{B}}^{\, \underline{\mathfrak {I}}} (\tau ). \end{aligned}$$

In Eq. (68), the projection operator  acts on a threshold boundary state $\underline{\mathfrak {I}} \in \mathfrak {B}$ to project $\underline{\mathfrak {I}}$ to the relevant cross-hair filter state , where for $| \mathfrak {I}_i | < \varTheta$,70where all four sign combinations give the corner boundary sites in the final case. The projection operator  just encodes filter resetting following threshold events. The operator  similarly encodes the associated change in synaptic strength state at filter threshold, with71where again all four sign combinations are possible in the final case, and we have given the three unattainable corner cases for completeness. If  is such that a strength state $B_i$ falls below $B_i = 0$ or rises above the possible maximum allowed state of $B_i = N$, then it is just reflected back to $\underline{B}$ in Eq. (68) so that saturation is correctly implemented. The sum over the homogeneous terms on the right-hand side of Eq. (68) simply corresponds to a sum over all possible filter threshold processes in the boundary set $\mathfrak {B}$ (the three unattainable corner sites having escape densities of exactly zero), leading to all possible changes in the initial synaptic strength state $\underline{B}$ following the first filter threshold process at time $0< \tau < t$.

Writing $\mathscr {P}_{\underline{B}, \, \underline{J}}^{\, \underline{A}} (t) = \sum _{\underline{I}} P_{\underline{B}, \, \underline{J}}^{\, \underline{A}, \, \underline{I}} (t)$, by the usual manipulations we obtain from Eq. (68) the backward equation72This should be compared to the equivalent equation for the $m = 1$ afferent case, which from Eq. (33) is73where now $\mathfrak {B}_1 = \{ -\varTheta , +\varTheta \}$ contains just the two filter threshold boundaries at $\pm \varTheta$; the one-dimensional projection operator takes the trivial form ; and . For the $m = 1$ afferent case, the right-hand side contains $\mathscr {P}_{B {\pm } 1, \, 0}^{\, A}(t - \tau )$, and hence, we could consider just the $J = 0$ initial filter state, with the underlying zero $\rightarrow$ zero filter transitions being the key processes driving changes in synaptic strength state. However, for the $m = 2$ afferent case in Eq. (72), the range of the two-dimensional projection operator  is the entire set of cross-hair filter states $\mathfrak {X}$, containing $4 \varTheta - 3$ elements, and not just the single element (0, 0) (unless $\varTheta = 1$). For two afferents, the strength-change dynamics are therefore driven not by zero $\rightarrow$ zero transitions, but by cross-hair $\rightarrow$ cross-hair transitions. Taking any $\underline{J} \in \mathfrak {X}$ and with  for any $\underline{\mathfrak {I}} \in \mathfrak {B}$, Eq. (72) constitutes a closed system of equations in the joint strength state transition probabilities starting from any of the $4 \varTheta - 3$ cross-hair filter states. The transition probabilities starting from any of the other $4 (\varTheta - 1)^2$ initial joint filter states are completely determined by these $4 \varTheta - 3$ cross-hair states. Since we are principally interested in the long-term behaviour of transition probabilities or strength states, we can again without loss of generality just take $\underline{J}$ to be any element of the cross-hair filter states $\mathfrak {X}$.

For the $m = 1$ afferent case, we could not move to the forward equation for $\mathscr {P}_B^{\, A} (t) = \sum _{I} P_{B, 0}^{\, A, I} (t)$ (actually $\sum _{I, X} P_{B, 0, \textrm{OFF}}^{\, A, I, X} (t)$, but with the asymptotic approximation, we can throw away reference to the switch states). Similarly, for $m = 2$ afferents we cannot move to the forward equation for $\mathscr {P}_{\underline{B}}^{\, \underline{A}} (t) = \sum _{\underline{I}} P_{\underline{B}, \underline{0}}^{\, \underline{A}, \underline{I}} (t)$. For $m = 1$ afferent, we established a correspondence between $\mathscr {P}_B^{\, A} (t)$ and $P_B^{\, A} (t)$ by elevating the memory kernels $\rho _{0; \, B}^{\pm \varTheta } (t)$ to a fundamental role as the drivers of a one-step random walk in strength space. We must do the same for two afferents. We therefore consider a random walk on the two-dimensional space of synaptic strength states by defining the memory kernels $\rho _{\underline{J}; \, \underline{B}}^{\, \underline{\mathfrak {I}}} (t)$ for $\underline{J} \in \mathfrak {X}$ as the (in general) non-exponentially distributed processes that drive changes in state. A state is specified by a strength state $\underline{A}$ and a cross-hair filter state $\underline{I} \in \mathfrak {X}$. The backward Chapman–Kolmogorov equation is then just74By summing over $\underline{I} \in \mathfrak {X}$, we obtain an equation identical to Eq. (72), establishing the correspondence between $\sum _{\underline{I} \in \mathfrak {X}} P_{\underline{B}; \, \underline{J}}^{\, \underline{A}; \, \underline{I}} (t)$ and $\sum _{\underline{I}} P_{\underline{B}, \, \underline{J}}^{\, \underline{A}, \, \underline{I}} (t)$, showing that they evolve identically, for an initial cross-hair filter state choice $\underline{J} \in \mathfrak {X}$. This correspondence is not trivial. The transition probability $P_{\underline{B}, \, \underline{J}}^{\, \underline{A}, \, \underline{I}} (t)$ admits all intermediate transitions between all joint strength and filter states and, in particular, it allows internal filter transitions without changes in strength state occurring. However, $P_{\underline{B}; \, \underline{J}}^{\, \underline{A}; \, \underline{I}} (t)$ evolves by definition only by changes in strength state. In particular, a synapse in any given strength state cannot change from one cross-hair filter state to another. Such transitions do occur in the full dynamics in $P_{\underline{B}, \, \underline{J}}^{\, \underline{A}, \, \underline{I}} (t)$, but these transitions are already built into the escape densities and hence into the memory kernels in Eq. (74). In the specification of a state $\underline{A}; \, \underline{I}$ or $\underline{B}; \, \underline{J}$ in the transition probability $P_{\underline{B}; \, \underline{J}}^{\, \underline{A}; \, \underline{I}} (t)$, the cross-hair filter states $\underline{I}, \underline{J} \in \mathfrak {X}$ are therefore essentially nothing but non-dynamical labels, or just partitions of any given strength state into substates between which there are no (one-step) transitions. The transition probabilities $P_{\underline{B}; \, \underline{J}}^{\, \underline{A}; \, \underline{I}} (t)$ know nothing about the internal filter dynamics precisely because these dynamics have been integrated out, leaving only the memory kernels. This has motivated our choice of notation $P_{\underline{B}; \, \underline{J}}^{\, \underline{A}; \, \underline{I}} (t)$, so that these additional labels clearly distinguish these transition probabilities from the full processes $P_{\underline{B}, \, \underline{J}}^{\, \underline{A}, \, \underline{I}} (t)$ in which synaptic filters are fully dynamical via their internal transitions within any given strength state.

**Fig. 7:**
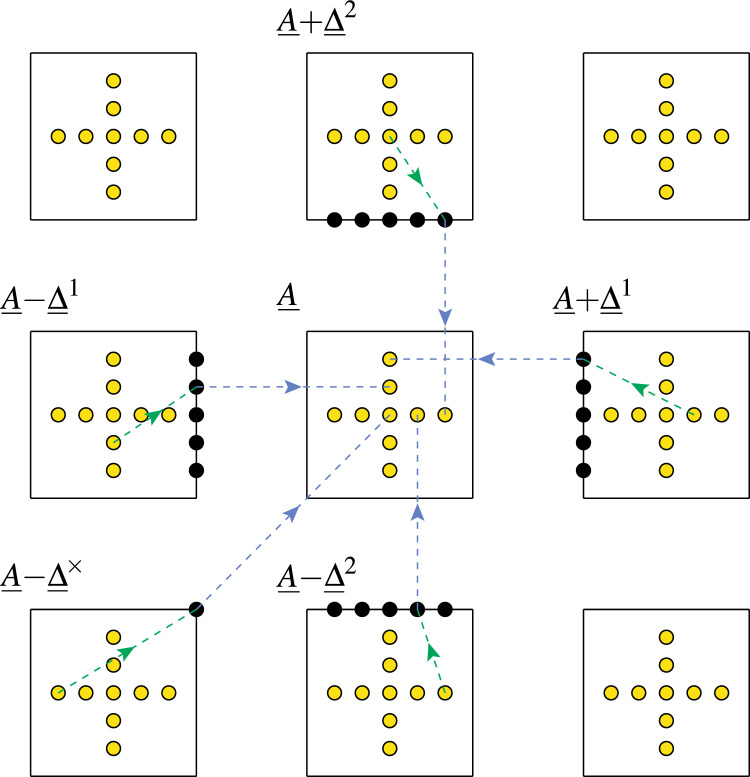
Schematic illustration of the processes by which a pair of synapses change their joint strength states. Each of the nine square blocks defines a particular configuration of synaptic strength states. We show only some of the transitions into the joint strength state $\underline{A}$, corresponding to the central block, from its neighbouring joint strength states. The internal structures of the blocks indicate the cross-hair filter states $\mathfrak {X}$ as cross-shaped arrangements of yellow circles in a manner similar to Fig. 6, for the particular choice of $\varTheta =3$. The black squares delimiting the blocks correspond to the possible filter threshold configurations. The black circles show only those threshold configurations through which the neighbouring blocks’ filter states must escape for the strength state to change to $\underline{A}$. The escape processes lead to the resetting of the filter states back to the relevant cross-hair states. Examples are sketched of possible transitions from the cross-hair filter states of neighbouring strength states to the escape sites (green lines) and then into strength state $\underline{A}$ together with the resetting of the joint filter state back to the appropriate cross-hair state (blue line). The precise details of internal changes indicated by the green lines are not relevant to the overall strength-change dynamics

The move to the forward Chapman–Kolmogorov equation for $P_{\underline{B}; \, \underline{J}}^{\, \underline{A}; \, \underline{I}} (t)$ is now just a trivial matter of pre- rather than post-multiplication by the transition matrix. The resulting master equation for the evolution of the probability of the state $\underline{A}; \, \underline{I}$ follows directly from the forward equation just by dropping reference to the fixed initial state $\underline{B}; \, \underline{J}$. We denote this state probability by $P_{\underline{A}; \, \underline{I}} (t)$. The master equation for $P_{\underline{A}; \, \underline{I}} (t)$ can be written in the compact if slightly cryptic form75To understand the structure of this equation, we refer to Fig. 7, which illustrates the transitions between neighbouring joint strength states. The outer sum over $\underline{J} \in \mathfrak {X}$ in the first line of Eq. (75) sums over all cross-hair filter states of any of the strength states adjacent to $\underline{A}$: any adjacent strength state could be in any cross-hair state. The inner sum restricts to those boundary threshold sites of adjacent strength states through which these adjacent states must escape in order for the joint filter state to be reset to the cross-hair state $\underline{I}$. For a horizontal cross-hair state $\underline{I} = (I_1, 0)$ with $I_1 \ne 0$, the sum inside the integral contains just two contributions from $\underline{\mathfrak {I}} = (I_1, \pm \varTheta )$; similarly for a vertical cross-hair state $\underline{I} = (0, I_2)$ with $I_2 \ne 0$, there are only two contributions from $(\pm \varTheta , I_2)$; for the bull’s eye cross-hair state $\underline{I} = (0, 0)$, there are in general eight contributions from $(0, \pm \varTheta )$, $(\pm \varTheta , 0)$ and $(\pm \varTheta , \pm \varTheta )$ (all four sign combinations for all four corners), although three of these corner states do not contribute due to our particular dynamics. The second line in Eq. (75) is just a sum over the possible escape processes from strength state $\underline{A}$, although these are not shown in Fig. 7. Equation (75) is the general form away from saturated strength states. The equation is modified in the usual way if any strength state is saturated. For example, if $A_1 = 0$, the terms representing potentiation of the non-existent $A_1 = -1$ state are removed and terms representing the impossible depression of the $A_1 = 0$ state are reflected back to the $A_1 = 0$ state, with filter resetting to $I_1 = 0$. Summing the master equation over the cross-hair filter states $\underline{I} \in \mathfrak {X}$, we obtain76where the sum over $\underline{I} \in \mathfrak {X}$ essentially removes the restriction on the $\underline{\mathfrak {I}}$ sum in the first line of Eq. (75) but in the process forces $\underline{J} \rightarrow \underline{I}$ in the outer sum. This is most easily seen just by writing down Eq. (75) for the separate cross-hair cases of $\underline{I} \in \mathfrak {H}$, $\underline{I} \in \mathfrak {V}$ and $\underline{I} \in \mathfrak {Z}$, and then directly summing. Equation (76) describes the evolution of the marginal probability $P_{\underline{A}} (t)$ of each synaptic strength state $\underline{A}$, although the right-hand side does not factorise into separate terms involving only these marginal probabilities because the escape to a different strength state occurs at different rates for different cross-hair states.

The cross-hair $\rightarrow$ cross-hair transitions are fundamental to understanding the change in the joint strength state of two afferents, just as the zero $\rightarrow$ zero transitions are the key processes driving the change in the strength state of a single afferent. Although there are $4 \varTheta - 3$ cross-hair filter states, focusing on the transitions between them with associated changes in synaptic strength affords a huge simplification, both conceptual and computational, compared to having to consider in detail the internal dynamics of the transitions within all $(2 \varTheta - 1)^2$ joint filter states, most of which do not lead directly to changes in synaptic strength. These full dynamics are governed by the renewal equation in Eq. (68), from which the backward Chapman–Kolmogorov equation can be obtained by differentiation, with the equivalent forward equation or the master equation for the probability distribution $P_{\underline{A}, \underline{I}} (t)$ over all joint strength and filter states following directly. This probability distribution $P_{\underline{A}, \underline{I}} (t)$ of course includes all joint filter states in a fully dynamical sense, with the full internal filter dynamics being present that ultimately give rise to the memory kernels $\rho _{\underline{I}; \, \underline{A}}^{\, \underline{\mathfrak {I}}} (t)$ in which these internal dynamics have been integrated out. The distribution $P_{\underline{A}, \underline{I}} (t)$ therefore differs conceptually from $P_{\underline{A}; \, \underline{I}} (t)$: in the former, $\underline{I}$ is a fully dynamical variable, while in the latter, $\underline{I}$ is merely a non-dynamical label distinguishing different cross-hair states. Nevertheless, their marginal strength state distributions are identical because of the established correspondence between $\sum _{\underline{I}} P_{\underline{B}, \, \underline{J}}^{\, \underline{A}, \underline{I}} (t)$ and $\sum _{\underline{I} \in \mathfrak {X}} P_{\underline{B}; \, \underline{J}}^{\, \underline{A}; \underline{I}} (t)$, and so between $\sum _{\underline{I}} P_{\, \underline{A}, \underline{I}} (t)$ and $\sum _{\underline{I} \in \mathfrak {X}} P_{\, \underline{A}; \underline{I}} (t)$. Hence, $\sum _{\underline{I}} P_{\underline{A}, \underline{I}} (t) \equiv \sum _{\underline{I} \in \mathfrak {X}} P_{\underline{A}; \underline{I}} (t)$ for each joint strength state $\underline{A}$, where the evolution of $P_{\underline{A}} (t) = \sum _{\underline{I} \in \mathfrak {X}} P_{\underline{A}; \underline{I}} (t)$ is given by Eq. (76).

### Resetting approximation of Elliott (2011b)

Previously we made the simplifying resetting approximation, so that regardless of how the joint filter state escapes through the boundary $\mathfrak {B}$, both (or, in general, all) filters are reset to their zero states (Elliott 2011b). We used this approximation to generalise the $m = 1$ afferent case in Eq. (49) to the general *m* case in Eq. (56). However, writing down equation Eq. (56) entails a further approximation. The resetting approximation corresponds to the choice  for any $\underline{\mathfrak {I}} \in \mathfrak {B}$ in the absorbing states. With this choice, the fundamental process driving changes in synaptic strength is the single $(0, 0) \rightarrow (0,0)$ transition, rather than all the cross-hair $\rightarrow$ cross-hair transitions. Writing $P_{\underline{A}; \, \underline{0}}(t)$ as the joint strength state probability with this resetting approximation, the master equation in Eq. (75) reduces to77In the first line, the original sum over $\underline{J} \in \mathfrak {X}$ in Eq. (75) collapses to a sum over $\underline{J} \in \mathfrak {Z}$, which just sets $\underline{J} = (0, 0)$. Furthermore, the original sum inside the first integral just becomes a sum over all $\underline{\mathfrak {I}} \in \mathfrak {B}$, because the original restriction  just becomes , which is now always true.

To consider Eq. (77) further, we partition the boundary set $\mathfrak {B}$ into the subsets78corresponding to the four edges of the square excluding the corner states (the arrows point from the inside of the square to the relevant edge) and its top-right corner state, but ignoring the other three, unattainable corner states. Then, for example, for , , and for $\underline{\mathfrak {I}} \in \mathfrak {B}_\uparrow$, . Partitioning the boundary sums in Eq. (77) into these five subsets (ignoring the three irrelevant corners), we can rewrite Eq. (77) in the uncondensed form79 Compared to Eq. (56) for $m = 2$ afferents, Eq. (79) contains the additional, final term in which both afferents change strength simultaneously. The absence of such a term from Eq. (56) is hardly surprising, and this contribution to Eq. (79) would be absent were we to set $r^{\, \ddagger }_{\underline{B}} (\underline{\lambda }) \equiv 0$. However, even in suppressing these diagonal joint filter transitions, Eq. (56) contains only the one-dimensional filter escape processes $\rho _{0; \, \underline{B}}^{\pm \varTheta } (\lambda _i; t)$, while Eq. (79) contains sums $\sum \rho _{\underline{0}; \, \underline{B}}^{\underline{\mathfrak {I}}} (t)$ over the relevant escape edges of the joint filter state space to give the required strength-change transitions. These latter are certainly not just the one-dimensional escape processes. Thus, not only must we make the resetting approximation to reduce Eq. (75) to Eq. (56), but we must also replace the full two-dimensional random walk dynamics in joint filter space by two separate, one-dimensional random walks in each afferent’s own filter state. This last approximation was unrecognised in Elliott (2011b). We will compare all three models below, referring to them as the “full” model in which all cross-hair states are present; the “resetting” model in which both (or all) filters are reset to zero when any one of them reaches threshold but the sub-threshold filter dynamics are considered fully; and the “2011” model in which both (or all) filters are reset to zero at any threshold and the full sub-threshold filter dynamics are replaced by separate, one-dimensional processes.

### Equilibrium strength distribution and lifetimes of segregated states

As discussed for the single afferent case, we are mainly concerned with the large *t* or equilibrium behaviour of $P_{\underline{A}; \, \underline{I}} (t)$, corresponding to the outcome of neuronal development. The equilibrium distribution $P_{\underline{A}; \, \underline{I}} (\infty )$ follows at once from Eq. (75) by again taking the Laplace transform and using the normal Tauberian theorem. The equilibrium distribution satisfies80with the usual replacements to accommodate strength saturation, and with the asymptotic escape rates $r_{\underline{I}; \, \underline{A}}^{\, \underline{\mathfrak {I}}} (\infty )$ computed via Eq. (67). Taking $\underline{I} = (I_1, 0) \in \mathfrak {H}$ (with $I_1 \ne 0$), we can write Eq. (80) out in full without the  and  operators in the form

81 $$\begin{aligned}&\Big [ \sum _{\underline{\mathfrak {I}} \in \mathfrak {B}} r_{(I_1, 0); \, \underline{A}}^{\, \underline{\mathfrak {I}}} (\infty ) \Big ] \, P_{\underline{A}; \, (I_1, 0)} (\infty ) \nonumber \\&\quad = \sum _{\underline{J} \in \mathfrak {X}} \Big [ r_{\underline{J}; \, \underline{A} - \underline{\varDelta }^2}^{\, (I_1, +\varTheta )} (\infty ) \, P_{\underline{A} - \underline{\varDelta }^2; \, \underline{J}} (\infty ) \nonumber \\ &\qquad + r_{\underline{J}; \, \underline{A} + \underline{\varDelta }^2}^{\, (I_1, -\varTheta )} (\infty ) \, P_{\underline{A} + \underline{\varDelta }^2; \, \underline{J}} (\infty ) \Big ]. \end{aligned}$$

For $\underline{I} = (0, I_2) \in \mathfrak {V}$ (with $I_2 \ne 0$), we have a very similar equation involving the two escape sites $(\pm \varTheta , I_2)$ and adjacent strength states $\underline{A} \mp \underline{\varDelta }^1$. For the bull’s eye state $\underline{I} = (0, 0) \in \mathfrak {Z}$, the right-hand side contains five contributions from the escape sites $(0, \pm \varTheta )$, $(\pm \varTheta , 0)$ and $(+ \varTheta , + \varTheta )$ from the associated adjacent strength states $\underline{A} \mp \underline{\varDelta }^2$, $\underline{A} \mp \underline{\varDelta }^1$ and $\underline{A} - \underline{\varDelta }^{\! \times \!}$, respectively. Unlike the one-dimensional case for which an analytical solution of the equilibrium distribution is available, we must numerically solve Eq. (80) as a linear system in $(N+1)^2 (4 \varTheta - 3)$ variables, although various symmetries may be used to reduce the total number of degrees of freedom.

Similarly to the one-dimensional case considered in Sect. 2.5, for *T* below some critical plasticity step size $T_{\textrm{C}}$, the two-dimensional marginal equilibrium strength state distribution $P_{\underline{A}} (\infty )$ exhibits two maxima at $\underline{A} = (0, A)$ and $\underline{A} = (A, 0)$ for $A > 0$. These maxima correspond to segregated states in which one or the other afferent exclusively controls the target cell upon the completion of neuronal development. Any particular realisation of the dynamics will therefore tend to occupy, or be close to, one of these two segregated, macroscopic states, with fluctuations driving the system between them. We can quantify the stability of these segregated states by determining the MFPT for the system to undergo a transition from one segregated state to the other (Elliott, 2011b; see van Kampen, 1992 for a general discussion of bistable systems). As these dynamics occur over long time scales, it suffices to replace $\rho _{\underline{I}; \, \underline{A}}^{\underline{\mathfrak {I}}} (t)$ in Eq. (75) with the asymptotic form $r_{\underline{I}; \, \underline{A}}^{\, \underline{\mathfrak {I}}} (\infty ) \delta (t)$, which collapses the convolution integrals. To calculate the lifetime of segregated states, we move to the continuum limit by regarding *T* as a small expansion parameter, so that the master equation in Eq. (75) can be expanded in small *T* to obtain a Fokker–Planck equation (van Kampen 1992). To expand in *T*, we write $P_{\underline{A}; \, \underline{I}} (t) \rightarrow P_{\, \underline{I}} (\underline{S}; t)$ and $r_{\underline{I}; \, \underline{A}}^{\, \underline{\mathfrak {I}}} (\infty ) \rightarrow r_{\underline{I}}^{\, \underline{\mathfrak {I}}} (\underline{S}; \infty )$, where $\underline{S} = T \underline{A}$ is a vector of synaptic strengths rather than synaptic strength states. Then, the master equation contains terms such as , and since82Equation (75) becomes83where the truncated differential operator $\mathscr {D}_{\underline{\mathfrak {I}}}$ is

84 $$\begin{aligned} \mathscr {D}_{\underline{\mathfrak {I}}}&= 1 - T \sum _{i=1}^m \textrm{sgn}( \mathfrak {I}_i ) \, \delta _{\, | \mathfrak {I}_i | , \varTheta } \; \frac{\partial }{\partial S_i} \nonumber \\&\quad + \frac{1}{2} \, T^2 \! \! \sum _{i,j=1}^m \textrm{sgn}( \mathfrak {I}_i ) \, \delta _{\, | \mathfrak {I}_i | , \varTheta } \; \textrm{sgn}( \mathfrak {I}_j ) \, \delta _{\, | \mathfrak {I}_j | , \varTheta } \, \frac{\partial ^2}{\partial S_i \partial S_j}. \end{aligned}$$

In general, Eq. (83) is not a standard Fokker–Planck equation because it contains non-derivative terms on the right-hand side. These terms cancel only when we sum over the cross-hair filter states, but similarly to Eq. (76), while the left-hand side would then involve only the marginal distribution $P (\underline{S}; t) = \sum _{\underline{I} \in \mathfrak {X}} P_{\, \underline{I}} (\underline{S}; t)$, the right-hand side would not factorise into terms involving only this marginal distribution. The presence of the non-derivative terms on the right-hand side of Eq. (83) reflects the impossibility of expressing the synaptic dynamics of two or more afferents purely in terms of changes in their synaptic strengths, because the cross-hair filter states cannot be eliminated at the level of the macroscopic dynamics. Even for a single afferent, its zero filter state also cannot be eliminated from the dynamics, although in this case $P_{A; \, 0} (t) \rightarrow P_{\, 0} (S; t)$ corresponds to the marginal distribution *P*(*S*; *t*) and so Eq. (83) collapses to a standard Fokker–Planck equation for a single afferent. This collapse also occurs for the resetting and 2011 models, because again the distribution $P_{\underline{A}; \underline{0}} (t) \rightarrow P_{\, \underline{0}} (\underline{S}; t)$ also just corresponds to the marginal distribution $P (\underline{S}; t)$.

To compute the lifetimes of segregated states, we are therefore forced to use the resetting or 2011 models as approximations. However, since the resetting model in Eq. (79) involves the full two-dimensional escape rates, which can only be obtained numerically, we must resort to using the 2011 model, for which analytical results are available (see Elliott 2011b, for details). Writing $S_\pm = S_1 \pm S_2$, the key competitive dynamics are in the $S_-$ variable. For small enough $\varsigma / \mu$, $S_+$ rapidly evolves to a fixed value, call it $\mathscr {S}_+$, and so we need consider the Fokker–Planck equation only in the $S_-$ variable,

85 $$\begin{aligned} \frac{\partial P(S_-; t)}{\partial t} = \frac{\partial }{\partial S_-} \left[ U' (S_-) P(S_-; t) \right] + \vartheta \frac{\partial ^2}{\partial S_-^2} P(S_-; t), \end{aligned}$$

where $U' (S_-) = \textrm{d} U (S_-) / \textrm{d} S_-$, with the potential $U(S_-)$ being given by

86 $$\begin{aligned} U (S_-) = - \frac{1}{2} \varLambda \, S_-^2. \end{aligned}$$

The potential is calculated to second order in $\varsigma / \mu$ and the diffusion constant to zeroth order. We have

87a $$\begin{aligned} \varLambda&= (1 - \mathfrak {r}) \, \varsigma ^2 \, \frac{T}{\varTheta } \, \frac{\partial ^2}{\partial x \partial y} \left[ r^+ (x, y) - r^- (x, y) \right] \big |_{x = \mu , y = \mu \mathscr {S}_+}\nonumber \\&= (1 - \mathfrak {r}) \, \varsigma ^2 \, \frac{T}{\varTheta } \, \frac{(\lambda _+ - \lambda _-) (\lambda _- + 2 \mu )(2 \lambda _- + 2 \mu - \lambda _+)}{(2 \lambda _- + 3 \mu - \lambda _+)^2 (\lambda _+ + \mu )^2}, \end{aligned}$$

87b $$\begin{aligned} \vartheta&= \left( \frac{T}{\varTheta } \right) ^2 \left[ r^+ (\mu , \mu \mathscr {S}_+) + r^- (\mu , \mu \mathscr {S}_+) \right] \nonumber \\&= 2 \left( \frac{T}{\varTheta } \right) ^{\! \! \! 2} \, \frac{\mu ( \lambda _- + \mu - \lambda _+)}{2 \lambda _- + 3 \mu - \lambda _+}, \end{aligned}$$

where we have written the asymptotic induction rates $r^\pm _B$ in Eq. (40) as $r^\pm ( \lambda _\pi , \lambda _p )$ to indicate their dependence on the pre- and postsynaptic firing rates. The Fokker–Planck equation in Eq. (85) describes the deterministic dynamics of the $S_-$ variable in the inverted ($\varLambda > 0$) quadratic potential $U(S_-)$, but subject to fluctuations controlled by the parameter $\vartheta$. The variable $S_-$ takes values in the interval $[ - \mathscr {S}_+, + \mathscr {S}_+ ]$, enforced by the reflecting strength state boundary conditions, with $\mathscr {S}_+ = ( \lambda _- + \mu - \lambda _+ ) / \mu$ when evaluated to order $(\varsigma / \mu )^0$. We require $\mathscr {S}_+ > 0$ for the existence of segregated states, and when this condition is satisfied, $\vartheta > 0$, guaranteeing that the diffusion constant in Eq. (85) is positive. The calculation of the MFPT $\tau _{\textrm{trans}}$ for a transition between the segregated states is standard (see van Kampen 1992, §XIII.2 for a general discussion), and we obtain (Elliott 2011b, Eq. 4.31, in which $\frac{1}{2} \sigma ^2$ is $\vartheta$ in our present notation)

88 $$\begin{aligned} \tau _{\textrm{trans}} = \frac{\pi }{\varLambda } \, \textrm{erf} \left( \! \! \! \sqrt{\frac{\varDelta U}{\vartheta } } \right) \textrm{erfi} \left( \! \! \! \sqrt{\frac{\varDelta U}{\vartheta }} \right) , \end{aligned}$$

where $\varDelta U = \frac{1}{2} \varLambda \, \mathscr {S}_+^2$ is the potential barrier over which the system must climb to undergo a transition, and erf and erfi are the standard and imaginary error functions, respectively. Writing the overall factor $\pi / \varLambda$ as $\alpha \, \varTheta /T$ and the argument of the two error functions as $\sqrt{\beta \, \varTheta /T}$, the small and large $T / \varTheta$ behaviours of $\tau _{\textrm{trans}}$ are given by

89 $$\begin{aligned} \tau _{\textrm{trans}} \sim {\left\{ \begin{array}{ll} \frac{\alpha }{\sqrt{\pi \, \beta }} \, \sqrt{\frac{\varTheta }{T}} \, \exp \left( \beta \frac{\varTheta }{T} \right) & \text{ for } \text{ small } T / \varTheta \\ \frac{4 \alpha \beta }{\pi } \left( \frac{\varTheta }{T} \right) ^2 & \text{ for } \text{ large } T / \varTheta \end{array}\right. }. \end{aligned}$$

As $T / \varTheta$ decreases, the stability of segregated states becomes vastly enhanced, growing essentially exponentially fast with $\varTheta / T$. For small enough $T / \varTheta$, segregated states are therefore for all practical purposes infinitely long-lived (i.e. vastly longer than the age of the universe, let alone the lifespan of a mammal).

**Fig. 8:**
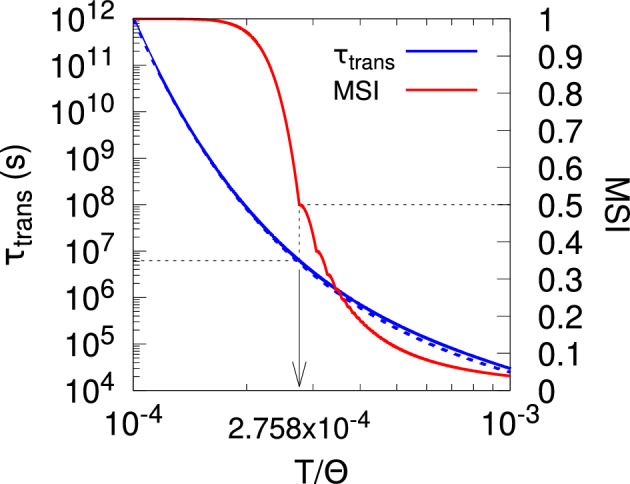
Mean transition time $\tau _{\textrm{trans}}$ between two segregated states and the corresponding mean segregation index $\langle | \langle S_I \rangle | \rangle$, as functions of $T / \varTheta$. The MFPT $\tau _{\textrm{trans}}$ is obtained from Eq. (88) while the corresponding MSI is estimated from the simple model discussed in the main text for a probe interval of $\tau _{\textrm{probe}} = 1.25 \times 10^7$ s. The dashed blue line shows the small $T/\varTheta$ limit of $\tau _{\textrm{trans}}$ in Eq. (89), indicating that over the displayed range of $T / \varTheta$, $\tau _{\textrm{trans}}$ is indistinguishable from, or very close to, its exponential-like form in Eq. (89). At the indicated value of $T / \varTheta \approx 2.758 \times 10^{-4}$, MSI = $\frac{1}{2}$ and $\tau _{\textrm{trans}} = 6.25 \times 10^6~\textrm{s} = \frac{1}{2} \tau _{\textrm{probe}}$. All other parameters take the standard values used in Fig. 3, i.e. $\mu = 50$ Hz, $\varsigma / \mu = 1/4$, $\lambda _- = 50$ Hz (or $\tau _- = 20$ ms), $\lambda _+ = \lambda _- / \rho$ where $\rho = 3/5$, and $\lambda _{\textrm{spont}} = 0.1$ Hz; we have additionally set the correlation coefficient $\mathfrak {r}$ between the two afferents’ activities to zero

In simulation, it is impractical to measure $\tau _{\textrm{trans}}$ when it is very large. To gauge the stability of segregated states in simulation, we therefore employ the segregation index, $S_I = S_- / S_+$. We first allow sufficient time (corresponding to $1.25 \times 10^7$ firing epochs of duration 1000 ms) for segregated states to emerge in a simulation, and then we employ a probe interval $\tau _{\textrm{probe}} = 1.25 \times 10^7$ s (the same number of firing epochs) during which we average $S_I$ over its value at the end of each firing epoch within this probe period, resulting in $\langle S_I \rangle$. If $\tau _{\textrm{trans}} \gg \tau _{\textrm{probe}}$, then $\langle S_I \rangle$ will be very close to $+1$ or $-1$, but if $\tau _{\textrm{trans}} \ll \tau _{\textrm{probe}}$, then $\langle S_I \rangle$ will be very close to zero. We then average $| \langle S_I \rangle |$ over multiple simulations, giving the mean segregation index (MSI) $\langle | \langle S_I \rangle | \rangle$, where the “inner” average refers to the probe interval and the “outer” average refers to multiple simulations. We average over $10^3$ simulations to obtain good statistics. Given a value of $\tau _{\textrm{trans}}$ from Eq. (88), we can estimate the corresponding MSI obtained in simulation by considering a simple model of the transition of the system between the two segregated states. In particular, consider a sequence of periods of length $\tau _{\textrm{trans}}$ during each of which $S_I = \pm 1$ with probability $\frac{1}{2}$. Suppose the probe interval starts at a time $\tau$ into one of these periods. The time remaining from this first period is

90 $$\begin{aligned} \tau _1 (\tau ) = \min \{ \tau _{\textrm{probe}}, \tau _{\textrm{trans}} - \tau \}. \end{aligned}$$

The number of full periods within the probe interval is

91 $$\begin{aligned} N(\tau ) = \left\lfloor \frac{\tau _{\textrm{probe}} - \tau _1 (\tau )}{\tau _{\textrm{trans}}} \right\rfloor , \end{aligned}$$

where $\lfloor \cdot \rfloor$ denotes the floor function. The time remaining at the end of these $N(\tau )$ full periods is

92 $$\begin{aligned} \tau _2 (\tau ) = \tau _{\textrm{probe}} - \tau _1 (\tau ) - N(\tau ) \, \tau _{\textrm{trans}}. \end{aligned}$$

If $N(\tau )$ decomposes into $N_+$ periods with $S_I = +1$ and $N(\tau ) - N_+$ periods with $S_I = -1$, then the absolute value of the segregation index from these $N(\tau )$ full and two partial periods is

93 $$\begin{aligned} | \langle S_I \rangle | = \frac{\left| \pm \, \tau _1 (\tau ) + [2 N_+ - N(\tau )] \, \tau _{\textrm{trans}} \pm \tau _2 (\tau ) \right| }{\tau _{\textrm{probe}}} \end{aligned}$$

with probability ${^{N (\tau )}}C_{N_+} / 2^{N (\tau ) + 2}$ (all four sign combinations). After simplification, the MSI from this simple model is:

94 $$\begin{aligned} \langle | \langle S_I \rangle | \rangle&= \frac{1}{\tau _{\textrm{trans}}} \int _0^{\tau _{\textrm{trans}}} \! \! \textrm{d} \tau \sum _{N_+ = 0}^{N (\tau )} \frac{^{N (\tau )}C_{N_+}}{2^{N (\tau )}} \nonumber \\&\quad \times \left( \frac{| 2 N_+ \tau _{\textrm{trans}} - \tau _{\textrm{probe}} | + | 2 N_+ \tau _{\textrm{trans}} - \tau _{\textrm{probe}} + 2 \tau _1 (\tau ) |}{2 \tau _{\textrm{probe}}} \right) . \end{aligned}$$

By considering $n \, \tau _{\textrm{trans}} \le \tau _{\textrm{probe}} \le (n+1) \tau _{\textrm{trans}}$ for integer $n \ge 0$, we can explicitly evaluate $\langle | \langle S_I \rangle | \rangle$. Writing $S_I (n)$ for any given value of *n*, we find that $\langle | \langle S_I (2n+1) \rangle | \rangle = \langle | \langle S_I (2n) \rangle | \rangle$, with the even values being given by

95 $$\begin{aligned} \langle | \langle S_I (2n) \rangle | \rangle = \frac{{^{2n}}C_n}{2^{2n}} \left[ (n+1) - \frac{\tau _{\textrm{probe}}}{4 \tau _{\textrm{stab}}} - n^2 \frac{\tau _{\textrm{stab}}}{\tau _{\textrm{probe}}} \right] . \end{aligned}$$

We may replace *n* by $\left\lfloor \tau _{\textrm{probe}} / (2 \tau _{\textrm{stab}}) \right\rfloor$ on the right-hand side of Eq. (95) to obtain a general expression for $\langle | \langle S_I \rangle | \rangle$ without specifying the value of *n*. For $\tau _{\textrm{probe}} \le 2 \tau _{\textrm{trans}}$ we have

96 $$\begin{aligned} \langle | \langle S_I \rangle | \rangle = 1 - \frac{\tau _{\textrm{probe}}}{4 \tau _{\textrm{trans}}}, \end{aligned}$$

so that as $\tau _{\textrm{trans}} \rightarrow \infty$ for a fixed probe interval $\tau _{\textrm{probe}}$, $\langle | \langle S_I \rangle | \rangle \rightarrow 1$ as required, and when $\tau _{\textrm{probe}} = 2 \tau _{\textrm{trans}}$, $\langle | \langle S_I \rangle | \rangle = \frac{1}{2}$. In Fig. 8, we plot both $\tau _{\textrm{trans}}$ from Eq. (88) and the corresponding value of the MSI according to this simple model, indicating how the latter saturates at its extremes as $T / \varTheta$ increases or decreases. We will compare this model of the MSI to the MSI obtained from simulation in the next section.

## Numerical and simulation results

We now turn to a discussion of our numerical results for the full model with two afferents, comparing them to the results for the 2011 and resetting models, and also where appropriate to the results for the one-afferent case. Numerical solutions of linear systems are obtained by standard numerical methods. Some of these solutions may be obtained on standard desktop or laptop computers, but larger systems require very high memory (0.25 to 0.5 TB). We also compare our numerical results to MSI results obtained from simulation. Simulations are run according to protocols discussed above and extensively elsewhere (Appleby and Elliott 2006; Elliott and Lagogiannis 2009; Elliott 2011b). In summary, at the start of a simulation, each synapse’s strength state is randomly set so that all synapses are initially of similar strength. For convenience, their separate, independent STDP and filter states are set to OFF and zero, respectively; we could instead randomly draw them from a distribution such as their equilibrium distribution, but their initial states are unimportant for the long-term behaviour. At the start of each firing epoch, an afferent’s firing rate is randomly set to $\mu \pm \varsigma$ with equal probability (with zero correlation between different afferents), and the postsynaptic firing rate is obtained. These pre- and postsynaptic firing rates determine the times of the pre- and postsynaptic Poisson spikes during the current firing epoch. For efficiency, we employ an event-driven approach within each epoch, so that the simulation steps between temporally consecutive events rather than uses small, discrete time steps. Events are pre- and postsynaptic spikes, or the STDP decay processes that arise at each synapse if an appropriate spike does not arrive first. As each event occurs, the corresponding synapse’s STDP state is updated accordingly, and its filter state is updated if an induction signal is generated. If a filter threshold occurs, the synapse’s strength state is updated; this will not affect the postsynaptic firing rate until the next firing epoch. Simulations are run as described earlier over multiple epochs, including the probe period used to determine the stability of developed patterns of connectivity. Data are averaged over many simulations for good statistics.

### Equilibrium distribution of full model

**Fig. 9:**
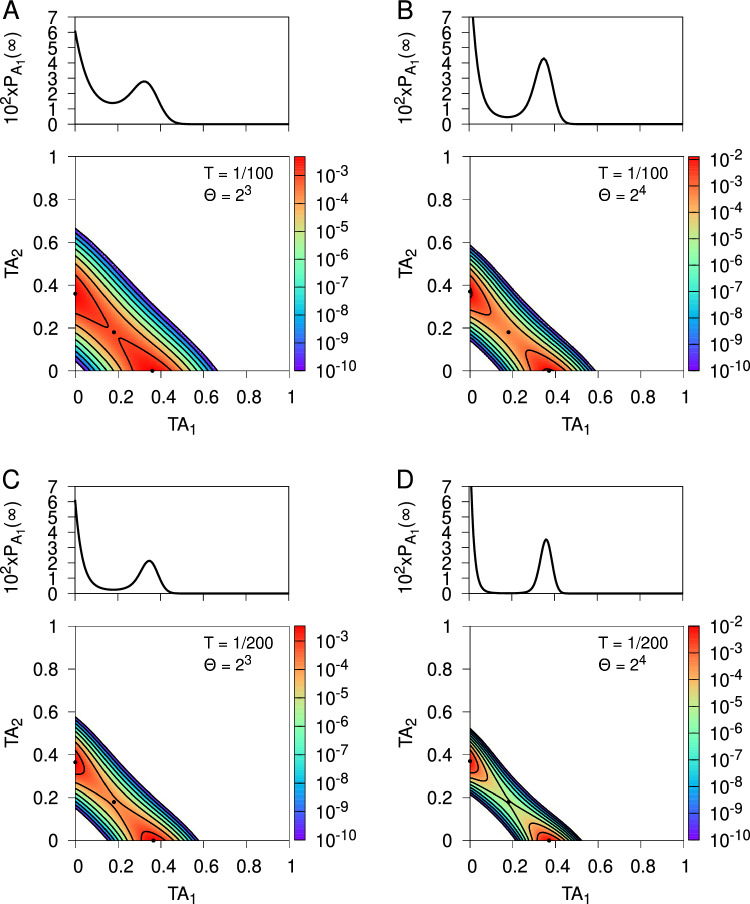
Equilibrium probability distribution of synaptic strengths for two afferents synapsing on a target cell. Each panel shows both the marginal probability distribution $P_{A_1}(\infty ) = \sum _{A_2} P_{(A_1,A_2)} (\infty )$ for one afferent (upper graph) and the full two-dimensional probability distribution $P_{(A_1,A_2)} (\infty )$ for both afferents as a combined heat and contour map (lower graph). The three small black blobs on each heat map indicate the two maxima of the probability distribution (on the two axes) and the saddle point equidistant between them. The two contours for $10^{-2}$ in the contour map in panel D are very close to the axes and are obscured by the black blobs. The choices of plasticity step size *T* and filter threshold $\varTheta$ are indicated in each panel. All other parameters take their standard values from Fig. 8

In Fig. 9, we plot illustrative examples of the equilibrium probability distribution $P_{\underline{A}} (\infty )$ of two afferents’ synaptic strengths in the full model for various choices of synaptic plasticity step size *T* and filter threshold $\varTheta$ that ensure the presence of segregated states. We show combined heat and contour maps of the two-dimensional equilibrium probability distribution as well as the marginal equilibrium distribution for just one afferent’s strength (by symmetry both afferents have the same marginal distributions). Very small probabilities ($< 10^{-10}$) are suppressed in the two-dimensional distributions to avoid saturating the heat maps. The two-dimensional distributions have maxima at (*A*, 0) and (0, *A*) for some synaptic strength state $A > 0$. These macroscopic states correspond to segregated states of synaptic connectivity in which one or the other afferent wins complete control of the target cell. Both segregated states are equiprobable by symmetry. These two states are connected by a saddle point at (*B*, *B*) for some other synaptic strength state $B > 0$. At (*B*, *B*), the probability distribution is a maximum along the line $A_1 = A_2$, but a minimum in the locally orthogonal direction. For the choice of parameters used in this figure, and specifically for the choice of correlation coefficient $\mathfrak {r} = 0$, the two maxima and the saddle lie very nearly on the straight line $A_1 + A_2 = A$, so that $B \approx A/2$. Only by significantly anti-correlating the two afferents’ activities would these three points lie on a curve that is not nearly linear. Fluctuations drive any particular realisation of the system between the two segregated states along a path typically very close to the saddle point, with trajectories deviating from the path between these three extrema being suppressed. As $\varTheta$ increases or *T* decreases, we see that the equilibrium probability distribution concentrates around the two maxima with the saddle decreasing in probability. For decreasing $T / \varTheta$, fluctuations that drive transitions between the segregated states therefore develop over increasingly long time scales, so that the quasi-stable segregated states become increasingly long-lived. These qualitative features of the equilibrium probability distributions agree with the quantitative dependence of the mean transition time $\tau _{\textrm{trans}}$ on $T / \varTheta$ in Eq. (89).

**Fig. 10:**
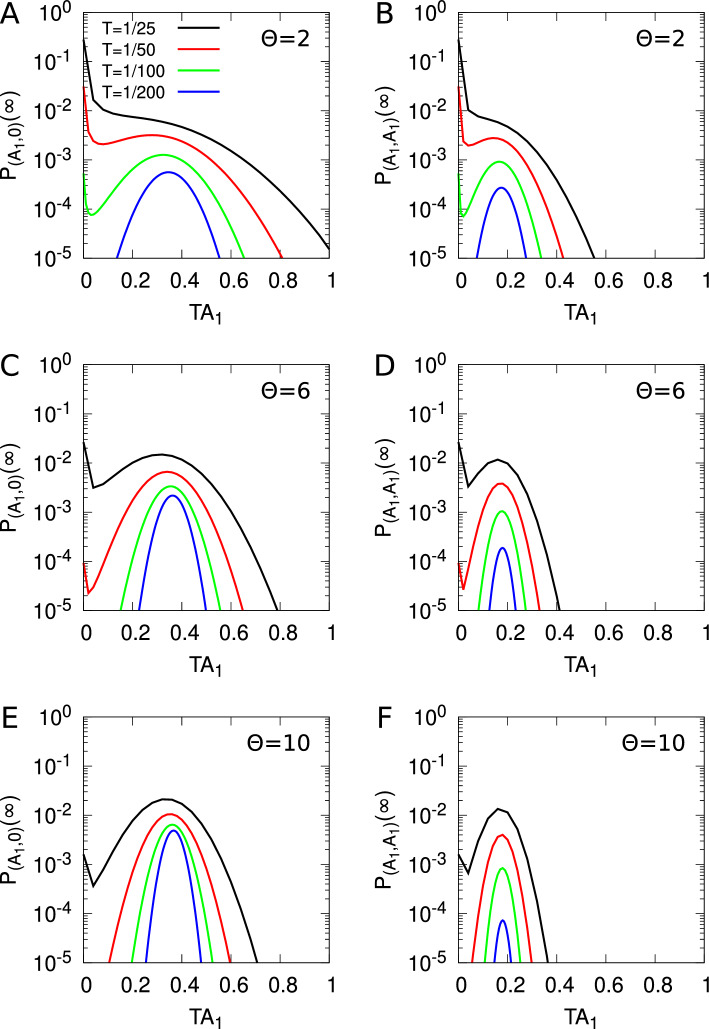
Slices through the equilibrium probability distribution of synaptic strengths for two afferents synapsing on a target cell. Panels A, C and E show the slice $P_{(A_1,0)} (\infty )$ corresponding to the second afferent being in the zero strength state. Panels B, D and F show the diagonal slice $P_{(A_1, \, A_1)} (\infty )$ along which both afferents are in the same strength state; the saddle point connecting the two maxima lies on this slice. The plasticity step size *T* is indicated in the common legend in panel A, with the filter threshold $\varTheta$ shown in each panel. All other parameters are as in Fig. 8

Although the entire two-dimensional equilibrium distribution $P_{(A_1, A_2)} (\infty )$ contains all information about the equilibrium structure of two afferents’ synaptic strengths, the two slices $P_{(A_1,0)} (\infty )$ and $P_{(A_1, A_1)} (\infty )$ through this distribution more compactly illustrate the central features in relation to the presence (or otherwise) of segregated states and the saddle point between them. The slice $P_{(A_1,0)} (\infty )$ is preferred over the marginal $P_{A_1} (\infty ) = \sum _{A_2} P_{(A_1,A_2)} (\infty )$ because the former is better at illustrating the onset of a non-zero maximum away from the origin as $T / \varTheta$ decreases and thus when a bifurcation occurs in the structure of the two-dimensional distribution. In Fig. 10, we therefore show these slices for different choices of *T* and $\varTheta$. Similarly to the results for one afferent shown in Fig. 3, for fixed $\varTheta$ as *T* decreases we again see the onset of a maximum in the slice $P_{(A_1,0)} (\infty )$ for $A_1 > 0$, with the maximum becoming more pronounced and sharper as *T* reduces below its critical value $T_{\textrm{C}}$. The same is true for the slice $P_{(A_1, A_1)} (\infty )$. Comparing the maximum values of $P_{(A_1,0)} (\infty )$ and $P_{(A_1, A_1)} (\infty )$, for $\varTheta = 2$ they are rather similar (at least for the displayed values of *T* in Fig. 10), but as $\varTheta$ increases, the saddle’s amplitude becomes suppressed compared to the segregated states. For $\varTheta = 6$ (panels C and D) and $\varTheta = 10$ (panels E and F), we also clearly see that although the amplitude of a segregated maximum falls only somewhat as *T* decreases, the amplitude of the saddle falls dramatically as *T* decreases. Again, these qualitative features accord with the dependence of $\tau _{\textrm{trans}}$ on $T / \varTheta$. Thus, $\tau _{\textrm{trans}}$ grows essentially exponentially in $\varTheta / T$ because of this differential suppression of the saddle’s amplitude relative to the segregated states as $\varTheta$ increases or *T* decreases.

It is noteworthy that the results for $P_{(A_1,0)} (\infty )$ for $\varTheta = 2$ in Fig. 10A for two afferents are qualitatively quite similar to those shown for $P_A (\infty )$ also for $\varTheta = 2$ in Fig. 3B for just one afferent. The principal difference is merely one of normalisation: were we instead to plot not $P_{(A_1,0)} (\infty )$ but the renormalised slice $P_{(A_1,0)} (\infty ) / \sum _{A_1} P_{(A_1,0)} (\infty )$ in Fig. 10A, the scales of the ordinates would also be very similar. This similarity between the one- and two-afferent cases holds for general $\varTheta$. This observation suggests that the structure of the equilibrium distribution of two (and in general more than one) afferents’ synaptic strengths is tightly constrained by the underlying structure that is inherited from the one-afferent case. Of course, there cannot by definition be segregated states for one afferent, but there can be, and are, maxima of the equilibrium probability distribution away from the origin. The existence of such maxima is a necessary if not sufficient condition for the presence of segregated states. If the equilibrium strength distribution of two (or more) afferents is indeed largely constrained by the one-afferent case, then this would suggest that the numerical results for the full, resetting and 2011 models for two afferents should be similar, and similar where appropriate to those for just one afferent. We now turn to this comparison.

### Comparison between models

**Fig. 11:**
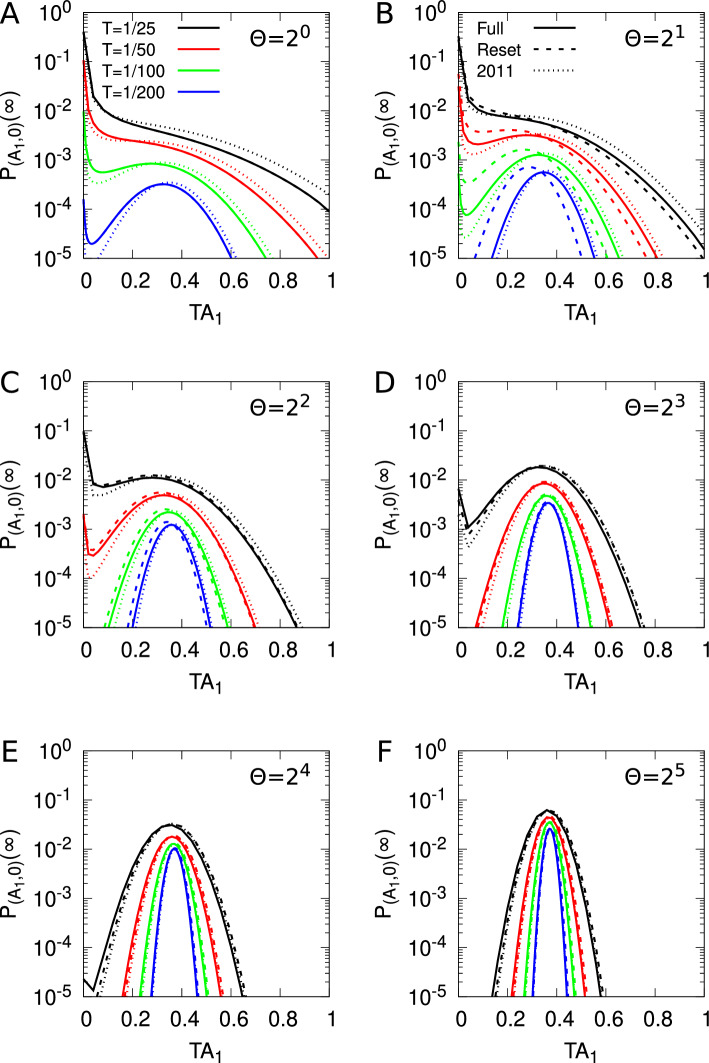
Comparison of the equilibrium probability distribution of synaptic strengths in the full model and its two approximated forms of the resetting and 2011 models. For clarity only the slice $P_{(A_1,0)} (\infty )$ is shown. Different line styles are used for each model as indicated in the common legend in panel B; different line colours distinguish the choice of plasticity step size *T* as indicated in the common legend in panel A. The choice of filter threshold $\varTheta$ is displayed in each panel. For $\varTheta = 2^0$ in panel A, the results for the full and resetting models are identical and so indistinguishable. Other parameters are the same as in Fig. 8

In Fig. 11, we plot the slice $P_{(A_1,0)} (\infty )$ for the full, resetting and 2011 models for different choices of $\varTheta$ and *T*. Results for the other slice $P_{(A_1, A_1)} (\infty )$ exhibit the same overall features and so are not shown. For all displayed choices of *T* and $\varTheta$, the results for all models are qualitatively very similar. For smaller choices of $\varTheta > 1$ and for smaller *T*, the resetting model exhibits a greater deviation from the full model than the 2011 model; for larger *T*, this trend is reversed. (For $\varTheta = 1$ the full and resetting models are identical because of the memorylessness of exponential distributions; the 2011 model does not reduce to the full model for $\varTheta = 1$ because it lacks the doubly potentiating induction process $r_{\underline{B}}^{\, \ddagger } (\underline{\lambda })$.) As $\varTheta$ increases, all three models’ results become not only qualitatively but also quantitatively very similar. These three models are dynamically different, so we would not expect convergence of their equilibrium distributions, but overall their distributions do become closer as $\varTheta$ increases. We see that the resetting and 2011 models’ results approach each other more closely than they approach the full model as $\varTheta$ increases, but all three models’ results become quantitatively close.

**Fig. 12:**
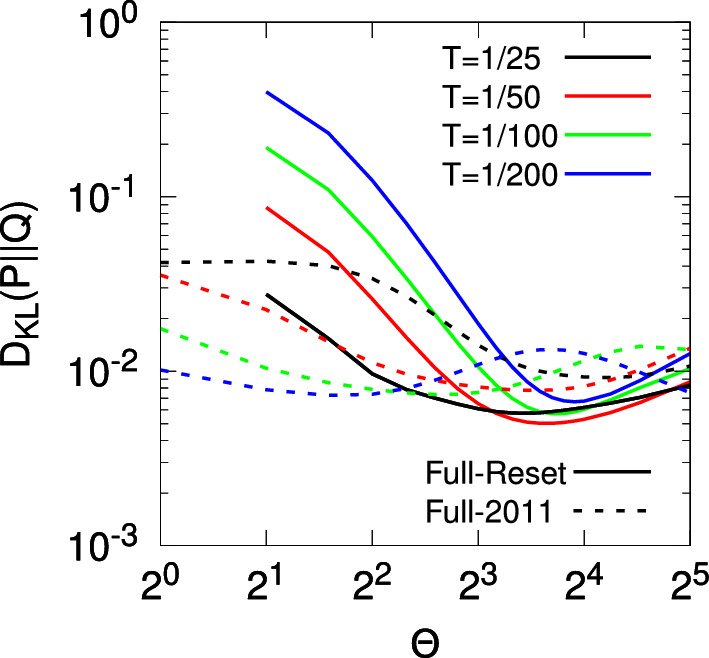
Kullback–Leibler divergence of the equilibrium probability distribution of synaptic strengths between the full model and either the resetting model or the 2011 model, as a function of filter threshold $\varTheta$. The Kullback–Leibler divergence between the full and resetting models for $\varTheta = 1$ is exactly zero as the two distributions are always identical in this case. Standard parameters are as in Fig. 8

To quantify the closeness or similarity of probability distributions, we use the Kullback–Leibler (KL) divergence. Figure 12 plots the KL divergence between the two-dimensional equilibrium probability distributions of the full and resetting models and between those of the full and 2011 models as a function of $\varTheta$ for different choices of *T*. Consistent with the behaviour exhibited by the one-dimensional slices $P_{(A_1, 0)} (\infty )$ in Fig. 11, for smaller $\varTheta$ ($\varTheta > 1$), the KL divergence between the full and resetting models’ two-dimensional distributions is larger than that between the full and 2011 distributions for smaller *T*, but smaller for larger *T*. For larger $\varTheta$, both KL divergences hover around $10^{-2}$, indicating that all three distributions are very similar. We are not able to obtain numerical solutions for the full and resetting models for $\varTheta$ in excess of around 32 and for smaller *T* because of the computational time and memory constraints involved even in a cluster environment,^3^ so we cannot extend Fig. 12 out to larger values of $\varTheta$. As discussed, all three models are dynamically different, so we should not expect both KL divergences to approach zero as $\varTheta$ increases further. Rather, if the trends exhibited in Fig. 11 continue for larger $\varTheta$, then we might expect the two KL divergences roughly speaking to stabilise at some low value, reflecting closeness but not exact identity of distributions.

**Fig. 13:**
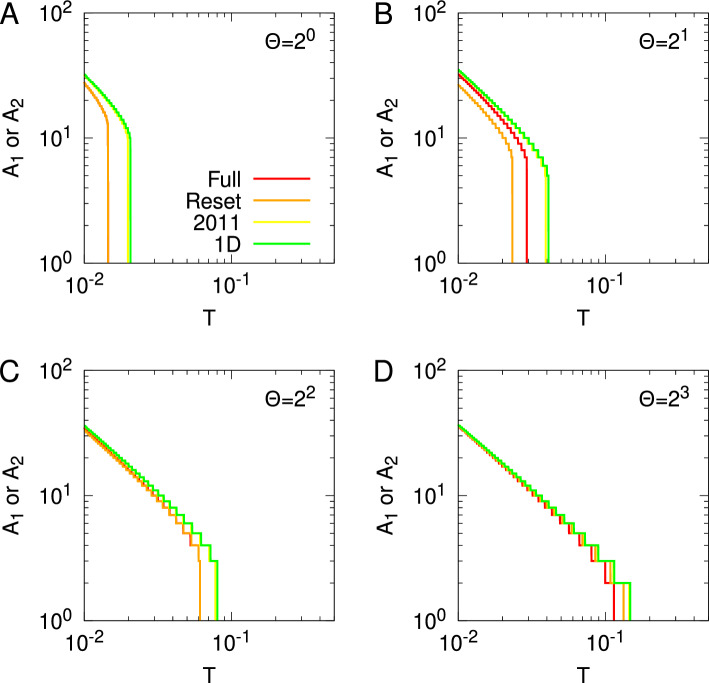
Convergence of the onset of maxima in the equilibrium probability distributions of synaptic strengths. Each panel shows the synaptic strength state $A_1 > 0$ (or $A_2 > 0$), when it exists, at which $P_{(A_1,0)} (\infty )$ (or $P_{(0, \, A_2)} (\infty )$) achieves a maximum, as a function of the plasticity step size *T*. These maxima are shown for the full model of two afferents synapsing on a target cell, and for its resetting and 2011 approximations. Also shown are the locations of the maxima in the one-dimensional distribution $P_{A;0} (\infty )$ for a single afferent synapsing on a target cell, taken from Fig. 4A. Each panel shows the four sets of results for the four different equilibrium distributions for the indicated choice of filter threshold $\varTheta$. Results for the full and resetting models in panel A are identical. Results for the 2011 model and from the one-dimensional distribution are very similar and sometimes hard to distinguish. Standard parameters are as in Fig. 8

The similarity of the two-afferent equilibrium distributions for the full, resetting and 2011 models should carry over to the locations of their maxima and the onset of these maxima through bifurcation processes. In Fig. 13, we plot the locations of the maxima as a function of the plasticity step size *T* for the three models, for different choices of $\varTheta$. We also plot the location of the maximum in the one-afferent case for comparison. Strikingly, the locations of the maxima for the one-afferent case and the 2011 model are almost identical, regardless of the value of $\varTheta$. Given that the 2011 model in Eq. (56) is an immediate generalisation of Eq. (38) for a single afferent, ignoring the complicated joint transitions in STDP and filter states of multiple afferents, perhaps this near-identity of these results for the 2011 model and the one-afferent case should not be so surprising. Given this, we might expect that this near-identity also extends to the general *m*-dimensional equilibrium distribution of the 2011 model for $m > 2$ afferents, because nothing fundamentally changes in Eq. (56) in the presence of more afferents. The locations of the maximum for the full and resetting models are also similar to those for the 2011 model and the one-afferent case. For smaller $\varTheta$, the full and resetting models do differ from the other two cases, but with larger $\varTheta$, including even just $\varTheta = 8$ in Fig. 13, the results for all four cases become very similar for most values of *T* not very close to the critical synaptic plasticity step size $T_{\textrm{C}}$ corresponding to the onset of these non-zero maxima.

**Fig. 14:**
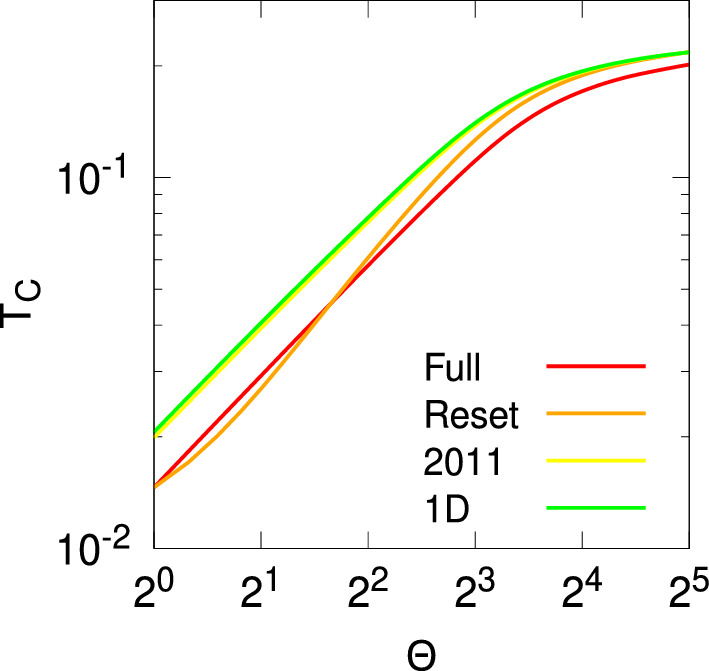
Critical synaptic plasticity step size as a function of filter threshold. Numerical results are shown for the four models indicated in the legend. Standard parameters are as in Fig. 8

As a final comparison of the different models, in Fig. 14 we plot the dependence of $T_{\textrm{C}}$ on $\varTheta$ for the three two-afferent models and the one-afferent case. As in Fig. 13, the results for the 2011 model and the one-afferent case are extremely close. This proximity validates our earlier use (Elliott 2011b) of the one-afferent case to obtain an analytical estimate of the value of $T_{\textrm{C}}$ for $\varTheta = 1$ in the 2011 model, and confirms that the result for arbitrary $\varTheta$ derived above in Eq. (54) extends to the general $\varTheta$ case of the 2011 model. The value of $T_{\textrm{C}}$ in the full model is somewhat below that in the 2011 model, but the former approaches the latter as $\varTheta$ increases, with the relative percentage difference for $\varTheta = 1$ being 31%, while for $\varTheta = 32$ it is just 8%. The value of $T_{\textrm{C}}$ in the resetting model essentially interpolates between that in the full model for smaller $\varTheta$ and that in the 2011 model for larger $\varTheta$. This interpolation is quite striking, because although in both the 2011 model and the resetting model any given filter threshold event resets all filters’ states to zero, the resetting model nevertheless retains the full two-dimensional sub-threshold filter dynamics of the full model, while the 2011 model replaces these full filter dynamics with one-dimensional filter processes for each afferent separately.

Taken together, the results in Figs. 11 to 14 indicate that the full, resetting and 2011 models all behave similarly, with the similarity increasing as $\varTheta$ increases. Furthermore, these results also indicate that the one-afferent case provides good quantitative understanding of the conditions under which segregated states emerge in the two-afferent case, where the location of an equilibrium probability maximum for some synaptic strength state $A > 0$ is used as a proxy for the presence of segregated states in the general case. To understand the similarity between these various cases, we must consider the two-dimensional filter escape process, to which we now turn.

### Filter escape processes

**Fig. 15:**
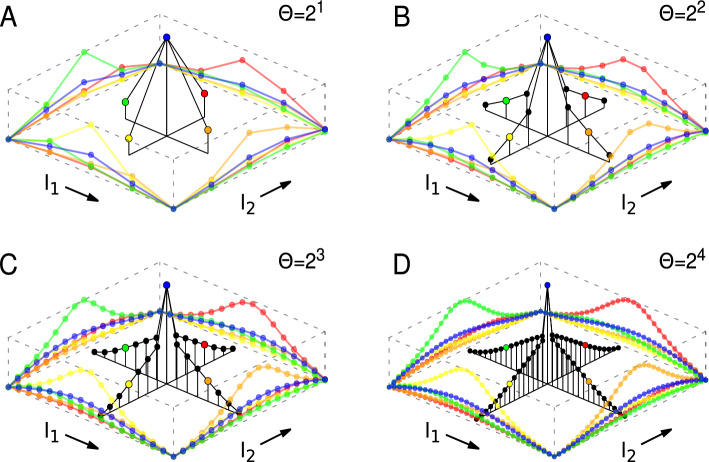
Equilibrium cross-hair filter state distribution and asymptotic filter threshold splitting probabilities. Each panel shows a schematic three-dimensional plot of the conditional equilibrium probability distribution $P_{\underline{A}; \, \underline{I}} (\infty ) / P_{\underline{A}} (\infty )$ of the cross-hair filter states $\underline{I} \in \mathfrak {X}$ for a given synaptic strength state $\underline{A}$, and the asymptotic splitting probability distributions $\pi _{\underline{I}; \, \underline{A}}^{\, \underline{\mathfrak {I}}} (\infty )$ of escape from some of these cross-hair states $\underline{I} \in \mathfrak {X}$ through all the boundary sites $\underline{\mathfrak {I}} \in \mathfrak {B}$. The equilibrium probability distribution of cross-hair filter states is represented as impulses drawn upwards from the black cross-shaped region in the square space of all possible filter states $\underline{I} = (I_1, I_2)$. These probabilities are normalised by their maximum value so that all panels have a common scale. The asymptotic splitting probability distribution of escape from some of these cross-hair states is plotted around the square region representing the filter threshold boundary $\mathfrak {B}$. These probabilities are also normalised by their collective maximum value within each panel. The splitting distributions are shown for five cross-hair filter states, distinguished by the choice of colour: $\underline{I} = (0,+\varTheta /2) \in \mathfrak {V}$ (red); $\underline{I} = (+\varTheta /2,0) \in \mathfrak {H}$ (orange); $\underline{I} = (0,-\varTheta /2) \in \mathfrak {V}$ (yellow); $\underline{I} = (-\varTheta /2,0) \in \mathfrak {H}$ (green); and $\underline{I} = (0,0) \in \mathfrak {Z}$ (blue). We take $\underline{A} = (18,18)$ with $T = 1/100$, corresponding to, or very close to, the location of the saddle in the equilibrium probability distribution of synaptic strength states (for $\varTheta = 2$ and 4 the saddle is at (17, 17), while for $\varTheta = 8$ and 16 it is at (18,18)). The choice of $\varTheta$ is indicated in each panel, and all other parameters are as in Fig. 8. The maximum conditional cross-hair state probabilities are: **A** 0.551; **B** 0.286; **C** 0.146; **D** 0.073. The maximum displayed splitting probabilities are: **A** 0.347; **B** 0.173; **C** 0.077; **D** 0.040. For comparison, the maximum escape probabilities through $(+\varTheta , +\varTheta )$ are: **A** 0.0287; **B** 0.0088; **C** 0.0026; **D** 0.0008

The equilibrium joint strength and filter state distribution is determined by the asymptotic filter escape rates $r_{\underline{I}; \, \underline{A}}^{\, \underline{\mathfrak {I}}} (\infty )$ from the cross-hair filter states $\underline{I} \in \mathfrak {X}$ through the filter boundary states $\underline{\mathfrak {I}} \in \mathfrak {B}$ for strength states $\underline{A}$. These asymptotic rates are themselves determined by the splitting probabilities $\pi _{\underline{I}; \, \underline{A}}^{\, \underline{\mathfrak {I}}}$ and the total mean escape times through any boundary site via Eq. (67). In Fig. 15 for a particular choice of strength state $\underline{A}$ with $T = 1/100$ and for different values of $\varTheta$, we plot these splitting probabilities around the filter boundary sites together with the conditional probability distribution $P_{\underline{A}; \, \underline{I}} (\infty ) / P_{\underline{A}} (\infty )$ of the cross-hair filter states for $\underline{I} \in \mathfrak {X}$. We have chosen the strength state $\underline{A} = (18, 18)$, corresponding to a location at or very close to the saddle point in the equilibrium strength distribution, but the results are very similar at or close to the segregated strength states.

Considering first the distribution of cross-hair filter states, the distribution for all choices of $\varTheta$ is concentrated around the state $\underline{I} = (0, 0)$ corresponding to the bull’s eye filter state. The probability jumps here from its neighbouring cross-hair states $(\pm 1, 0)$ and $(0, \pm 1)$ because the bull’s eye state may be reached from five different boundary sites while the other cross-hair states may each be reached from only two different boundary sites. The distribution of cross-hair filter states at this strength state $\underline{A} = (18, 18)$ is roughly-speaking symmetrical in the vertical ($\mathfrak {V}$) and horizontal ($\mathfrak {H}$) directions, and with no biasing of the distribution towards positive or negative filter states. This is true for all the strength states close to the line connecting the segregated states and the saddle node. For increasing $\varTheta$, the probability of the cross-hair states near filter thresholds becomes suppressed, with the bulk of the cross-hair probabilities being focused more towards the vicinity of the bull’s eye state.

For the splitting probabilities, we plot these around the boundary for five different choices of cross-hair filter state, including the bull’s eye state. The splitting probabilities are peaked at or near to filter state $I_1 = 0$ or $I_2 = 0$. Starting from a cross-hair state $\underline{I} \in \mathfrak {H}$, the system is biased to escape through the closest boundary site, so that the splitting probability is maximised at or close to the boundary sites $(\pm \varTheta , 0)$ for $\underline{I} = (\pm I_1, 0)$ ($I_1 > 0$, same signs). Similarly for the $\mathfrak {V}$ states. This biasing becomes more pronounced at $\varTheta$ increases in Fig. 15. Starting from the bull’s eye state $\underline{I} = (0, 0)$, roughly speaking the system is equally likely to escape in any of the four directions in filter space, but again always with a bias towards the $(0, \pm \varTheta )$ and $(\pm \varTheta , 0)$ escape sites.

As discussed above, the cross-hair filter states are central because the joint filter state always returns to a cross-hair state at filter threshold. Although the system will perform a random walk over possibly all filter states before it finally escapes through a filter boundary site, this process is already taken into account in the calculation of the splitting probabilities starting from the cross-hair states. The details of these internal filter dynamics are unimportant: what is key is that the system always returns to a cross-hair state and then exits through some boundary site to another cross-hair state. The cross-hair filter distributions and splitting probabilities in Fig. 15 therefore in some sense reinforce each other. In particular, the maximising of splitting probabilities at or near to the escape sites $(0, \pm \varTheta )$ and $(\pm \varTheta , 0)$ ensures that the joint filter state tends on average to be reset to a state that is at or close to the bull’s eye state. This observation explains why the resetting model may be used as an approximation to the dynamics of the full model. In addition, although a two-dimensional random walk never formally reduces or collapses to one-dimensional processes (unless the transition probabilities in any given dimension vanish), the tendency of the splitting probabilities to be maximised at or near to the escape sites $(0, \pm \varTheta )$ and $(\pm \varTheta , 0)$ also explains why the 2011 model approximates the resetting and full models.

Simultaneous changes in both afferents’ strength states occur only when both filters simultaneously reach threshold. Only the doubly potentiating induction process can drive such a transition in joint filter state. For the same parameters as in Fig. 15, starting from the bull’s eye state, the ratio of the splitting probability through $(+\varTheta , +\varTheta )$ to the maximum splitting probability through any boundary site is: 0.335 for $\varTheta = 1$ ($\varTheta = 1$ data are not shown in Fig. 15); 0.191 for $\varTheta = 2$; 0.128 for $\varTheta = 4$; 0.077 for $\varTheta = 8$; 0.046 for $\varTheta = 16$; 0.026 for $\varTheta = 32$ ($\varTheta = 32$ data are not shown in Fig. 15). These results confirm, as we have discussed previously (Elliott 2011b), that the filtering of induction signals decorrelates changes in synaptic strength, with the extent of the decorrelation increasing as $\varTheta$ increases. For $\varTheta = 1$, there is no filtering process, so that induced changes in synaptic strength are expressed immediately. In this case, the decorrelation is caused not by filtering but by the stochastic STDP decay processes that operate independently in each synapse. In particular, in the absence of the stochastic decay process from the UP state at rate $\gamma _+$, the only exit from the joint $\textrm{UP}_1 \textrm{UP}_2$ state would be the doubly potentiating postsynaptic-spike-driven transition back to the joint $\textrm{OFF}_1 \textrm{OFF}_2$ state. But for $\gamma _+ > 0$, the joint $\textrm{UP}_1 \textrm{UP}_2$ state may decay to $\textrm{UP}_1 \textrm{OFF}_2$ or $\textrm{OFF}_1 \textrm{UP}_2$, with a subsequent postsynaptic spike then driving only singly-potentiating transitions. In fact, the STDP decay processes underlie the decorrelation that occurs for $\varTheta > 1$, at least for the simple filter model considered here. In the presence of synaptic filtering, the random walk in filter space driven by the STDP induction processes amplifies the decorrelation already present in the STDP switch process rather than adds anything fundamentally new to it. However, for other models of filtering that introduce further stochastic decay processes, such as the molecular filter model (Elliott 2011b), the additional sources of stochasticity introduced into the filtering mechanism itself further compound rather than merely amplify the decorrelation of synaptic strength changes.

**Fig. 16:**
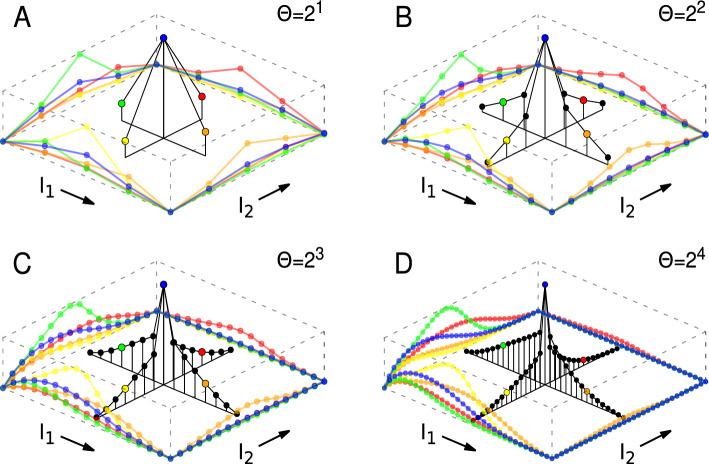
Equilibrium cross-hair filter state distribution and asymptotic filter threshold splitting probabilities. The format of this figure is identical to Fig. 15, but we have taken an arbitrary strength state of $\underline{A} = (10,90)$ rather than the saddle strength state of $\underline{A} = (18,18)$. The maximum conditional cross-hair state probabilities are: **A** 0.556; **B** 0.295; **C** 0.161; **D** 0.097. The maximum displayed splitting probabilities are: **A** 0.389; **B** 0.211; **C** 0.108; **D** 0.061. For comparison, the maximum escape probabilities through $(+\varTheta , +\varTheta )$ are: **A** 0.0177; **B** 0.0034; **C** 0.0003; **D** 0.0000 ($9 \times 10^{-6}$)

Along the line connecting the segregated states and the saddle node, potentiation and depression processes are balanced. This is because competitive dynamics arise principally in the $S_- = S_1 - S_2$ direction (for the parameters used here), while in the orthogonal, $S_+ = S_1 + S_2$ direction, the dynamics quickly move the system close to the line $S_+ = \mathscr {S}_+$ (or $A_1 + A_2 \approx \mathscr {S}_+ / T$, since the $A_i$ must be integers). Above this line, depression dominates, while below it, potentiation dominates; on it, they are matched. To illustrate the domination of depression for synaptic strength states satisfying $S_1 + S_2 > \mathscr {S}_+$, in Fig. 16 we plot the splitting probabilities and cross-hair filter distribution for the point $\underline{A} = (10, 90)$ (with $T = 1/100$), which is well away from the central core of the equilibrium probability distribution on which competitive dynamics occur. In comparison to Fig. 15, we see that the distribution of cross-hair filter states is biased towards negative values, $I_1 < 0$ or $I_2 < 0$, with this biasing becoming clearer for larger choices of $\varTheta$. The splitting probabilities are themselves also biased towards escape through the lower rather than upper filter thresholds, and their maxima are somewhat skewed away from the threshold states $(0, \pm \varTheta )$ and $(\pm \varTheta , 0)$. In these regions of synaptic strength state space above the central core of the equilibrium strength probability distribution, the dynamics are dominated by what is essentially a relaxation process as the afferents rapidly fall through their available strength states towards the central core. Both afferents fall equally rapidly, unless one moves close to its minimum strength state. If we instead choose a joint strength state below the central core (data not shown), then the domination of potentiation in this region is reflected in the biasing of splitting probabilities towards escape through upper rather than lower filter thresholds, although the distribution of cross-hair filter states is not so biased as in the depression case. The (relative) probability of escape through $(+\varTheta , +\varTheta )$ leading to simultaneous changes in synaptic strength remains suppressed in this region.

### Simulation results

We now turn to simulation results. Given the structure of the equilibrium probability distributions illustrated above, with their very rapid transitions from the high probability cores around the segregated states to the much lower probability saddle node, which is increasingly suppressed as $\varTheta$ increases, attempts to obtain decent numerical samples of these distributions from simulation would be futile. Although we could obtain the overall shape of a distribution, it would be too noisy in simulation to be able to make meaningful comparisons to analytical or numerical results. The same is true of the lifetimes of the quasi-stable segregated states, especially when these lifetimes are anything other than very short. Although the MSI $\langle | \langle S_I \rangle | \rangle$ is in many respects a rather implicit or even coarse indicator of states’ lifetimes, it is robust and relatively easy to obtain from simulation with good averaging.

#### Mean segregation index from simulations

**Fig. 17:**
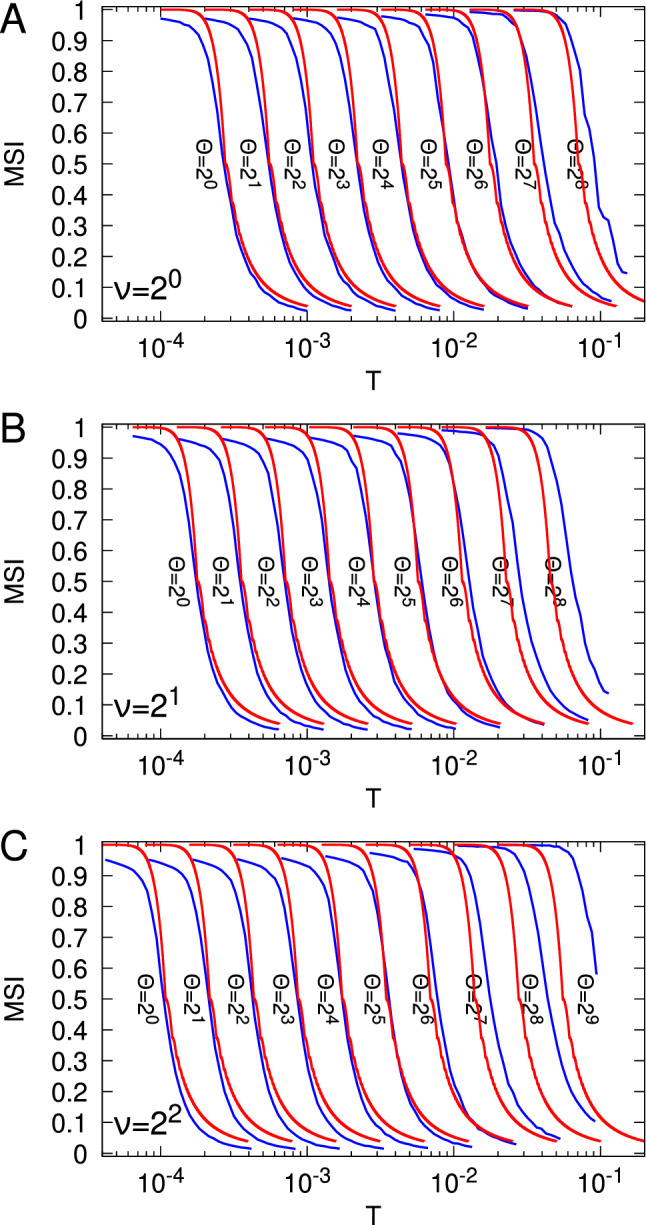
Mean segregation index as a function of plasticity step size *T*. Simulation results are shown in blue while analytical results are shown in red. Results are shown for different choices of filter threshold $\varTheta$, indicated immediately to the left of the corresponding simulation curve. All panels show results for $m=2$ afferents synapsing on a single target cell, but the different panels show results in which each afferent supports a different number $\nu$ of distinct synapses, as indicated in each panel. The analytical results for the MSI in panel A for $\nu = 2^0$ synapses per afferent are obtained from the simple model discussed in relation to Eq. (95). The analytical results in panels B and C are obtained from the same analytical formula, but the value of *T* is reduced by a factor of 1.55 and 2.55, respectively, to align the $\varTheta = 1$ analytical and simulation results in both these panels. All simulation parameters are identical to those used to obtain the numerical results shown in earlier figures

In Fig. 17A, we plot results for the MSI obtained from simulation for various choices of filter size $\varTheta$ as indicated, together with the analytical form for the MSI obtained from the simple model discussed in Sect. 3.5, which permits the analytical result for $\tau _{\textrm{stab}}$ in Eq. (88) to be converted into a corresponding MSI. The simulation results in Fig. 17A are similar to but extend and significantly improve on those presented earlier (Elliott 2011b, Fig. 3): here we average over 1000 simulations rather than just 80 to obtain much better statistics; consider more and larger choices of $\varTheta$; use a slightly longer probe periods of $1.25 \times 10^7$ s rather than $10^7$ s. Overall, we see that the analytical results for the MSI in Fig. 17A qualitatively match the simulation results very well. For smaller $\varTheta$ and thus smaller *T*, the saturation dynamics do differ somewhat, but this is largely because in the definition of $\tau _{\textrm{stab}}$, we employed a strict definition of a segregated state as simply either (*A*, 0) or (0, *A*) for some value of *A*. However, typically the segregated states are not sharp. Instead, one afferent fluctuates around its zero strength state, with only large, rare fluctuations inducing a transition from a state near (*A*, 0) to a state near (0, *A*), or *vice versa*. However, in the simple model for the MSI, we just took the segregation index $S_I = S_- / S_+$ to take values of $\pm 1$ (exact, perfect segregation) for a fixed duration of $\tau _{\textrm{stab}}$. Both the assumed perfect segregation and the fixed duration of $\tau _{\textrm{stab}}$ will affect the saturation dynamics for segregated states.

For larger choices of $\varTheta$ and thus larger values of *T*, however, we see that the saturation dynamics change, becoming sharper with the MSI approaching unity more rapidly as $\varTheta$ increases. This is because for larger $\varTheta$, the saturation of the MSI occurs at larger *T*. For larger *T*, the spacing between neighbouring strengths increases and the system occupies fewer strength states. In a segregated state, therefore, the losing afferent does exactly occupy the zero strength state rather than hovers around it: for larger *T*, a fluctuation from the zero strength state will take the afferent’s synaptic strength to a large value, and thus $S_I$ will be close to zero. These dynamics also explain why, in Fig. 17A, we have not taken *T* above around 0.2. For the parameters used here, the total equilibrium synaptic strength of both afferents is around 0.35. For large *T*, a segregated strength state can be (1, 0) or (0, 1), or (2, 0) or (0, 2), so very close to (0, 0). Fluctuations can then take segregated states to the (0, 0) state, in which both afferents’ strength states are zero. Such a state is not permanently absorbing for $\lambda _{\textrm{spont}} > 0$ Hz, but clearly $S_I = S_- / S_+$ cannot be defined, and is in any event meaningless, in such a disconnected state. Moreover, as *T* increases, it approaches the critical value $T_{\textrm{C}}$, above which the probability maxima away from the origin cease to exist. In all models considered above, $T_{\textrm{C}}$ is around 0.2 at most, for the parameters used here. Hence, for *T* above this value, segregated states do not exist. This does not mean that $\varTheta$ cannot be taken higher than the largest value of 256 used in Fig. 17A. Rather, we have not shown results for larger values because either they lack the low MSI tail as *T* increases up to $T_{\textrm{C}}$ or their associated MSIs are always near unity for all values of $T < T_{\textrm{C}}$. Even for $\varTheta = 256$ in Fig. 17A, the tail of the simulation MSI curve is truncated.

We observe the scaling behaviour of the simulation results for the MSI with $\varTheta$, so that as $\varTheta$ doubles in Fig. 17A, the simulation MSI curve shifts rightward with the corresponding value of *T* also doubling. This scaling only starts to break down as $\varTheta$ approaches around 100. Earlier we saw that the analytical and numerical results for $T_{\textrm{C}}$ also exhibit scaling for smaller values of $\varTheta$, up to around $\varTheta = 8$ (see Eq. (54) and Figs. 4B, 14), but that this scaling breaks down for larger $\varTheta$ for which $T_{\textrm{C}}$ exceeds around 0.1. It is striking, then, that in the simulation results in Fig. 17A, the scaling only starts to break down for much larger values of $\varTheta$, roughly an order of magnitude larger than in the results for $T_{\textrm{C}}$. The analytical result for $\tau _{\textrm{stab}}$ in Eq. (88), and thus the MSI calculated from it, is obtained from a Fokker–Planck equation, which entails a continuum approximation in which *T* is assumed to be small. We commented earlier that the equilibrium distribution from the Fokker–Planck fails to be a satisfactory approximation to that from the exact master equation when *T* reaches around 0.1. We see that the breakdown of scaling in Fig. 17A is consistent with the requirement that the Fokker–Planck equation requires *T* to be at most around 0.1: for $\varTheta = 128$, the threshold-like region of the MSI curve is starting to approach the $T = 0.1$ region at its lower end, so that the Fokker–Planck results must start to break down there. The value of $\varTheta$ at which scaling starts to break down is therefore simply determined by the relevant value of *T* for $\varTheta = 1$. For $\varTheta = 1$, MSI = $\frac{1}{2}$ at $T \approx 2.7 \times 10^{-4}$ (marginally lower than the value obtained from the one-dimensional case) while $T_{\textrm{C}} \approx 1.5 \times 10^{-2}$ (in the one-dimensional case, $T_{\textrm{C}} \approx 2.1 \times 10^{-2}$). Thus, the semi-saturation location of the MSI curve is much lower than $T_{\textrm{C}}$ and hence the MSI results exhibit scaling for larger values of $\varTheta$ than do those for $T_{\textrm{C}}$.

#### Multiple synapses per afferent

In our earlier work (Elliott 2011b), we tacitly assumed that a single afferent supports just a single synaptic contact of (discretely) variable synaptic strength on a target neuron. Here, we have thus far continued to make this tacit assumption, equivocating between “afferent” and “synapse” as if the two are interchangeable terms. In reality, while a single afferent by definition can support only a single synaptic connection on a single target cell, this entire connection typically consists of axonal branches that ramify and make multiple (often hundreds or perhaps thousands of) individual synaptic contacts (separate, distinct synaptic boutons) on the dendrites of a target cell. Because an afferent’s synapses usually cannot distinguish on which target cells they form contacts except through the pattern of postsynaptic spikes, and similarly because a target cell usually cannot distinguish between the different afferents synapsing on it except through the patterns of presynaptic spikes at each synaptic contact, we must assume that each separate, individual synaptic contact possesses the biomolecular machinery to implement our postulated STDP tristate switch (Appleby and Elliott 2005). Indeed, this assumption is crucial for the model’s ability to explain the usual biphasic, graded STDP curve (Bi and Poo 1998) for pre- and postsynaptic spike pairs of arbitrary time difference. Ignoring issues such as the branch point failure of axonal spike propagation and dendritic calcium spike back-propagation, although all the synapses between an afferent and a target cell will experience identical patterns of pre- and postsynaptic spikes, the decorrelating effects of the multiple synaptic tristate switches’ stochastic decay processes will ensure that all these synapses do not change their strengths in an identical manner. Each individual synapse, for any afferent, must therefore also implement a synaptic filter to control the fluctuations in synaptic strength to which its own STDP switch gives rise.

For the case in which an afferent supports a single synapse, we have seen that its plasticity step size *T* critically determines whether or not segregated states exist and their quasi-stability. If we consider that an afferent supports $\nu > 1$ synaptic contacts on the target cell, then potentially any number of these $\nu$ synapses may undergo simultaneous changes in strength. The plasticity step size *T* per synapse then changes into an effective plasticity step size over the entire synaptic connection, where this effective size fluctuates depending on the decorrelation induced by the STDP switch mechanism and its subsequent amplification by synaptic filtering. We would therefore expect to see the simulation MSI curves in Fig. 17A for $\nu = 1$ synapse per afferent shifting leftward, towards smaller values of *T*, when $\nu$ increases. The remaining panels in Fig. 17, panel B with $\nu = 2$ and panel C with $\nu = 4$, confirm this expectation. In order to re-align the $\nu = 1$ and $\varTheta = 1$ analytical results in panel A to the simulation results for $\nu > 1$, we must reduce *T* by a factor of 1.55 and 2.55 in panels B and C, respectively; for $\nu = 8$, we must reduce *T* by a factor of 4.41 (data not shown).

**Fig. 18:**
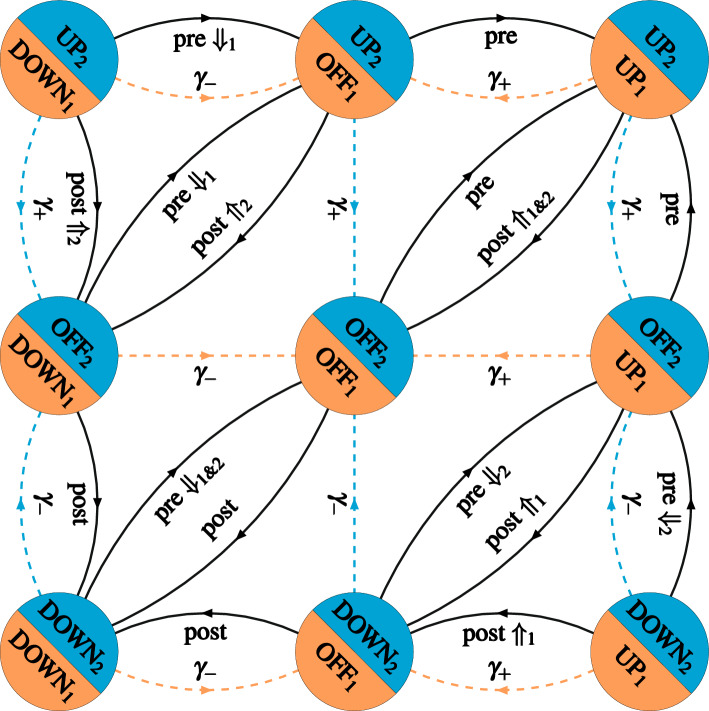
Joint synaptic state transitions induced by spike-timing-dependent plasticity in the tristate switch model for two synapses supported by the same afferent synapsing on the same target cell. Both synapses experience the same sequence of presynaptic spikes (now shown with the same black directed lines rather than lines of different colours) as well as the same sequence of postsynaptic spikes. Different colours continue to be used to show the separate, independent stochastic decay processes in each synapse’s STDP states. The format of this figure is otherwise identical to that of Fig. 5, except that we do not show the marginal transitions for each synapse as they are identical to those in Fig. 5 when $\hbox {pre}_1$ and $\hbox {pre}_2$ are replaced by pre

For $\varTheta = 1$, we can calculate the average effective plasticity step size for a single afferent supporting $\nu$ synapses, at least for smaller values of $\nu$. Figure 18 illustrates the transitions in joint STDP tristate switch states for $\nu = 2$ synapses, both experiencing the same sequence of pre- and postsynaptic spikes. Figure 18 differs from Fig. 5 for two afferents each of one synapse because in the former, both synapses experience common presynaptic spikes rather than the latter’s uncorrelated presynaptic spikes. The common presynaptic spikes in the former drive the diagonal presynaptic transitions, and in particular the transition from the joint $\textrm{DOWN}_1 \textrm{DOWN}_2$ state to the joint $\textrm{OFF}_1 \textrm{OFF}_2$ state, which gives rise to a doubly depressing process in which both synapses depress simultaneously. With the usual asymptotic approximation, we can use the equilibrium distribution $\underline{\sigma }$ of these joint STDP states (same ordering of states as above) to determine the probabilities of singly and doubly potentiating or depressing processes, conditional on their being a potentiating or depressing process. The average effective potentiating and depressing plasticity step sizes are then given by

97a $$\begin{aligned} T_{\textrm{eff}}^+&= \frac{(0,0,1,0,0,1,1,1,2)^{\textrm{T}} \cdot \underline{\sigma }}{(0,0,1,0,0,1,1,1,1)^{\textrm{T}} \cdot \underline{\sigma }} \; T, \end{aligned}$$

97b $$\begin{aligned} T_{\textrm{eff}}^-&= \frac{(2,1,1,1,0,0,1,0,0)^{\textrm{T}} \cdot \underline{\sigma }}{(1,1,1,1,0,0,1,0,0)^{\textrm{T}} \cdot \underline{\sigma }} \; T, \end{aligned}$$

respectively, where the vectors in the numerators count the number of plasticity steps (of the required type) in the afferent’s two synapses, while those in the denominators provide the conditioning on the occurrence of plasticity steps (of the required type). The calculation can be performed in general for $\nu$ synapses, but is limited by the exponential growth in the number, $3^\nu$, of joint STDP states of $\nu$ synapses. Using $\lambda _\pi = 50$ Hz for simplicity (i.e. $\varsigma = 0$ Hz) and standard parameters, $T_{\textrm{eff}}^\pm$ are then functions of $\lambda _p$. Choosing for concreteness the values of $\lambda _p$ at which $T_{\textrm{eff}}^+ = T_{\textrm{eff}}^-$ so that potentiation and depression are matched (around $\lambda _p = 20$ Hz, depending on $\nu$), we find that $T_{\textrm{eff}}^\pm / T = 1.44$ for $\nu = 2^1$, 2.30 for $\nu = 2^2$, and 3.98 for $\nu = 2^3$. These numbers are not too dissimilar to the factors of 1.55, 2.55 and 4.41 by which *T* must be reduced in order to align the $\nu = 1$ analytical MSI curves with those from simulation for $\nu > 1$.

These $\varTheta = 1$ results for the effective plasticity step size provide good qualitative understanding of the leftward shift of the MSI curves in Fig. 17 for $\nu > 1$. However, for $\varTheta > 1$ the effective plasticity step sizes $T_{\textrm{eff}}^\pm$ must reduce because of the filter-induced amplification of the decorrelation of plasticity steps caused by the STDP stochastic decay processes. For $\nu = 2$, we can repeat the above calculation of $T_{\textrm{eff}}^\pm$ for $\varTheta > 1$ using the asymptotic distribution of filter states to obtain the rates of single and double plasticity steps. For $\varTheta = 2^1$, we find that $T_{\textrm{eff}}^\pm / T = 1.22$; for $\varTheta = 2^2$, $T_{\textrm{eff}}^\pm / T = 1.09$; for $\varTheta = 2^3$, $T_{\textrm{eff}}^\pm / T = 1.04$; for $\varTheta = 2^4$, $T_{\textrm{eff}}^\pm / T = 1.01$; and for $\varTheta = 2^5$, $T_{\textrm{eff}}^\pm / T = 1.01$ (actually 1.005). The general $\nu$ and general $\varTheta$ calculation becomes very difficult if not intractable because of the number of joint STDP and filter states, but for example for $\nu = 3$ and general $\varTheta$, it is clear that single plasticity steps occur when the system escapes through the faces of the cube that defines the joint filter state $(I_1, I_2, I_3)$; double plasticity steps occur only through the edges of this cube; and triple plasticity steps only through two of its vertices. Although increasing $\nu$ increases the possible multiplicity of plasticity steps, it becomes overwhelmingly more likely that the system will escape through the vastly more numerous hyper-faces of the hyper-cube defining the filter space, corresponding to only a single synapse changing strength. Thus, although the results for $T_{\textrm{eff}}^\pm$ provide a good account of the simulation MSI results for $\varTheta = 1$, they cannot explain the results for general $\varTheta$.

To do so, we must re-derive the Fokker–Planck equation in Eq. (85) from the 2011 model’s master equation in Eq. (56) for $\nu > 1$ synapses per afferent. We write $S_{i; a}$ for the strength of synapse $a = 1, \ldots , \nu$ of afferent *i*, with $\underline{S} = ( S_{1; 1}, \ldots , S_{1; \nu }, S_{2; 1}, \ldots , S_{2; \nu } )$. We then define $S_\pm = \left( \sum _a S_{1; a} \right) \pm \left( \sum _a S_{2; a} \right)$ and $2 (\nu - 1)$ further, linearly independent or orthogonal variables, call them $X_j$, $j = 1, \ldots , 2 (\nu - 1)$, forming a vector $\underline{X}$. For example, for $\nu = 2$ we can take $X_{1,2} = (S_{1; 1} - S_{1; 2}) \pm (S_{2; 1} - S_{2; 2})$. We normalise these additional variables so that they have the same normalisation as $S_\pm$ (i.e. if $X_j = \underline{v} \cdot \underline{S}$, then $\underline{v} \cdot \underline{v} = 2 \nu$). Summing Eq. (56) over all synapses just as it sums over all afferents, the Fokker–Planck equation in Eq. (85) then always takes the form

98a $$\begin{aligned} \frac{\partial P(S_-, \underline{X}; t)}{\partial t}&= \nu \frac{\partial }{\partial S_-} \left[ U' (S_-) P(S_-, \underline{X}; t) \right] \nonumber \\&\quad + \nu \vartheta \left( \frac{\partial ^2}{\partial S_-^2} + \sum _{j=1}^{2(\nu -1)} \frac{\partial ^2}{\partial X_j^2} \right) P(S_-, \underline{X}; t), \end{aligned}$$

to the same level of approximation. In particular, we have restricted to the $S_+ = \mathscr {S}_+$ hyper-plane so that derivative terms in $S_+$ are dropped; the deterministic dynamics contain derivative terms only in $S_\pm$ with that in $S_+$ being dropped, with $U(S_\pm , \underline{X})$ being calculated to order $( \varsigma / \mu )^2$ and depending only on $S_\pm$ with $S_+$ being fixed; the diffusion terms are calculated to order $( \varsigma / \mu )^0$, with all the cross-derivative terms vanishing or cancelling at this order. The potential $U(S_-)$ and diffusion constant $\vartheta$ are then unchanged from Eq. (85). Despite the fact that Eq. (98a) involves $2 \nu - 1$ variables, its coefficients depend only on $T / \varTheta$, and so scaling again automatically emerges, at this level of approximation, for any $\nu$. There are no drift terms in Eq. (98a) involving the $X_j$ variables. This is because the postsynaptic firing rate $\lambda _p$ depends only on $\sum _a S_{1; a} = (S_+ + S_-)/2$ and $\sum _a S_{2; a} = (S_+ - S_-)/2$. The synaptic plasticity expression rates therefore depend only on $S_\pm$ and hence the drift terms do not involve derivatives in the $X_j$ variables. At the level of the deterministic dynamics, the $X_j$ variables therefore do not evolve, remaining at their initial values. Their full dynamics include the diffusion terms in Eq. (98a), so these variables simply diffuse around their initial values. We see precisely this behaviour in simulation. As the $X_j$ variables represent dynamics orthogonal to $S_-$, they do not affect the MFPT for transitions between segregated states. Ignoring them, Eq. (98a) then differs from Eq. (85) only in an additional overall factor of $\nu$ on the right-hand side. This overall factor simply rescales *t*, and therefore $\tau _{\textrm{stab}}$ is reduced by a factor of $\nu$. However, this reduction has relatively little impact on the MSI in the regime that $\tau _{\textrm{stab}}$ grows exponentially fast, because of the saturation of the MSI in this regime. Thus, Eq. (98a) correctly accounts for scaling (dependence only on $T / \varTheta$), but it does not give the correct leftward shift of the MSI curves for $\nu > 1$ even for $\varTheta = 1$.

This failure reflects the fact that we have simply summed Eq. (85) over all $2 \nu$ synapses, treating them separately and independently. Although we must continue to treat the two afferents in this way to maintain the 2011 model’s analytical tractability, treating each afferent’s $\nu$ synapses in this way explicitly ignores the possibility that some of them can undergo simultaneous changes in strength. Thus, we must extend Eq. (56) to include all possible simultaneous intra-afferent changes in synaptic strength state, but we must continue to exclude any simultaneous inter-afferent changes to maintain the spirit of the 2011 model’s approximation. We may then write down the corresponding Fokker–Planck equation in the $S_\pm$ and $\underline{X}$ variables. Because the Fokker–Planck equation is sensitive only to first- and second-order statistics, it can only reflect strength change processes involving one or two synapses, so at most doubly potentiating or depressing processes. The drift term must remain unchanged because it involves only single processes, so double processes can only arise in the diffusion terms. Eq. (98a) then becomes

98b $$\begin{aligned} \frac{\partial P(S_-, \underline{X}; t)}{\partial t}&= \nu \frac{\partial }{\partial S_-} \left[ U' (S_-) P(S_-, \underline{X}; t) \right] \nonumber \\&\quad + \nu ^2 \vartheta _2 \frac{\partial ^2}{\partial S_-^2} P(S_-, \underline{X}; t) \nonumber \\&\quad + \nu \vartheta _1 \left( \frac{\partial ^2}{\partial S_-^2} + \sum _{j=1}^{2(\nu -1)} \frac{\partial ^2}{\partial X_j^2} \right) P(S_-, \underline{X}; t), \end{aligned}$$

where

99a $$\begin{aligned} \vartheta _1&= \left( \frac{T}{\varTheta } \right) ^2 \big [ r^+ (\mu , \mu \mathscr {S}_+) + r^- (\mu , \mu \mathscr {S}_+) \nonumber \\&\hspace{1.75cm} - r^{++} (\mu , \mu \mathscr {S}_+) - r^{--} (\mu , \mu \mathscr {S}_+) \big ], \end{aligned}$$

99b $$\begin{aligned} \vartheta _2&= \left( \frac{T}{\varTheta } \right) ^2 \left[ r^{++} (\mu , \mu \mathscr {S}_+) + r^{--} (\mu , \mu \mathscr {S}_+) \right] , \end{aligned}$$

where $r^{++} (\lambda _\pi , \lambda _p)$ and $r^{--} (\lambda _\pi , \lambda _p)$ are the asymptotic rates of the doubly potentiating and depressing processes that arise from the intra-afferent, two-synapse STDP induction processes in Fig. 18. The overall diffusion constant in the $S_-$ variable is now $\nu ( \vartheta _1 + \nu \vartheta _2)$, and the MFPT lifetime $\tau _{\textrm{stab}}$ of segregated states is given by Eq. (88) under the replacements $\varLambda \rightarrow \nu \varLambda$ and $\vartheta \rightarrow \nu ( \vartheta _1 + \nu \vartheta _2)$. We then find that the resulting analytical MSI curves shift leftward, with *T* reducing by a factor of 1.65 for $\nu = 2^1$, 2.94 for $\nu = 2^2$, and 5.51 for $\nu = 2^3$. These numbers should be compared to those from simulation (1.55, 2.55 and 4.41, respectively), and from the $T_{\textrm{eff}}^\pm$ calculation for $\varTheta = 1$ (1.44, 2.30 and 3.98, respectively). While the $T_{\textrm{eff}}^\pm$ argument somewhat underestimates the leftward shift, the results for $\tau _{\textrm{stab}}$ from the revised Fokker–Planck equation somewhat overestimate the leftward shift, but both sets of results are consistent with the simulation results given the various approximations involved.

The leftward shift of the MSI curves with $\nu$ for any $\varTheta$ owes its origin to the $\nu (\nu - 1)$ pairs of possible simultaneous strength changes in both afferents. In particular, there are $\nu (\nu - 1)/2$ pairs of possible simultaneous updates within an afferent’s synapses, although as $\varTheta$ increases the probability of any particular simultaneous update decreases. These possible simultaneous updates within an afferent’s synapses can destabilise the other afferent’s connection to the target cell, perhaps ultimately leading to a transition in macroscopic connectivity from one segregated state to the other. These possible changes represent a contribution to fluctuations and therefore increase the diffusion constant, with contributions from both afferents. As $\nu$ increases, *T* must be decreased in order to maintain the same lifetime of segregated states, shifting the MSI curves leftward.

## Discussion

Over a couple of decades or so, we have tried to develop a view of single synapses as computationally rather simple devices with relatively limited biomolecular resources in which to represent or instantiate elaborate computations. The often tacit assumption that the synaptic plasticity rule displayed by any entire synaptic connection between afferent and target cells should be embodied at the level of any of its single synaptic contacts has therefore seemed to us ripe for dissection. We have thus sought to offload the computational burden imposed on single synapses by this assumption onto emergent computations over the ensemble of synapses, exploiting temporal and/or spatial averaging. Our model of STDP as driven by a simple, tristate switch mechanism in which a single synapse changes its strength in discrete steps with only a very minimal form of spike coincidence detection has been informed by this view. Synaptic filtering is then required to control the fluctuations to which any stochastic or probabilistic approach is inevitably subject.

Our previous work (Elliott 2011b) argued that our proposed STDP switch mechanism tends to decorrelate the changes in synaptic strengths of different afferents, and synaptic filtering either amplifies this decorrelation or provides additional sources of decorrelation, depending on the details of the filter model. This argument lent plausibility to although did not validate the approach taken in the 2011 model, in which afferents’ contributions to the master equation (and hence the Fokker–Planck equation) essentially sum independently. This massively simplifying assumption was key to our earlier analysis, permitting a generality that would otherwise have been impossible. This assumption provides critical analytical insight (in good agreement with simulation) into the lifetimes of quasi-stable patterns of synaptic connectivity. It therefore permits the interpretation of the plasticity step size as a temperature-like parameter, and it then establishes the role of synaptic filtering as a mechanism for effectively rescaling the plasticity step size. Validating this approximation by demonstrating why and when it works well, and showing that it is in good agreement with results obtained without it, has been essential. Essential not only for our own models, but also for those models of synaptic plasticity in general where this, or related approximations, may be made.

To validate and shed further light on our 2011 model, here we have considered the full dynamics of two afferents synapsing on a single target cell. The full model does not employ the resetting approximation, which is a renewal approximation that allows a filter threshold event in one afferent to reset all afferents’ filters to the zero state. Nor does it replace two-dimensional (or in general multi-dimensional) filter escape densities with one-dimensional densities, which was an additional, then-unrecognised approximation in the 2011 model. The results from all three models are similar, in terms of their equilibrium distributions and even their critical plasticity step sizes. These results therefore confirm the validity of the 2011 model, and our examination of the distribution of cross-hair filter states and their escape densities demonstrates that the decorrelation of synaptic strength changes does indeed underlie this validity.

Furthermore, the results of all three models are also similar to those from a purely one-dimensional model, where the existence of a maximum of the equilibrium probability distribution away from the zero strength state is taken as a surrogate (or a necessary condition) for the existence of segregated states in the general case. Of course, a necessary condition is not a sufficient condition: we do not claim that the existence of a probability maximum away from the zero strength state for a single afferent guarantees (is a sufficient condition for) the presence of competitive dynamics between multiple afferents. Indeed, our STDP model exhibits parameter regimes in which multiple afferents can have non-zero strengths without competition between them (Elliott 2008). Rather, the existence of segregated patterns of neuronal connectivity involving multiple afferents at least requires that each afferent has access to a non-zero maximum of the joint strength state probability distribution away from the zero strength state. A one-afferent system will therefore also have access to such a state if a multi-afferent system has, particularly when competitive dynamics are in fact present, because once segregated, a target cell has by definition reduced to a one-afferent system. What is striking is that a purely one-afferent system’s non-zero strength and critical plasticity step size are similar to those for a two-afferent system, and thus the former may act, analytically-speaking, as a surrogate for at least some aspects of the latter. However, given that the full and 2011 models’ results are so similar, and that the 2011 model essentially reduces the synaptic dynamics of multiple afferents down to one-dimensional systems coupled only through their common postsynaptic firing rate, perhaps the ability of a purely one-afferent system to capture key properties of a multi-afferent system should not be so surprising, even while it remains striking. Although we cannot of course use a purely one-afferent system to understand the regimes in which competitive dynamics arise between multiple afferents, we can then at least use this very much simpler system to understand some of the central principles uncovered by the 2011 model, such as the existence of a critical plasticity step size and the role of synaptic filtering.

The approach taken in our earlier work (Elliott 2011b) permitted considerable generality, obtaining as far as possible generic results for any induction and expression processes. Unfortunately, that level of generality has been impossible here, because of the largely intractable nature of the two-dimensional (or in general multi-dimensional) random walks generated by our STDP model. Reliance on numerical methods has therefore been essential: we can write down master and Fokker–Planck equations, but we can only solve them numerically. That even numerical solutions continue to be possible reflects the advantages offered by our approach to synaptic plasticity in general and to STDP in particular. In contrast, in conventional models of STDP it is in general essentially impossible to examine the impact of fluctuations in any detail, because conventional models of STDP enforce correlated changes in synaptic strengths driven by postsynaptic spiking (see, for example, Kempter et al. 1999; van Rossum et al. 2000; Rubin et al. 2001; Burkitt et al. 2004). We have discussed these issues at length elsewhere (Elliott 2011b), so we do not rehearse them here.

Most of our present and all of our earlier (Elliott 2011b) study made the conventional (and convenient) assumption that we may represent the entire synaptic connection between an afferent and a target cell by a single synaptic strength. However, an afferent’s synaptic connection in fact consists of many individual synaptic contacts, each of which according to our model of STDP must implement a tristate STDP switch and therefore also a mechanism to implement synaptic filtering. Our simulation results show that the results for $\nu > 1$ synapses per afferent are essentially identical to those for the $\nu = 1$ case. In particular, they exhibit the same scaling properties, with the MSI (and therefore the lifetimes of segregated states) depending only on the ratio $T / \varTheta$, at least over a range of parameters. The only substantial difference between the $\nu > 1$ and $\nu = 1$ cases is that the MSI curves are shifted leftward in a plot of MSI against *T* for $\nu > 1$ compared to $\nu = 1$. For $\varTheta = 1$, we can interpret this shift as due to the effective plasticity step size. For general $\varTheta$, the leftward shift arises because any of the $\nu (\nu - 1)/2$ pairs of an afferent’s synapses may undergo simultaneous strength changes, even while the probability of such simultaneous changes decreases with increasing $\varTheta$.

The leftward shift of the MSI curves with increasing $\nu$ raises two potential issues. First, for $\varTheta = 1$, the value of *T* at which the MSI is semi-saturated clearly reduces, and perhaps it reduces to a level that we should regard as unrealistically small for biologically relevant values of $\nu$. Second, the concomitant issue arises as to whether $\varTheta$ must then be taken implausibly large in order to restore *T* to a reasonable level. For general $\nu$, assuming that an afferent’s synapses on any given target cell are all of similar strength, the relevant comparison is between $\mathscr {S}_+ / \nu$ and *T* rather than between $\mathscr {S}_+$ and *T*, where $\mathscr {S}_+$ is the total synaptic strength of the winning, segregated afferent. For larger $\nu$, we would expect that the step change in strength per synapse should be smaller precisely because each synapse contributes, on average, only fractionally to the overall synaptic connection. What constitutes *T* being implausibly small then becomes a relative question. Indeed, if we try to take *T* close to $T_{\textrm{C}}$ by taking $\varTheta$ large enough (whether plausible or not), then the winning afferent’s synapses will not contribute roughly equally to the entire synaptic connection: only at most a handful of them will have non-zero strength, and the rest will have zero strength. The strength diffusion amongst an afferent’s synapses that we discussed above may mean that an afferent will constantly reconfigure its overall connection with the target cell, but this regime is likely just an irrelevant modelling artefact. Thus, we would expect *T* to take a value that is not too small relative to $\mathscr {S}_+ / \nu$ nor so close to $T_{\textrm{C}}$ that the very concept of $\nu$ becomes essentially meaningless.

Whether or not $\varTheta$ must then be taken to be implausibly large is unclear. We make, however, a couple of remarks. First, the model of filtering that we have considered here is in many respects the simplest and weakest of those that we have considered previously. The “molecular filter model” (Elliott 2011a), which posits an additional stochastic decay process that returns the filter state to zero, controls fluctuations more powerfully, using smaller filter thresholds to achieve the same mean escape times as the model without decay considered here. The so-called “super filter” (Elliott 2016) enforces the requirement that $\varTheta$ consecutive induction signals of the same type are required to achieve threshold, with any intervening induction signal of the opposite type immediately resetting the filter to zero. The escape times in the super filter grow exponentially rather than polynomially with $\varTheta$, providing an extremely powerful control of fluctuations. Thus, other filter models are available, and may not require $\varTheta$ to be taken as large as for the simplest model considered here. Second, when we estimated the lifetimes of segregated states above for $\nu > 1$, we ignored spike propagation branch point failures, considering just the simplest scenario in which all synapses between the same afferent cell and the same target cell experience identical sequences of pre- and postsynaptic spikes. However, spike propagation failures will reduce the correlations in the spike trains experienced by synapses, lowering the probability of simultaneous changes in synaptic strengths between synapses. This will reduce the diffusion constant in the Fokker–Planck equation and attenuate the leftward shift of the MSI curves as $\nu$ increases. Any increase in $\varTheta$ to shift the MSI curves rightward would then be correspondingly attenuated. Spike propagation failure may then, on this view, be a feature rather than a flaw.

We have made two other approximations: the asymptotic and adiabatic approximations. The former is justified by the rapidity with which the STDP switch states reach (close to) their asymptotic distribution. The latter is a standard approximation made in the stochastic modelling literature to separate processes occurring on different time scales (see, e.g. Gardiner 1985; van Kampen 1992). Given that we have seen that considering just a single afferent is sufficient to capture the key dynamics present even in a multi-afferent system, it is possible that we may even be able to relax these two approximations and consider the exact dynamics of a single afferent synapsing on a single target cell. This might allow us to probe issues around the duration of the firing epochs analytically and determine how such epochs interact with the emergence of segregated states (or at least probability maxima) and their stability. It will be fascinating to pursue this possibility in future work.

## Data Availability

Available upon request.

## Footnotes

1 We have previously referred to the UP and DOWN states as “POT” and “DEP” states, respectively. Unfortunately this risked some confusion, with the possibility that, for example, the POT state was incorrectly regarded as a state in which a synapse is potentiated.

2 We use $\delta _a^b$, $\delta _{a,b}$ and $\delta ^{a,b}$ to denote the same Kronecker delta symbol, where the positions of the indices match those in the associated transition probabilities in any given context. From a tensor perspective, this is an abuse of notation, but we use this convention for greater clarity.

3 For example, for $\varTheta = 32$ the numerical calculation of all the joint filter escape probabilities and rates for a single joint strength state takes several minutes. This calculation must be repeated for all strength states, so that with $T = 1/200$ and setting a maximum strength state of $N = 200$, there are $201^2 = 40,401$ such calculations. Even in a cluster environment, this involves a few weeks of calculations for all the filter escape processes alone.

## Code availability

Available upon request.
